# From methylglyoxal to pyruvate: a genome-wide study for the identification of glyoxalases and D-lactate dehydrogenases in *Sorghum bicolor*

**DOI:** 10.1186/s12864-020-6547-7

**Published:** 2020-02-10

**Authors:** Bidisha Bhowal, Sneh L. Singla-Pareek, Sudhir K. Sopory, Charanpreet Kaur

**Affiliations:** 1 International Centre for Genetic Engineering and Biotechnology (ICGEB), Aruna Asaf Ali Marg, New Delhi, 110067 India; 2 School of Life Sciences, Jawaharlal Nehru University, New Delhi, 110067 India

**Keywords:** D-lactate dehydrogenase, Genome-wide analysis, Glyoxalase, Sorghum, Stress

## Abstract

**Background:**

The glyoxalase pathway is evolutionarily conserved and involved in the glutathione-dependent detoxification of methylglyoxal (MG), a cytotoxic by-product of glycolysis. It acts via two metallo-enzymes, glyoxalase I (GLYI) and glyoxalase II (GLYII), to convert MG into D-lactate, which is further metabolized to pyruvate by D-lactate dehydrogenases (D-LDH). Since D-lactate formation occurs solely by the action of glyoxalase enzymes, its metabolism may be considered as the ultimate step of MG detoxification. By maintaining steady state levels of MG and other reactive dicarbonyl compounds, the glyoxalase pathway serves as an important line of defence against glycation and oxidative stress in living organisms. Therefore, considering the general role of glyoxalases in stress adaptation and the ability of *Sorghum bicolor* to withstand prolonged drought, the sorghum glyoxalase pathway warrants an in-depth investigation with regard to the presence, regulation and distribution of glyoxalase and D-LDH genes.

**Result:**

Through this study, we have identified 15 *GLYI* and 6 *GLYII* genes in sorghum. In addition, 4 *D-LDH* genes were also identified, forming the first ever report on genome-wide identification of any plant D-LDH family. Our in silico analysis indicates homology of putatively active SbGLYI, SbGLYII and SbDLDH proteins to several functionally characterised glyoxalases and D-LDHs from *Arabidopsis* and rice. Further, these three gene families exhibit development and tissue-specific variations in their expression patterns. Importantly, we could predict the distribution of putatively active SbGLYI, SbGLYII and SbDLDH proteins in at least four different sub-cellular compartments namely, cytoplasm, chloroplast, nucleus and mitochondria. Most of the members of the sorghum glyoxalase and D-LDH gene families are indeed found to be highly stress responsive.

**Conclusion:**

This study emphasizes the role of glyoxalases as well as that of D-LDH in the complete detoxification of MG in sorghum. In particular, we propose that D-LDH which metabolizes the specific end product of glyoxalases pathway is essential for complete MG detoxification. By proposing a cellular model for detoxification of MG via glyoxalase pathway in sorghum, we suggest that different sub-cellular organelles are actively involved in MG metabolism in plants.

## Background

Methylglyoxal (MG) was initially identified as a physiological growth inhibiting substance owing to its biological effects [1]. Subsequent studies established MG as a ubiquitous reactive dicarbonyl compound present under physiological as well as stress conditions. MG is primarily synthesised through non-enzymatic reactions as a by-product of various metabolic pathways including carbohydrate, protein and fatty acid metabolism [2–4]. Of these, glycolytic pathway remains the most important endogenous source of MG [5]. Further, reactions catalysed by enzymes such as, monoamine oxidase (MAO), cytochrome P450 (CP450) and MG synthase (MGS), can also synthesize MG using substrates derived from amino acids, fatty acids and glucose metabolism, respectively [6].

**Fig. 1:**
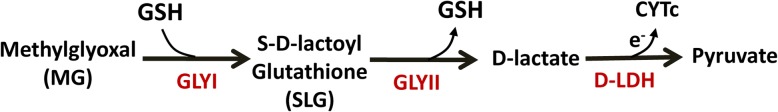
Schematic representation of the glyoxalase pathway for methylglyoxal detoxification in plants. Methylglyoxal (MG) is converted to S-D-lactoylglutathione (SLG) by glyoxalase I (GLYI) enzyme which is then converted to D-lactate by glyoxalase II (GLYII). Glutathione is used in the first reaction catalysed by GLYI but is recycled in the second reaction catalysed by GLYII. D-lactate is further metabolized to pyruvate through D-lactate dehydrogenase (D-LDH) enzyme which passes electrons to cytochrome C (CYTc)

MG being a potent glycating agent can readily react with lipids, proteins and nucleic acids forming advanced glycation end products (AGEs) in turn, rendering its accumulation highly deleterious for the cell as it leads to subsequent cell death [7]. Among the various MG detoxification mechanisms reported so far, the glyoxalase system is considered to be the major route for its detoxification and other reactive dicarbonyl compounds in the living systems (Fig. 1). It plays a crucial role in cellular defence against glycation and oxidative stress [7–9]. In plants, depending on glutathione (GSH) requirement, the MG detoxifying enzymes can be classified as GSH-dependent or GSH-independent. Glyoxalase pathway is the GSH-dependent system which detoxifies MG via a two-step enzymatic reaction, catalysed by glyoxalase I (GLYI, lactoylglutathione lyase) and glyoxalase II (GLYII, hydroxyacylglutathione hydrolase) enzymes. Here, the first step involves a spontaneous reaction between MG and GSH to form hemithioacetal (HTA), which is then isomerized to S-D-lactoylglutathione (SLG) by GLYI. In the second step, GLYII hydrolyzes SLG to release D-lactate and thus, recycles one GSH molecule into the system. In addition to the GSH-dependent glyoxalase system, there also exists a shorter GSH-independent, direct pathway for MG detoxification which has recently been reported in rice [10]. The enzyme involved is glyoxalase III, also known as DJ-1 protein due to its high sequence similarity with human DJ-1 protein (HsDJ-1). In humans, DJ-1 proteins are associated with early onset of Parkinson disease and it was only later that the presence of glyoxalase III activity was reported in such proteins [11]. The catalytic mechanism of this enzyme is completely different from the typical two-step glyoxalase pathway, as it neither requires GSH nor metal cofactors for activity [10].

D-lactate, which is the product of MG detoxification catalyzed by either GLYI-GLYII system or GLYIII enzymes, is then further metabolised to pyruvate via D-lactate dehydrogenases (D-LDH) and thus, D-lactate formation can be termed as the final step in the MG detoxification pathway (Fig. 1). In fact, D-LDH links MG degradation with the electron transport chain through Cytochrome c (CYT c). In *Arabidopsis*, CYT*c* loss-of-function mutants and the D-LDH mutants, are sensitive to both D-lactate and MG, indicating that they function in the same pathway. On the other hand, over-expression of either of the two viz. D-LDH or CYT*c,* increases tolerance of the transgenic plants to D-lactate and MG [12]. Further, GLYI and D-LDH from *Arabidopsis* have been shown to confer tolerance to various abiotic stresses in both prokaryotes and eukaryotes [13]. In rice, silencing of D-LDH impedes glyoxalase system leading to MG accumulation and growth inhibition [14].

The production of MG in response to various environmental cues and its subsequent detoxification by the glyoxalase pathway, together with its ability to trigger a widespread plant response, makes MG and glyoxalases suitable biomarkers for stress tolerance [15]. A large volume of evidence resulting from in vivo and in silico studies has revealed MG to be a central metabolite controlling signal transduction, gene expression and protein modification [16, 17]. To date, several genome-wide analyses have been carried out that located the presence of multiple glyoxalase isoforms in all the plant species studied. A total of 11 *GLYI* and 5 *GLYII* genes in *Arabidopsis thaliana* [18], 11 *GLYI* and 3 *GLYII* in *Oryza sativa* [18], 24 *GLYI* and 12 *GLYII* in *Glycine max* [19], 29 *GLYI* and 14 *GLYII* in *Medicago truncatula* [20] and, 16 *GLYI* and 15 *GLYII* in *Brassica rapa* [21] have been identified. Very recently, 4 *GLYI* and 2 *GLYII* genes encoding putative functionally active glyoxalase isoforms have also been identified in grapes [22]. Similarly, a recent comparative analysis of glyoxalases genes in *Erianthus arundinaceus* and a commercial sugarcane hybrid has led to the identification of 9 *GLYI* and 7 *GLYII* genes in sugarcane, with the wild cultivar showing higher expression of glyoxalase genes under stress conditions than the commercial variety [23].

The existence of multiple forms of these enzymes indicates the presence of possibly different reaction mechanisms, regulations and their tissue-specific distribution across plant species, thereby suggesting several important physiological functions for these enzymes in plants. Few recent studies have in fact highlighted altogether different roles of glyoxalases in plants i.e. in pollination [24] and starch synthesis [25].

*Sorghum bicolor* (L.) Moench is truly a versatile crop that can be grown as a grain, forage or sweet crop. It is among the most efficient crops with regard to its ability to convert solar energy and also in use of water and thus, is known as a high-energy, drought-tolerant crop [26]. Owing to sorghum’s wide uses and adaptation, it is considered “one of the really indispensable crops” required for the survival of humankind (see Jack Harlan, 1971). Notably, sorghum is of interest to the US DOE (Department of Energy) as a bio-energy crop because of its resilience to drought and its ability to thrive on marginal lands. Since glyoxalases are important for stress adaptation in plants and since sorghum has remarkably high capacity to resist drought, we thought it pertinent to investigate the presence, regulation and distribution of glyoxalases in sorghum.

Towards this, in the present study, we carried out a genome-wide analysis of MG detoxification genes viz. *GLYI*, *GLYII* and *D-LDH*, in sorghum. Our results indicate the presence of 15 *GLYI*, 6 *GLYII* and 4 *D-LDH* genes in the sorghum genome with multiple members co-localising in mitochondria, chloroplast and cytoplasm. Of these, cytoplasm and mitochondria could be said to possess complete MG detoxification pathway, as the functionally active *GLYI*, *GLYII* and *D-LDH* genes could be predicted to exist in these sub-cellular compartments. However, while chloroplasts have been predicted to possess functional GLYI and GLYII, it is predicted to not possess any D-LDH protein. Further, we observed development and tissue specific variations in the expression of these three gene families. Though several similar studies have been carried out in other plant species, those have mainly focused on the first two enzymes of the pathway. We believe that D-LDHs are equally important for the complete detoxification of MG as D-lactate is exclusively formed from the reactions of glyoxalase enzymes. Future studies may focus on elucidating the physiological functions of these different forms with respect to both MG detoxification and various developmental processes in plants.

## Results

### Identification and analysis of glyoxalase genes in sorghum

The Hidden Markov Model (HMM) profile search for conserved glyoxalase domain (PF00903 and PF12681) led to the identification of 15 putative *SbGLYI* genes, of which 6 genes, *SbGLYI-1*, *SbGLYI-7*, *SbGLYI-8*, *SbGLYI-9*, *SbGLYI-10* and *SbGLYI-11*, were found to have varying transcript lengths (Table 1). Among these, *SbGLYI-1* and *SbGLYI-8* were predicted to form alternatively spliced products. As a result, a total of 17 SbGLYI proteins were identified in sorghum. However, PCR-based assessment of spliced variants of *SbGLYI-7*, *SbGLYI-8*, *SbGLYI-10* and *SbGLYI-11* genes using primers designed from the coding sequence (CDS) or 5′ or 3′- untranslated region (UTR), revealed several discrepancies. Amplicon of expected size was obtained only for *SbGLYI-8* transcript thereby, validating the presence of two spliced variants (Additional file 1: Figure S1). However, no spliced variant could be detected for *SbGLYI-10* and *SbGLYI-11* genes. In contrast, we failed to PCR amplify *SbGLYI-7* gene and as a result could not validate the presence or absence of spliced variants of this gene (Additional file 1: Figure S1).

**Table 1:** List of putative glyoxalase I genes present in *Sorghum bicolor*

Gene Name	Locus Name	Transcripts	Coordinate (5′-3′)	Transcript length (bp)	CDS (bp)	Protein			Localisation
						Length (aa)	MW (kDa)	pI	
*SbGLYI-1*	Sobic.001G147300	Sobic.001G147300.1	11,834,277..11836939	810	429	142	15.18	5.75	Cytoplasm
		Sobic.001G147300.2	11,833,232..11837989	705	501	166	15.18	5.75	Chloroplast
*SbGLYI-2*	Sobic.001G418500	Sobic.001G418500.1	69,928,671..69929611	859	420	139	15.3	4.96	Cytoplasm
*SbGLYI-3*	Sobic.002G104200	Sobic.002G104200.1	12,324,224–12,326,388	1596	1491	496	52.48	5.78	Chloroplast
*SbGLYI-4*	Sobic.002G401400	Sobic.002G401400.1	75,190,443..75191454	938	633	210	15.28	4.96	Cytoplasm
*SbGLYI-5*	Sobic.003G049700.	Sobic.003G049700.1	4,550,859..4555912	2910	702	233	25.13	6.16	Cytoplasm
*SbGLYI-6*	Sobic.004G053700	Sobic.004G053700.1	4,365,877–4,367,586	1725	1323	440	46.83	5.41	Chloroplast
*SbGLYI-7*	Sobic.004G127600	Sobic.004G127600.1	15,708,207..15711117	2128	1041	346	37.8	5.84	Chloro_Mito^a^
		Sobic.004G127600.2	15,708,207..15711117	1956	1041	346	37.8	5.84	Chloro_Mito^a^
*SbGLYI-8*	Sobic.006G029800	Sobic.006G029800 .1	6,293,014..6306287	1484	684	227	25.65	7.76	Chloro_Mito^a^
		Sobic.006G029800.2	6,293,014..6306287	1445	645	214	24.2	7.77	Chloro_Mito^a^
*SbGLYI-9*	Sobic.006G162100	Sobic.006G162100.1	51,989,823..51991102	1048	525	174	19.1	5.53	Cytoplasm
		Sobic.006G162100.2	51,989,824..51990812	669	525	174	19.1	5.53	Cytoplasm
*SbGLYI-10*	Sobic.007G069000	Sobic.007G069000.1	7,692,587..7699005	2277	873	290	32.23	6.02	Cytoplasm
		Sobic.007G069000.2	7,694,823..7699442	2512	873	290	32.23	6.02	Cytoplasm
		Sobic.007G069000.3	7,694,141..7698415	1815	873	290	32.23	6.02	Cytoplasm
		Sobic.007G069000.4	7,695,376..7698415	1641	873	290	32.23	6.02	Cytoplasm
		Sobic.007G069000.5	7,692,635..7698734	1751	873	290	32.23	6.02	Cytoplasm
		Sobic.007G069000.6	7,692,588..7698415	2395	873	290	32.23	6.02	Cytoplasm
		Sobic.007G069000.7	7,694,002..7699432	2262	873	290	32.23	6.02	Cytoplasm
*SbGLYI-11*	Sobic.007G069200	Sobic.007G069200.1	7,703,151..7706621	1487	885	294	32.91	5.45	Cytoplasm
		Sobic.007G069200.2	7,703,151..7706610	1464	885	294	32.91	5.45	Cytoplasm
*SbGLYI-12*	Sobic.008G188600	Sobic.008G188600.1	62,295,706–62,300,530	2089	569	188	20.22	7.01	Chloro_Mito^a^
*SbGLYI-13*	Sobic.009G063301	Sobic.009G063301.1	6,734,003..6738618	1687	660	219	23.35	5.95	Chloroplast
*SbGLYI-14*	Sobic.009G085200	Sobic.009G085200.1	14,378,045..14386395	1419	1065	354	38.91	6.49	Chloro_Mito^a^
*SbGLYI-15*	Sobic.010G046400	Sobic.010G046400.1	3,614,012..3616367	903	369	122	13.4	6.89	Mitochondria

^a^*Chloro_mito* Chloroplast and/or mitochondria (as very similar scores for both)

The chromosomal locations, orientations and CDS length of *SbGLYI* genes along with their various physico-chemical properties and sub-cellular localisation have been listed in Table 1. SbGLYI proteins were predicted to be localised in different cell organelles. While majority of them localised in the cytoplasm and chloroplast, others were predicted to be localised both in the chloroplast and mitochondria. Only SbGLYI-15 protein was predicted to be exclusively localised in the mitochondria. Interestingly, one of the SbGLYI protein namely, SbGLYI-8 and its isoform SbGLYI-8.1, were found to harbour nuclear localisation signals (NLS) as well and therefore, may even localise in the nucleus. To further confirm, SbGLYI-8/8.1 sequences were aligned to their closest rice (OsGLYI-8) and *Arabidopsis* (AtGLYI-2) orthologs. Both SbGLYI-8 and SbGLYI-8.1 were found to possess a 20 aa long NLS near the N-terminus of the protein, as also observed in OsGLYI-8 and AtGLYI-2.4 proteins (Additional file 2: Figure S2). The predicted iso-electric points (pI) of SbGLYI proteins were found to range between 5 to 7 with a few exceptions, as for SbGLYI-2 and SbGLYI-4, which had pI lesser than 5.

Similarly, HMM profile search for metallo-beta lactamase (PF00753) and HAGH_C (PF16123) domains led to the identification of 7 SbGLYII proteins encoded by 6 *SbGLYII* genes. Similar to SbGLYI proteins, several SbGLYII proteins were also predicted to be both chloroplast- and mitochondria-localised. Two out of 7 proteins were predicted to be cytoplasmic and only one was predicted to be solely localised in the chloroplast. The predicted iso-electric points (pI) of SbGLYII proteins ranged between 5 to 8 (Table 2).

**Table 2:** List of putative glyoxalase II genes present in *Sorghum bicolor*

Gene	Locus Name	Transcripts	Coordinate (5′-3′)	Transcript length (bp)	CDS (bp)	Protein			Localisation
						Length (aa)	MW (kDa)	pI	
*SbGLYII-1*	Sobic.001G008500	Sobic.001G008500.1	815,344–818,189	2428	2088	695	77.21	6.28	Chloroplast
*SbGLYII-2*	Sobic.001G020000	Sobic.001G020000.1	1,663,460–1,671,583	3412	2217	738	81.87	5.17	Cytoplasm
		Sobic.001G020000.2	1,663,460–1,671,583	3406	2212	736	81.63	5.17	Cytoplasm
*SbGLYII-3*	Sobic.001G383100	Sobic.001G383100.1	67,068,087–67,072,709	1273	777	258	28.63	5.8	Cytoplasm
*SbGLYII-4*	Sobic.002G264400	Sobic.002G264400.1	64,894,221–64,897,742	2642	1011	336	36.95	8.05	Chloro_Mito^a^
*SbGLYII-5*	Sobic.003G249900	Sobic.003G249900	58,818,725–58,822,144	1990	891	296	32.11	8.57	Chloro_Mito^a^
*SbGLYII-6*	Sobic.004G356100	Sobic.004G356100	68,349,193–68,352,497	1410	999	332	37.33	6.32	Chloro_Mito^a^

^a^*Chloro_mito* Chloroplast and/or mitochondria (as very similar scores for both)

### Phylogenetic analysis of glyoxalase proteins of sorghum and other plant species

**Fig. 2:**
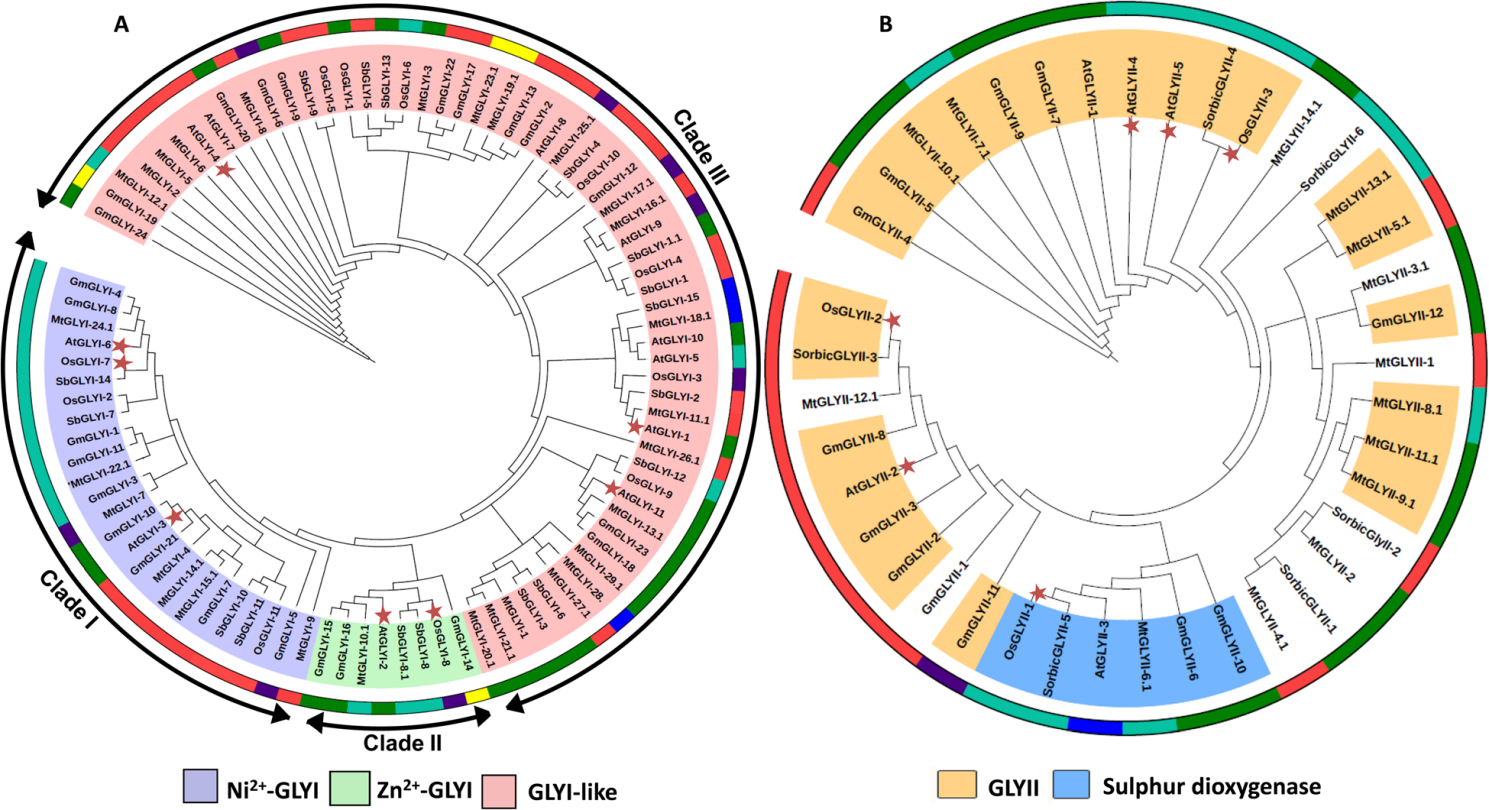
Phylogenetic analysis of glyoxalase proteins from sorghum and other plant species. Circular tree constructed for the (**a**) GLYI and (**b**) GLYII proteins from sorghum, rice, *Arabidopsis*, *Medicago* and Soybean using Neighbour-Joining method in MEGA 7.0 with 1000 bootstrap replicates. The putative sub-cellular localisation of the proteins has been indicated as rings bordering the tree in different colours. Cytoplasm (red), Chloroplast (green), Mitochondria (blue), Nucleus (purple), Extracellular/peroxisomes (yellow), Chloroplast or Mitochondria (turquoise). The localisation of those marked with asterisk have been experimentally proven

In order to study the evolutionary divergence of glyoxalase proteins, amino acid sequences of the putative SbGLYI and SbGLYII proteins were aligned to members of the well-characterised rice glyoxalase family. Sequence alignments revealed high similarity between SbGLYI and OsGLYI proteins and between SbGLYII and OsGLYII proteins. For instance, SbGLYI-7, SbGLYI-10, SbGLYI-11 and SbGLYI-14 clustered with OsGLYI-2, OsGLYI-7 and OsGLYI-11 whereas SbGLYI-8 and SbGLYI-8.1 were found to be more similar to OsGLYI-8 (Additional file 3: Figure S3). Likewise, SbGLYII-3 and SbGLYII-4 were more similar to rice OsGLYII-2 and OsGLYII-3, respectively, whereas SbGLYII-5 was closer to OsGLYII-1 in sequence (Additional file 4: Figure S4). Next, a phylogenetic tree was generated using Neighbour-Joining method for GLYI proteins from different plant species such as *Arabidopsis*, rice, soybean and *Medicago* (Fig. 2). The tree revealed clustering of proteins into three major groups, comprising of putative Ni^2+^-dependent proteins (Clade I), putative Zn^2+^-dependent GLYI proteins (Clade II) and functionally diverse GLYI-like proteins (Clade III) (Fig. 2a). Clade-III was the most populous cluster followed by Clade I and II. SbGLYI-7, SbGLYI-10, SbGLYI-11 and SbGLYI-14 clustered in the same clade as that of the previously characterised and functionally active, AtGLYI-3 and AtGLYI-6 from *Arabidopsis* and OsGLYI-2, OsGLYI-7, and OsGLYI-11 proteins from rice, with all these proteins belonging to the Ni^2+^-dependent GLYI category of proteins, whereas SbGLYI-8 grouped with Zn^2+^-dependent GLYI proteins from *Arabidopsis* (AtGLYI-2) and rice (OsGLYI-8). Overall, these GLYI protein encoding genes were predicted to be orthologous, and functionally similar. The third cluster contained greater number of proteins which have probably diverged in their functions and hence, were named as GLYI-like proteins [27].

In the case of GLYII proteins, two different subfamilies were observed in the phylogenetic tree, those with conserved active site motifs and therefore, enzymatically active and the other comprising of proteins which did not show conservation of active site residues. Of these, some were previously reported to possess sulphur dioxygenase (SDO) activity. It could be clearly seen from the tree that SbGLYII-3 shared more similarity to OsGLYII-2, and SbGLYII-4 was closer to OsGLYII-3 (Fig. 2b). Both OsGLYII-2 and OsGLYII-3 are functionally active GLYII proteins and therefore, SbGLYII-3 and SbGLYII-4 were also predicted to be enzymatically active. Further, we found SbGLYII-5 to be most similar to OsGLYII-1 and thus, was more likely to possess SDO activity (Fig. 2b).

### Gene structure analysis of sorghum glyoxalase genes

**Fig. 3:**
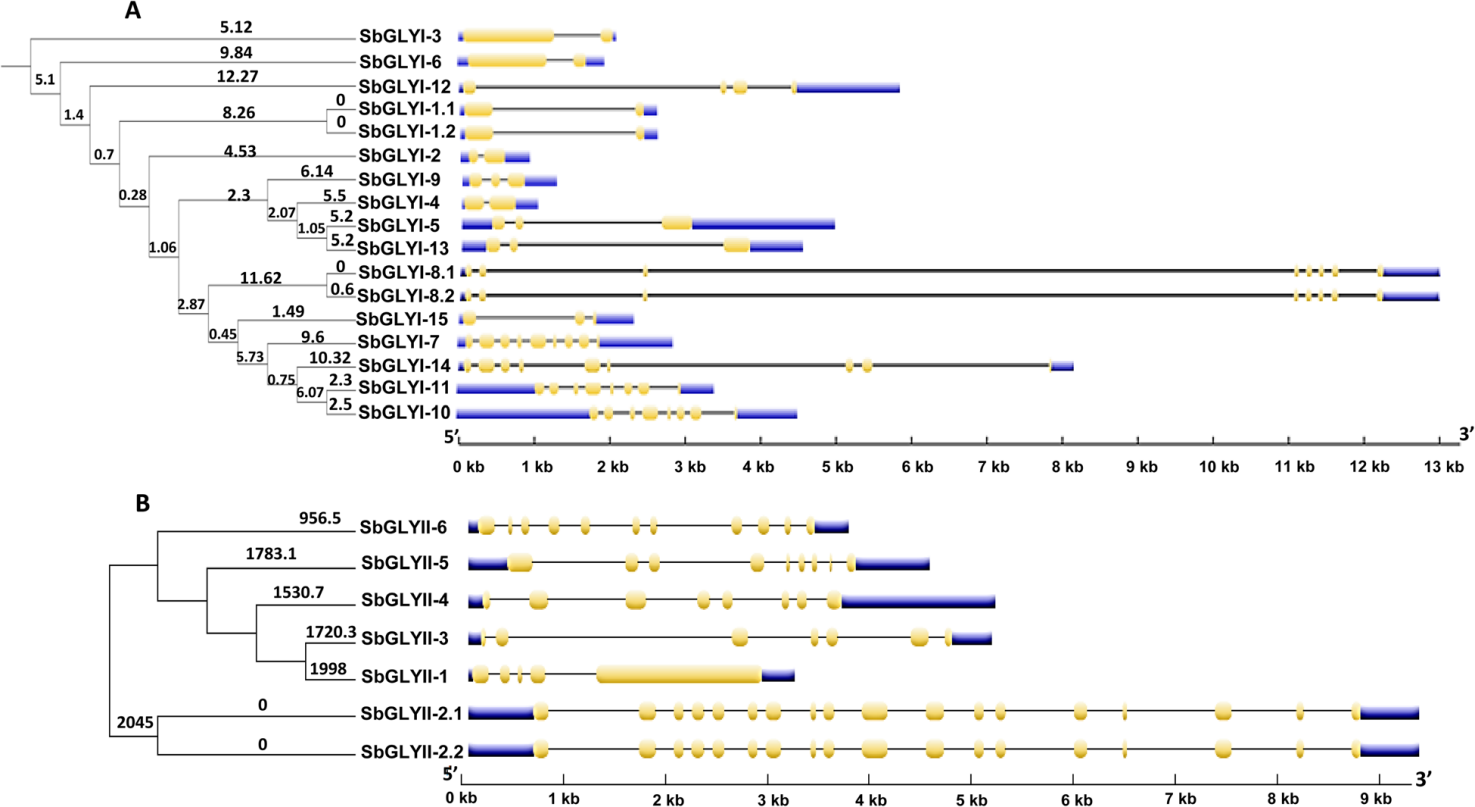
Exon-intron organisation of glyoxalase gene family from sorghum. Exon-Intron structure of (**a**) *SbGLYI* and (**b**) *SbGLYII* genes were analysed using the Gene Structure Display Server tool. Length of exons and introns has been exhibited proportionally as indicated by the scale on the bottom. Order of GLY genes is represented as per their phylogenetic relationship. The branch lengths represent evolutionary time between the two nodes

Subsequent to phylogenetic analysis and prediction of the type of GLYI and GLYII activities in the sorghum GLY proteins, we analysed their gene structure to investigate any possible correlation of gene structure with their activity. For this, exon–intron structure of the genes was drawn using the Gene Structure Display Server tool [28]. The *SbGLYI* genes predicted to be functionally active as glyoxalases, shared similar exon-intron patterns among themselves. For instance, *SbGLYI-7*, *SbGLYI-8* and *SbGLYI-14* shared 8 exons and 7 introns each, while *SbGLYI-10* and *SbGLYI-11* shared 7 exons and 6 introns. Interestingly, GLYI-like protein encoding genes which clustered into two groups according to their sequence homology, also shared similarities in their gene structure within each cluster. First cluster comprising of genes, *SbGLYI-1*, *SbGLYI-2*, *SbGLYI-3*, *SbGLYI-4* and *SbGLYI-6* uniformly shared 2 exons and 1 intron each while the other cluster comprising of genes, *SbGLYI-5*, *SbGLYI-9* and *SbGLYI-13*, shared 3 exons and 2 introns each (Fig. 3a). However, SbGLYII protein encoding genes did not show such characteristic exon-intron arrangements (Fig. 3b). *SbGLYII-3* and *SbGLYII-4* genes predicted to possess GLYII activity, consisted of 7 exons-6 introns and 8 exons-7 introns-based gene organization, respectively, whereas *SbGLYII-5* predicted to be an SDO enzyme, consisted of 9 exons and 8 introns. Among the *SbGLYII* genes, *SbGLYII-2* had the highest number of exons with both the spliced forms having 18 exons and 17 introns each (Fig. 3b).

### Domain architecture analysis of putative glyoxalases

**Fig. 4:**
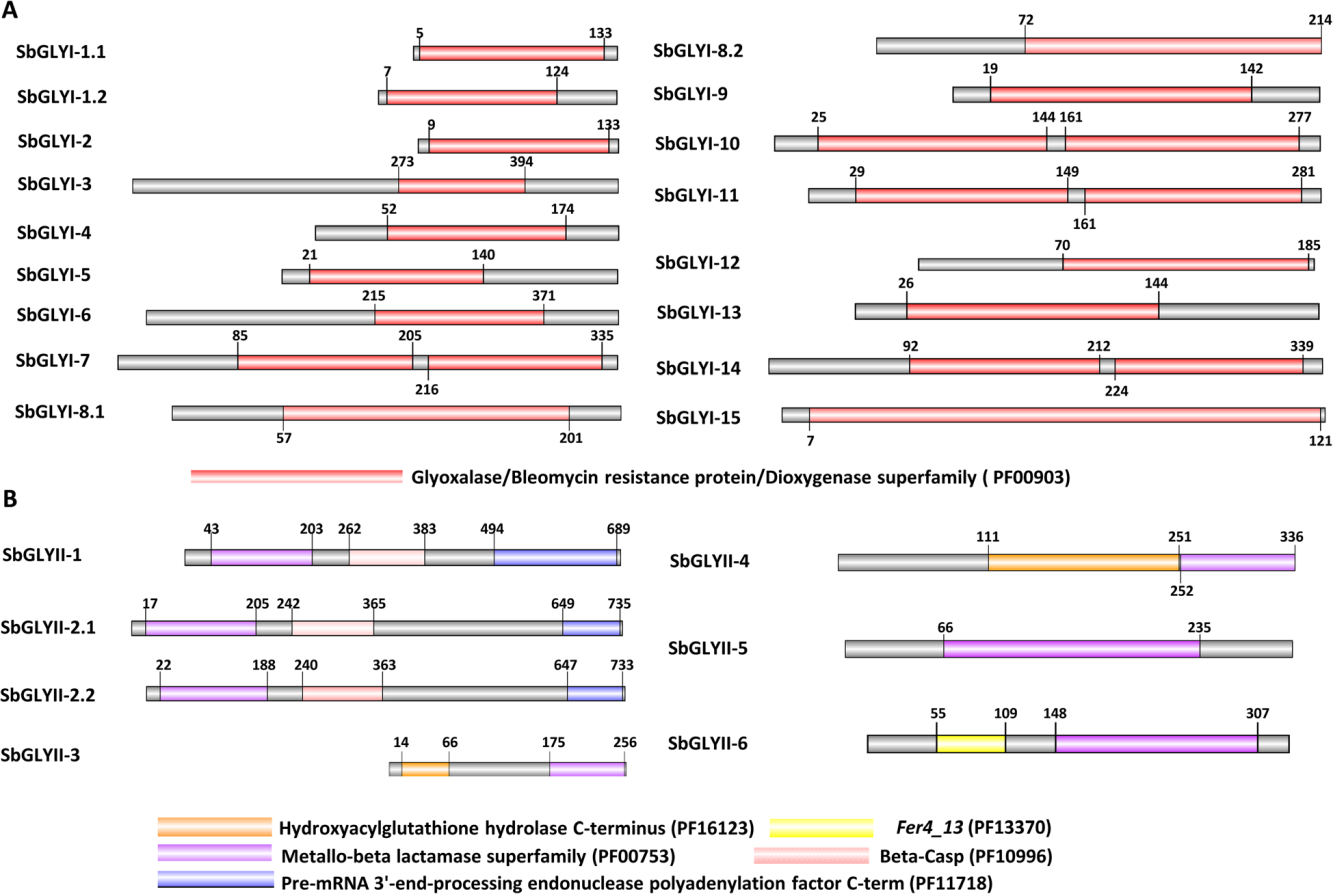
Schematic representation of domain architecture of glyoxalase proteins from sorghum. Domain architecture of (**a**) SbGLYI proteins showing the presence of glyoxalase domain (PF00903) and (**b**) SbGLYII proteins containing metallo-beta lactamase superfamily domain (PF00753) in all the predicted SbGLYII proteins. In addition, HAGH_C (PF16123) domain predicted to be important for the catalytic activity of SbGLYII proteins, was also found in some SbGLYII protein sequences while few SbGLYII proteins had other secondary domains. Domains were analysed using Pfam database. Exact position and number of domains are schematically represented along with the length of the protein

Domain architecture of putative SbGLYI proteins was analysed to determine the presence of functional domains and to draw similarities in protein features between glyoxalases from sorghum and other plant species. Analysis revealed that all the 17 SbGLYI proteins possessed only one type of domain viz. Glyoxalase/Bleomycin resistance protein/Dioxygenase (PF00903) domain. However, 4 GLYI proteins namely, SbGLYI-7, SbGLYI-10, SbGLYI-11 and SbGLYI-14 had two glyoxalase domains (Fig. 4a). In accordance with the previous studies, those proteins which possessed 2 GLYI domains of approximately 120 aa in a single polypeptide, served as the putative Ni^2+^-dependent forms, while those having approximately 142 aa long single GLYI domains and also possessing two extra stretches of sequences compared to other GLYI proteins, served as the putative Zn^2+^-dependent forms. Therefore, domain organisation pattern could also serve as an indicator for the type of metal ion dependency of the GLYI proteins. Based on this criterion, SbGLYI-7, SbGLYI-10, SbGLYI-11 and SbGLYI-14 could be classified as Ni^2+^-dependent and SbGLYI-8 as Zn^2+^-dependent (Table 3). This result is in line with the phylogenetic analysis, with metal binding sites also being conserved in these proteins (Additional file 3: Figure S3 and Table 3). Likewise, domain architecture analysis of GLYII proteins revealed the presence of metallo-β-lactamase domains in all GLYII proteins (Fig. 4b). However, out of the 7 SbGLYII proteins, only 2 proteins namely, SbGLYII-3 and SbGLYII-4, were found to possess HAGH_C (PF01623) domain in addition to the metallo-β-lactamase (PF00753) domain (Fig. 4b). The metal binding site THHHXDH, was found to be conserved in SbGLYII-3 and SbGLYII-4 (Table 4 and Additional file 4: Figure S4). In addition, the active site C/GHT residues were also present in SbGLYII-3 and SbGLYII-4, and even in SbGLYII-5 (Additional file 4: Figure S4). But SbGLYII-5 being similar to OsGLYII-1, was predicted to be a sulphur dioxygenase enzyme. The domain organisation of inactive GLYII proteins was very different from the active GLYII proteins having different additional domains. They were predicted to possess domains such as pre-mRNA 3′-end-processing endonuclease polyadenylation factor C-term, as found in SbGLYII-1 and SbGLYII-2, whereas SbGLYII-6 had *Fer4_13* towards its N-terminus (Fig. 4b).

**Table 3:** Information on domain organisation of SbGLYI proteins for the prediction of enzymatic activity and metal ion dependency

Protein	Protein domain (PF00903)			Presence of conserved metal binding sites^*^				Predicted enzymatic activity	Metal ion dependency Ni^2+^/Zn^2+^
	Start	End	Length	H/Q	E	H/Q	E		
SbGLYI-1.1	5	133	128	+	–	+	–	Absent	–
SbGLYI-1.2	7	124	117	+	–	+	–	Absent	–
SbGLYI-2	9	133	124	–	+	–	–	Absent	–
SbGLYI-3	273	394	129	+	–	–	–	Absent	–
SbGLYI-4	52	174	123	+	–	+	+	Absent	–
SbGLYI-5	21	140	120	+	–	+	+	Absent	–
SbGLYI-6	215	371	157	+	–	–	–	Absent	–
SbGLYI-7	85	205	121	+	+	+	+	Present	Ni^2+^
	216	335	120	+	+	+	+	Present	Ni^2+^
SbGLYI-8.2	72	214	144	+	+	+	+	Present	Zn^2+^
SbGLYI-8.1	57	201	144	–	+	+	+	Present	Zn^2+^
SbGLYI-9	19	142	123	+	–	+	+	Absent	–
SbGLYI-10	25	144	121	+	+	+	–	Present	Ni^2+^
	161	277	124	+	+	+	+	Present	Ni^2+^
SbGLYI-11	29	149	121	+	+	+	+	Present	Ni^2+^
	161	281	124	+	+	+	+	Present	Ni^2+^
SbGLYI-12	70	185	116	+	+	+	+	Absent	–
SbGLYI-13	26	144	119	+	–	+	–	Absent	–
SbGLYI-14	92	212	128	+	+	+	+	Present	Ni^2+^
	224	339	128	+	+	+	+	Present	Ni^2+^
SbGLYI-15	7	121	114	–	+	–	–	Absent	–

* ‘-’ indicates absence of the corresponding residue and ‘+’ indicates its presence

**Table 4:** Information on domain organisation of putative SbGLYII proteins for the prediction of conserved motifs and enzyme activity

Protein	Protein domain						Conserved metal binding motif THHHXDH	Active site motif C/GHT	Expected Enzymatic activity
	PF00753			PF16123					
	Start	End	Length	Start	End	Length			
SbGLYII-1	43	203	205	–	–	–	Absent	Absent	No
SbGLYII-2.1	17	205	197	–	–	–	Absent	Absent	No
SbGLYII-2.2	22	188	193	–	–	–	Absent	Absent	No
SbGLYII-3	175	256	81	14	66	74	Present	Present	Yes
SbGLYII-4	252	336	84	111	251	156	Present	Present	Yes
SbGLYII-5	66	235	172	–	–	–	Absent	Present	No
SbGLYII-6	148	307	162	–	–	–	Absent	Absent	No

### Developmental variations and stress-mediated expression profiling of sorghum glyoxalase genes

**Fig. 5:**
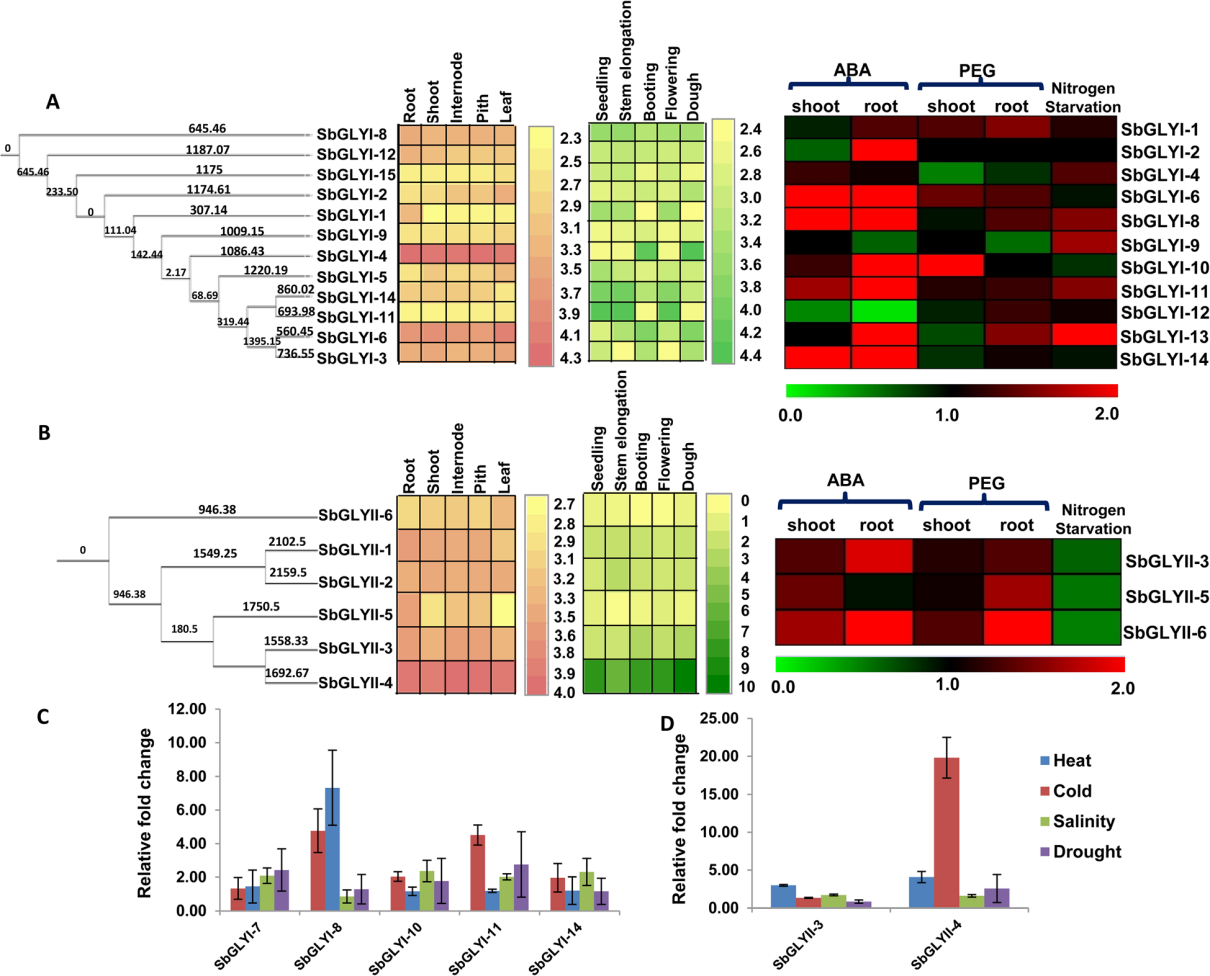
Developmental and stress-mediated regulation of glyoxalase family genes from sorghum. Expression profile of (**a**) *GLYI* and (**b**) *GLYII* genes was obtained from the publicly available Genevestigator Affymetrix sorghum genome array database. Normalized transcript data was obtained for different tissues, viz. underground tissues - root and, aerial tissues - leaf, internode, shoot and pith (left panel) at different developmental stages (middle panel). Normalized and curated perturbation expression data (right panel) of the genes was retrieved from Expression Atlas. Fold change in expression pertaining to ABA treatment (20 μM), PEG treatment (20% PEG 8000) and nutrient nitrogen limitation has been shown as heatmap generated using MeV software package. Colour scale below or on the right of the heatmap shows the level of expression. *GLY* genes have been represented in order as per their phylogenetic relationship. Branch length represents evolutionary time between the two nodes. Histogram depicting relative expression levels of (**c**) *SbGLYI* and (**d**) *SbGLYII* genes under different abiotic stress treatments viz. heat, cold, salinity (given to 7 d old seedlings for 6 h) and drought (water withheld for 48 h). Expression levels have been calculated with respect to the untreated control (having value of 1)

In order to study the anatomical and developmental regulation of glyoxalase genes in sorghum, gene expression profile of putative *SbGLYI* and *SbGLYII* genes was retrieved from the Genevestigator database. Expression data, however, could not be obtained for *SbGLYI-3*, *SbGLYI-5*, *SbGLYI-7* and *SbGLYI-13* genes. Expression analyses revealed that, of all the *GLYI* genes, the expression of *SbGLYI-4* did not show tissue-specific variations and was constitutively expressed at higher levels in all the tissues (Fig. 5a, left panel). However, developmental stage mediated variations existed in the expression of *SbGLYI-4*, with its transcript levels being higher at the booting and dough stage of development (Fig. 5a, middle panel). Further, another GLYI-like gene, *SbGLYI-6,* showed relatively higher expression in leaves and even exhibited gradual increase in transcript abundance during different stages of development. However, putative Ni^2+ −^dependent forms, *SbGLYI-11* and *SbGLYI-14,* were found to maintain higher levels of expression from the seedling stage till the flowering stage which thereafter, declined (Fig. 5a, middle panel). The expression of putative Zn^2+^ − dependent *SbGLYI-8,* was however, found to be similar in all the tissues and even at different developmental stages (Fig. 5a, middle panel). Among *GLYII* genes, *SbGLYII-4* showed highest expression which was maintained across all the tissues (Fig. 5b, left panel). Developmental variations could be seen in its expression, being lowest at the stem elongation stage and highest during the dough stage, but still more than the other *SbGLYII* genes (Fig. 5b, middle panel).

Further, normalised and curated perturbation expression data was retrieved from the publicly available database; Expression Atlas, with an aim to study the stress-mediated regulation of glyoxalase genes in sorghum. It was found that the expression of *SbGLYI-6, SbGLYI-8, SbGLYI-11* and *SbGLYI-14* was up-regulated in response to ABA treatment in both roots and shoots while *SbGLYI-2* and *SbGLYI-13* genes were induced only in roots upon ABA treatment (Fig. 5a, right panel). In response to PEG treatment, *SbGLYI-1, SbGLYI-6* and *SbGLYI-10* seemed to play a significant role as their expression levels were higher in comparison to the other *GLYI* genes. *SbGLYI-2* and *SbGLYI-13* were however, highly down regulated in response to PEG treatment. All the *SbGLYI* genes, except *SbGLYI-6, SbGLYI-10* and *SbGLYI-14* showed an induction in response to nitrogen starvation conditions in either root or shoot tissues (Fig. 5a, right panel).

Further, analysis of *SbGLYII* expression revealed that *SbGLYII-3*, *SbGLYII-5* and *SbGLYII-6* genes showed a similar pattern of expression in response to ABA treatment in shoot, osmotic stress in root, and nitrogen stress (Fig. 5b, right panel). However, expression data was not available for *SbGLYII-1*, *SbGLYII-2* and *SbGLYII-4*. Taken together, the genes were found to be induced in response to abiotic stresses but were down-regulated in response to nutrient stress (Fig. 5b, right panel). Notably, expression of *SbGLYII-5,* which encodes SDO activity, was different from the other two proteins and was found to be unaltered in roots in response to ABA treatment and in shoots in response to osmotic stress.

Further, as glyoxalases have a well-established role in plant stress response, we also determined stress-mediated alterations in the expression levels of sorghum glyoxalase genes through qRT-PCR. Expression profiling of putative enzymatically active SbGLY forms was carried out under different abiotic stress conditions viz. heat, cold, salinity and drought (Fig. 5c & d). Interestingly, Ni^2+^-dependent *SbGLYI* genes namely, *SbGLYI-7*, *SbGLYI-10, SbGLYI-11* and *SbGLYI-14,* were found to be induced in response to most of the stress treatments (Fig. 5c). The expression of putative Zn^2+^-dependent *SbGLYI-8* was however, 4.7-fold and 7.3-fold increased under heat and cold stress, respectively but marginally declined under salinity conditions. Similarly, functionally active *SbGLYII* genes also showed stress-mediated perturbations in the expression levels. *SbGLYII-3* expression was found to be ~ 3-fold up-regulated under heat stress whereas *SbGLYII-4* expression was found to be 20-fold higher under cold stress (Fig. 5d).

### Identification and analysis of genes encoding D-lactate dehydrogenase enzymes in sorghum

D-lactate dehydrogenases (D-LDH) have been found to be involved in the metabolism of MG catalysing the conversion of D-lactate to pyruvate, the last step of the pathway. However, no genome-wide study has ever been carried out, in particular for any plant D-LDH. Hence, in addition to the *GLYI* and *GLYII* genes, we also searched for the *D-LDH* genes in the sorghum genome. In order to identify the D-LDH encoding genes in sorghum, HMM profile of FAD_binding_4 (PF01565) was searched against the sorghum database because D-LDH belongs to the FAD binding super-family of proteins. Initial screening led to the identification of 43 genes having FAD _binding _4 domains (Additional file 5: Table S1). The proteins encoded by these genes share a conserved FAD binding domain, but may have different catalytic activities. Thus, it was important to identify genes specifically encoding for D-LDH activity. For this, multiple sequence alignment and phylogenetic analyses was performed for the 43 sequences which revealed 5 major clusters (Additional file 6: Figure S5 and Additional file 7: Figure S6). Proteins in these different clusters had additional domains specific to each cluster except for cluster II which did not possess a second domain (Additional file 7: Figure S6). Presence of different second domains in these proteins could be correlated to different catalytic functions. One of the clusters comprising of 5 proteins (Cluster III) contained previously characterised D-LDH from *Arabidopsis* and rice (Additional file 6: Figure S5 and Additional file 7: Figure S6). Further, the Cluster II proteins having no additional second domains were not predicted to possess any specific catalytic functions. Keeping in view, the features of Cluster II and III, we suggest that proteins in these clusters could possibly code for D-LDH proteins. Therefore, four genes from sorghum were ultimately predicted to code for proteins with D-LDH activity (Table 5). These putative D-LDH proteins had iso-electric point (pI) ranging from 6 to 8 and were predicted to be localised in mitochondria or cytoplasm.

**Table 5:** List of probable D-LDH genes present in *Sorghum bicolor*

Gene name	Locus Name	Transcripts	Coordinate (5′-3′)	Transcript length (bp)	CDS (bp)	Protein			Localisation
						Length (aa)	MW (kDa)	pI	
*SbDLDH-1*	Sobic.002G042400	Sobic.002G042400.1	4,037,195..4048696	2045	1791	596	64.78	6.24	Mitochondria
*SbDLDH-2*	Sobic.002G058500	Sobic.002G058500.1	5,652,091..5665483	2363	1686	561	61.17	6.58	Mitochondria
		Sobic.002G058500.2	5,652,091..5665483	2360	1686	561	61.17	6.58	Mitochondria
*SbDLDH-3*	Sobic.004G355600	Sobic.004G355600.1	68,289,531..68293955	2232	1362	453	52.35	6.9	Cytoplasm
		Sobic.004G355600.2	68,289,531..68293955	2075	1362	453	52.35	6.9	Cytoplasm
*SbDLDH-4*	Sobic.010G277300	Sobic.010G277300.1	61,012,418..61019694	2197	1734	577	66.9	8.53	Cytoplasm
		Sobic.010G277300.2	61,012,418..61019694	2107	1686	561	65.01	8.41	Cytoplasm

### Gene structure, domain organisation and phylogenetic analyses of sorghum D-LDH proteins

**Fig. 6:**
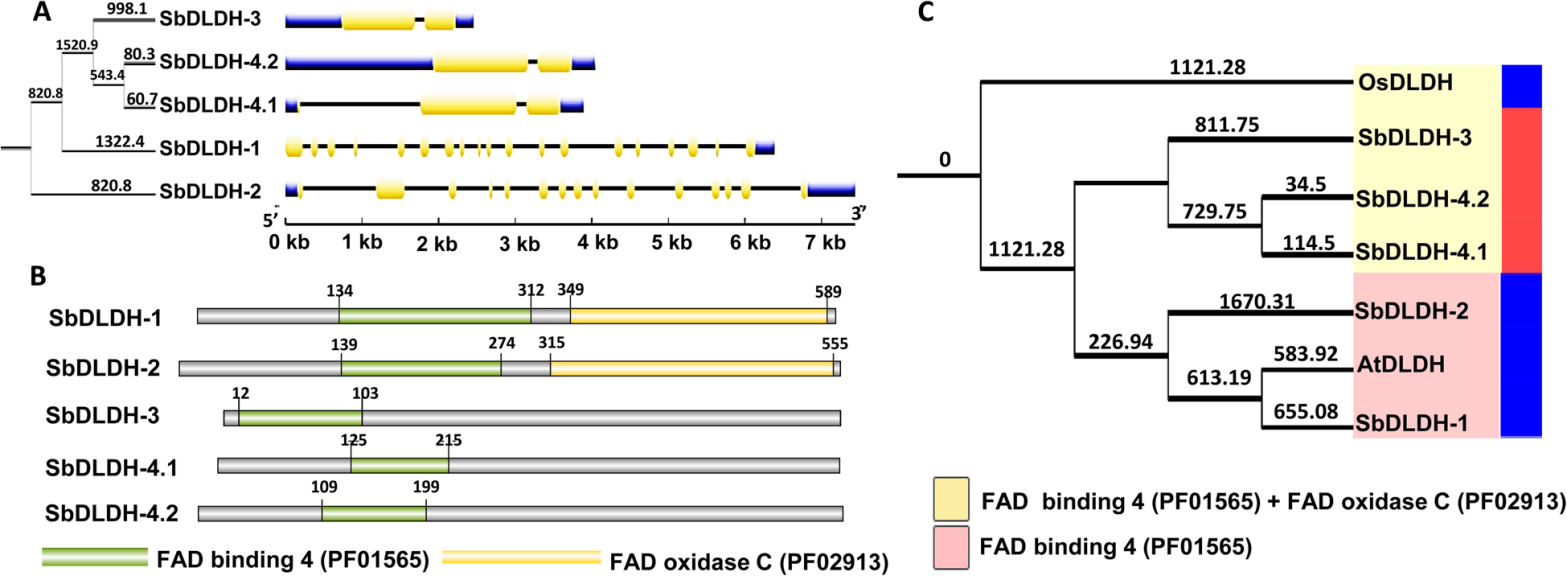
Elucidation of exon-intron structure, protein domain architecture and phylogenetic relationship between sorghum D-LDH proteins. **a** Exon-Intron structure of *SbDLDH* genes. Length of exons and introns have been represented proportionally as indicated by the scale at the bottom. **b** Schematic representation of domain architecture of SbDLDH proteins indicating the presence of FAD_binding_4 and FAD_oxidase_C domains in SbDLDH proteins **c** Full length amino acid sequence of SbDLDH proteins were compared with the known D-LDH proteins from rice and *Arabidopsis* and phylogenetic tree was constructed using the Neighbour-Joining method in MEGA 7.0 with 1000 bootstrap replicates. Putative sub-cellular localisation of proteins have been indicated towards the right of the tree in different colours; cytoplasm (red) and mitochondria (blue)

*SbDLDH* genes did not show characteristically similar exon-intron patterns as found for *SbGLYI* genes (Fig. 6a). *SbDLDH-1* had the highest number of exons followed by *SbDLDH-2*. Both these proteins consisted of FAD _oxidase _C domain in addition to the FAD_binding_4 domain (Fig. 6b and Table 6). SbDLDH-3, SbDLDH-4.1 and SbDLDH-4.2 proteins consisted of only FAD_binding_4 domains. Further, phylogenetic analyses indicated SbDLDH-1 and SbDLDH-2 to cluster with AtDLDH, and hence, were predicted to be functionally similar (Fig. 6c). Likewise, both SbDLDH-1 and SbDLDH-2 were predicted to be mitochondrial proteins similar to their *Arabidopsis* AtDLDH ortholog (Fig. 6c). SbDLDH-3, SbDLDH-4.1 and SbDLDH4.2 proteins shared greater sequence similarity to rice OsDLDH, and likewise possessed both the domains. However, unlike OsDLDH which is a mitochondrial protein, SbDLDH-3, SbDLDH-4.1 and SbDLDH-4.2 were predicted to be cytoplasmic proteins (Table 5).

**Table 6:** Domain architecture analysis of SbDLDH proteins from *Sorghum bicolor*

Protein	Domain name	Domain coordinates		
		Start	End	Length
SbDLDH-1	FAD_binding_4	134	312	179
	FAD_oxidase_C	349	589	241
SbDLDH-2	FAD_binding_4	139	274	136
	FAD_oxidase_C	315	555	240
SbDLDH-3	FAD_binding_4	12	103	92
SbDLDH-4.1	FAD_binding_4	125	215	91
SbDLDH-4.2	FAD_binding_4	109	199	91

### Developmental stage-specific and stress-mediated variations in the expression profile of D-LDH genes of sorghum

**Fig. 7:**
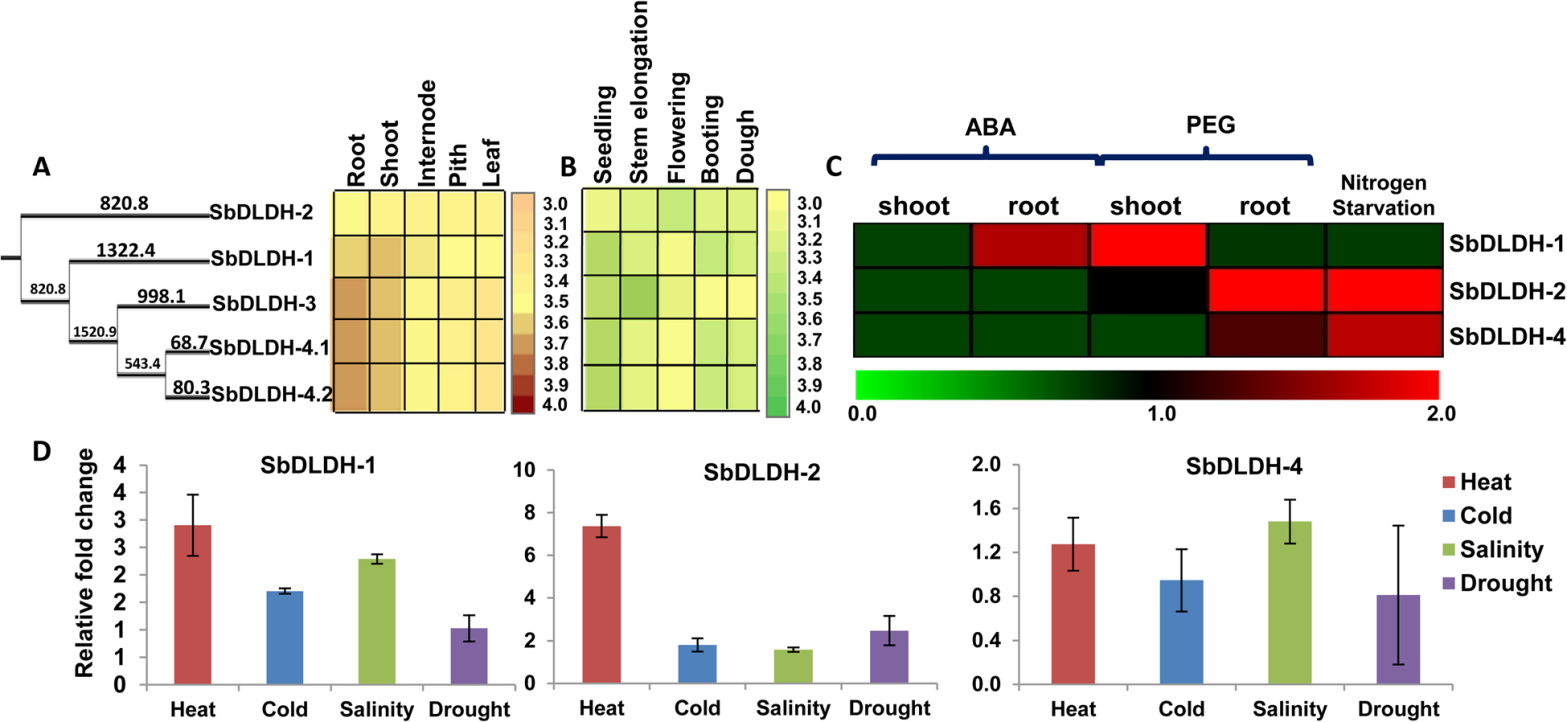
Developmental and stress-mediated regulation of D-LDH genes from sorghum. Genome-wide microarray data of *D-LDH* genes was obtained from the publicly available Genevestigator Affymetrix sorghum genome array database. Normalized transcript data was obtained for (**a**) different tissues, including underground tissues-root and, aerial tissues- shoot, leaf, internode and pith and at (**b**) different developmental stages. **c** Normalized and curated perturbation expression data of the sorghum *D-LDH* genes was retrieved from Expression Atlas. Fold change in expression pertaining to ABA treatment (20 μM), PEG treatment (20% PEG 8000) and nutrient nitrogen limitation has been shown as heatmap generated using MeV software package. Colour scale below and on the right of the heatmap shows the levels of expression. Genes have been represented in order as per their phylogenetic relationship. Branch length represents evolutionary time between the two nodes. **d** Histogram depicting relative expression levels of *SbDLDH* genes under different abiotic stress treatments viz. heat, cold, salinity, (given to 7 d old seedlings for 6 h) and drought (water withheld for 48 h). Expression levels have been calculated with respect to the untreated control (having a value of 1)

Similar to glyoxalases, development and tissue-specific variations in expression were determined for *SbDLDH* genes as well. Of the four sorghum D-LDH genes, *SbDLDH-1* was found to be expressed at greater levels in shoots than roots (Fig. 7a) whereas expression of *SbDLDH-3* and *SbDLDH-4* was greater in roots than in shoots (Fig. 7a). *SbDLDH-2* selectively showed lower expression in all the tissues and across different developmental stages except for the flowering stage (Fig. 7b). All the other *SbDLDH* genes showed stronger expression at the seedling stage. However, *SbDLDH-3* had higher expression even at the stem elongation stage.

To understand the regulation of D-LDH proteins in response to stress, transcript levels of *SbDLDH* genes were analysed under osmotic (PEG) and drought (ABA) stress conditions (Fig. 7b). Data could not be obtained for *SbDLDH-3* and hence was not included in Fig. 7c. All the analysed *SbDLDH* genes were found to be down-regulated in response to ABA treatment in both roots and shoots except for *SbDLDH-1* which was induced upon ABA treatment in roots. PEG treatment also led to an increase in *SbDLDH-1* expression in shoots whereas *SbDLDH-2* transcript levels increased in roots upon PEG treatment. Further, *SbDLDH-2* and *SbDLDH-4* but not *SbDLDH-1* showed an increase in expression levels in response to nitrogen starvation (Fig. 7c). Further, a qRT-PCR-based expression profiling of putative functionally active *SbDLDH* isoforms under stress conditions revealed increased expression of these genes under heat, cold, salinity and drought conditions. The alteration in *SbDLDH-2* expression was however, insignificant under cold and salinity stress as compared to the other two genes under the same conditions (Fig. 7d). Moreover, we could not determine stress-mediated variations in the S*bDLDH-3* expression as its transcript remained undetected under stress conditions.

### Three-dimensional homology modelling of SbDLDH proteins

**Fig. 8:**
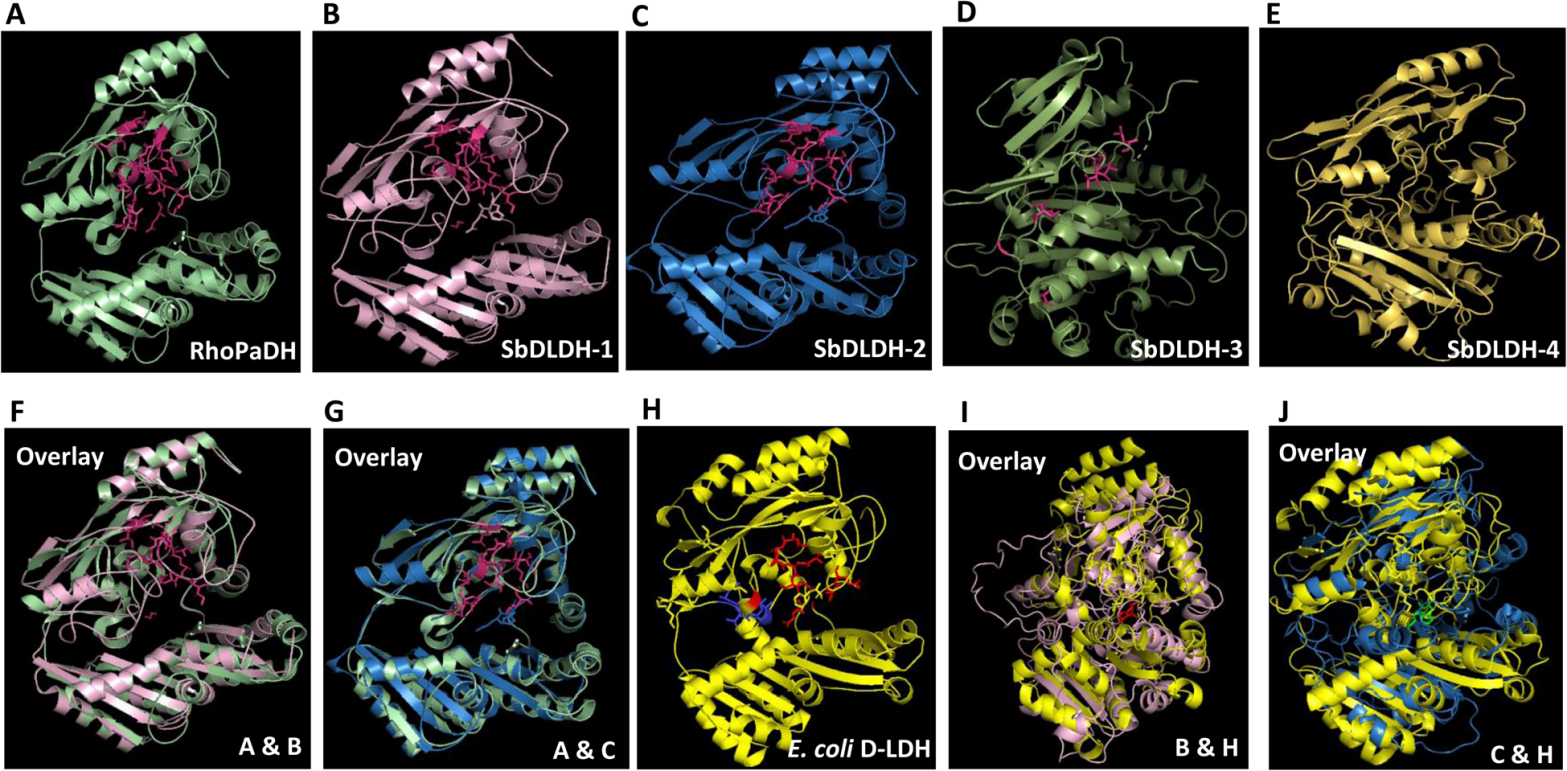
Three dimensional structure of putative D-LDH proteins from sorghum generated through homology modelling. Three dimensional structures of putative D-LDH proteins were modelled using *Rhodopseudomonas palostris (RhoPaDH)* putative dehydrogenase (RhoPADH) (**a**) as template. Structures of (**b**) SbDLDH-1, (**c**) SbDLDH-2, (**d**) SbDLDH-3 and (**e**) SbDLDH-4 showing conserved FAD binding sites (marked in pink). SbDLDH-1 and SbDLDH-2, closest in structural similarity to RhoPaDH, are shown as overlay with RhoPaDH (**f**&**g**). Three-dimensional structure of D-LDH from *E. coli* (**h**) has also been shown as overlay with SbDLDH-1 (**i**) and SbDLDH-2 (**j**). Red indicates FAD binding site in *E. coli*, blue indicates catalytic site of *E. coli* D-LDH

Since no three-dimensional protein structures are yet available for any plant D-LDH proteins, a three-dimensional homology modelling study of SbDLDH proteins was attempted using information from the other systems. For structure prediction, the putative SbDLDH proteins were searched against the Protein Data Bank in the NCBI Blast server. A putative dehydrogenase from *Rhodopseudomonas palostris* (RhoPaDH, 3PM9_A) was found to be the closest available structural ortholog of the SbDLDH proteins. Once the structure of RhopaDH (Fig. 8a) was obtained from the Protein Data bank, the structure of the SbDLDH proteins (Fig. 8b-e) were modelled using the RhoPaDH structure as a template. Upon structural alignment and superposition onto the RhoPaDH protein, FAD-binding domain residues were found to be conserved in SbDLDH-1 and SbDLDH-2 (Fig. 8f, g) but lacking in SbDLDH-3 and SbDLDH-4 (data not shown). Further, structures of D-LDH proteins from sorghum were also modelled using *Escherichia coli* (*E. coli*) D-LDH (1F0X) as a template. Reports on the crystal structure of *E.coli* D-LDH (Fig. 8h) suggest that the flavin ring of FAD specifically interacts with the residues, Leu 81, Ile-147, Phe-39, Ser-144, Glu-528 and His-529 [29]. Although the position of the active site is not known, it is suggested that its location is close to the iso-alloxazine ring of FAD in the neighbourhood of Ile-142 and Ser-144, and is a part of the FAD-binding domain [29]. Upon structural alignment of SbDLDH proteins with the *E.coli* D-LDH protein, we found Glu-528 and His-529 residues to be conserved in SbDLDH-1 and SbDLDH-2 (Fig. 8i, j). It was however, observed that SbDLDH proteins were more similar to RhoPaDH than to the *E.coli* D-LDH.

## Discussion

Agricultural productivity is adversely affected by various environmental factors resulting in extensive yield losses worldwide. Plants growing in the field generally face a combination of different stresses at any given time, severely limiting their growth and productivity [30]. But owing to their inherent plastic nature, plants have acquired a remarkable ability to adapt to variable climatic conditions and multiple stresses via the evolution of diverse mechanism for stress alleviation. Of these, some pathways are important not only for stress adaptation but also serve crucial housekeeping functions. The glyoxalase system, which is involved in the degradation of MG, can be termed as an apt example of one such mechanism. MG is a potent glycating agent that can readily modify proteins, lipids and nucleic acids [2] causing large-scale modifications in the plant cellular components and thus, its levels need to be tightly controlled [31, 32]. Since generation of MG in the living systems is inevitable, glyoxalases which detoxify MG are ubiquitously found in all organisms. Increase in MG levels during stress is in fact, a universal phenomenon in plants with a number of reports reiterating the role of MG and glyoxalases during stress conditions [33, 34]. Therefore, it would not be inappropriate to term MG and glyoxalases as possible biomarkers of plant stress tolerance [15]. To this end, over-expression of glyoxalase pathway genes has been carried out in various plant species wherein through improved MG detoxification as a result of increased activity of glyoxalase pathway enzymes, MG levels could be restricted from rising under stress, thereby imparting enhanced stress tolerance to plants [33, 35–38].

Previous genome-wide studies carried out in *Arabidopsis*, *Oryza sativa* [18], *Glycine max* [19], *Medicago truncatula* [20] and *Brassica rapa* [21] have identified the presence of glyoxalase pathway genes as multiple members in these plant species and shown them to be differentially regulated in response to various abiotic stresses. However, no previous studies, ever reported the status of D-LDH proteins in plants. Experimental evidence suggests a crucial role of NADH-independent D-LDH proteins in the MG detoxification pathway which constitutes the last step of this process [12–14]. Accumulation of D-lactate produced by the reactions of the glyoxalase pathway enzymes, can otherwise confer toxicity in the form of lactic acidosis, not being able to be utilized in any other metabolic pathway [39]. Therefore, with an aim to investigate the relevance of MG detoxification in sorghum, one of the top five most versatile and economically important cereal crops [26], we have undertaken a genome-wide distribution and expression profiling analysis of genes involved in the MG detoxification pathway.

A comprehensive genome-wide distribution study led to the identification of 15 *GLYI*, 6 *GLYII* and 4 *D-LDH* genes in the sorghum genome. Like in other plant species, SbGLYI proteins could also be broadly classified into two major categories. First category comprised of functionally active GLYI proteins, which based on their metal activation properties could be further classified into Zn^2+^- and Ni^2+^- dependent proteins. The metal specificity of SbGLYI proteins was predicted based on their domain sequence and length [40, 41]. Four SbGLYI proteins namely, SbGLYI-7, SbGLYI-10, SbGLYI-11 and SbGLYI-14, were found to be Ni^2+^-dependent showing greater homology to the previously characterised Ni^2+^-dependent GLYI proteins from rice and *Arabidopsis* [42, 43] and having a similar domain length, of approximately 120 aa. Similarly, only one GLYI protein namely, SbGLYI-8 was found to be Zn^2+^-dependent, having a domain length of 140 aa, much like rice OsGLYI-8 [44] and *Arabidopsis* AtGLYI-2 [43, 45] proteins. Interestingly, *SbGLYI-8* possessed two spliced forms coding for almost similar length proteins (214 and 227 aa long), and both were predicted to be similarly localised in the mitochondria and/or chloroplast. This was unlike *AtGLYI-2* from *Arabidopsis*, where three out of the four spliced forms coded for the same protein (AtGLYI-2.1/2/3, 187 aa) and only one was different (AtGLYI-2.4) being 236 aa long [45]. The longer AtGLYI-2.4 protein form was more similar to rice OsGLYI-8, both in length as well as in nuclear localization [44]. The AtGLYI-2.4 protein, however, also localizes to the chloroplast as reported by Schmitz et al. [45]. Likewise, SbGLYI-8/8.1 proteins were also found to harbour putative nuclear localization signals (NLS) and therefore, may get localized in the nucleus as well.

The other category of proteins comprised of functionally diverse and possibly inactive GLYI-like proteins. Schmitz et al. [27] have recently proposed the occurrence of functional divergence in the *Arabidopsis* glyoxalase family. In *Arabidopsis*, eight proteins were reported to be the members of the GLYI-like category of proteins which lacked conserved motifs and shared only 17–21% sequence identity to AtGLYI-2, the Zn^2+^-dependent form. Their biological activity is yet to be elucidated and even no close bacterial homologs have been identified to date. Importantly, Schmitz et al. [27] also pointed that the phylogenetic occurrence of GLYI-like proteins is restricted to bacteria and the green lineage.

Among the SbGLYII proteins, SbGLYII-3 and SbGLYII-4 were predicted to be active GLYII enzymes owing to the presence of conserved metal binding motifs and their high sequence similarity to the respective functionally active OsGLYII-2 [46] and OsGLYII-3 proteins from rice. SbGLYII-5, however, lacked the conserved THHHXDH metal binding motif and instead showed high sequence similarity to SDO activity-encoding OsGLYII-1 [47] and AtGLY2–3 proteins [48]. Hence, *SbGLYII-5* was predicted as a putative SDO enzyme. It is now clear that, like GLYI family, functional divergence has also occurred in the GLYII family and this is seen in all plant species studied so far. GLYII proteins belong to the super-family of metallo-β-lactamase proteins which include proteins of different functions such as arylsulfatase, cyclase/dihydrase, lactams, phosphonate derivatives etc. [49]. Previously, distinction between different members of this super-family was not clear with all proteins possessing the metallo-β-lactamase fold being annotated as putative GLYII proteins, as also was done for rice [18]. However, with the sequence and crystal structure analysis of true GLYII proteins, a C-terminus located HAGH_C domain has been identified in the functionally active GLYII enzymes and it is suggested that substrate binding occurs at the interface between this domain and the catalytic β-lactamase domain [50]. Therefore, the presence of HAGH_C domain provides more confidence in the prediction of β-lactamase fold-containing protein as a true GLYII enzyme and our results are in accordance with it.

**Fig. 9:**
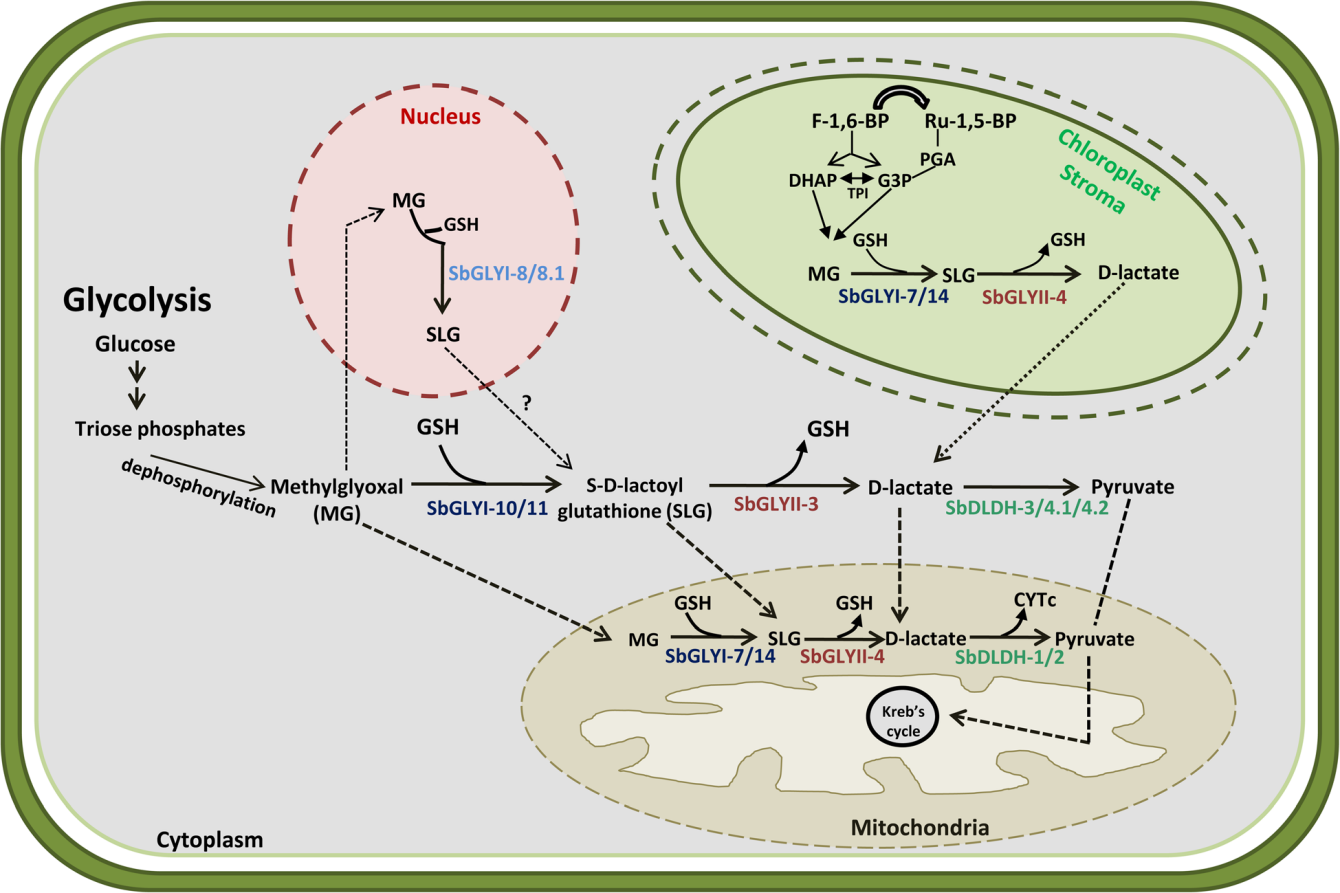
Proposed model of methylglyoxal detoxification via glyoxalase pathway proteins in various subcellular organelles of sorghum. Cellular defence against MG probably involves four different sub-cellular compartments viz. cytosol, chloroplast, mitochondria and nucleus. Cytosolic MG produced as an off shoot of glycolysis is converted to SLG by SbGLYI-10/11 which is further converted to D-lactate by SbGLYII-3. The conversion of D-lactate to pyruvate is catalysed either by SbDLDH-3, 4.1 or 4.2. Both in the mitochondria as well as chloroplast, MG detoxification is predicted to be catalysed by the same SbGLYI and SbGLYII proteins. D-lactate produced in the chloroplast can be converted to pyruvate either by cytosolic SbDLDH protein or transported in the mitochondria. In mitochondria, D-lactate is converted to pyruvate by SbDLDH-1/2 protein. Pyruvate is then fed into the Kreb’s cycle. In the nucleus, SbGLYI-8/SbGLYI-8.1, may catalyse the conversion of MG to SLG. Nuclear export of SLG is proposed as no nuclear GLYII could be predicted in the sorghum genome. TPI-Triose phosphate isomerase, GSH-Glutathione, G3P-Glyceraldehyde-3-Phosphate, F-1,-6-BP- Fructose-1,6-bisphosphate, Ru-1,5-BP- Ribulose-1,5, bisphosphate, PGA- Phosphoglyceraldehyde

The last step of MG detoxification is catalysed by the D-LDH enzyme. These proteins belong to the FAD_binding_4 superfamily which use FAD as a cofactor. There are 43 such proteins in sorghum. In addition to the presence of FAD_binding_4 domain, most of these proteins contain an additional second domain which may be used to identify the catalytic functions of these proteins. In the case of D-LDHs, we found that out of the four possible D-LDHs identified on the basis of their sequence similarity to the previously characterized rice and *Arabidopsis* D-LDH proteins, two of them did not possess second domain while the other two had a FAD_oxidase_C domain. The remaining 39 proteins had different second domains such as, ALO (D-arabino-1,4-lactone oxidase), BBE (berberine and berberine like) and Cytokinin binding domain and are known to be involved in the D-erythroascorbic acid biosynthesis pathway [51], in biosynthesis of numerous isoquinoline alkaloids [52], and are present in plant cytokinin dehydrogenase, respectively [53]. SbDLDH proteins were predicted to be localized in either mitochondria or cytoplasm. Mitochondria is one of the potential sites for MG production and detoxification, possibly favouring the cell in protection against oxidative damage. The predicted presence of SbDLDH proteins in the mitochondria is in fact, in agreement with the known mitochondrial localization of D-LDH proteins from rice and *Arabidopsis* [12–14]. Further, it is possible that these mitochondrial D-LDH enzymes might acquire their substrate from within the organelle as few functionally active GLYI (SbGLYI-7 and SbGLYI-14) and GLYII (SbGLYII-4) proteins were also predicted to be present within the mitochondria (Fig. 9). Even otherwise, cytoplasm-generated D-lactate is also known to translocate to mitochondria for its metabolism to pyruvate by the mitochondrial D-LDH proteins [54]. Nonetheless, even cytoplasmic D-LDH proteins were predicted in the sorghum genome and included SbDLDH-3 and SbDLDH-4 proteins (Fig. 9).

Transcript abundance analysis of putatively active *SbGLY* genes in different tissues and at different developmental stages revealed constitutive expression of *SbGLYI-8*, *SbGLYI-11, SbGLYI-14, SbGLYII-3* and *SbGLYII-4*, similar to the observed constitutive expression pattern of active glyoxalases in rice (i.e *OsGLYI-2, OsGLYI-8, OsGLYI-11.2, OsGLYII-2* and *OsGLYII-3*) and *Arabidopsis* (*AtGLYI-2, AtGLYII-2* and *AtGLYII-5*) at all developmental stages and in all the tissues [18]. Schmitz et al. [27] have indeed suggested that functional glyoxalase enzymes are constitutively expressed probably for maintaining MG levels below toxic limits. It is well known that alterations in the expression of genes at the transcriptional level is one aspect of stress response. Glyoxalases from different plant species such as, *AtGLYI-4, AtGLYI-7* (*Arabidopsis*), *OsGLYI-6, OsGLYI-11* (rice), *GmGLYI-6, GmGLYI-9, GmGLYI-20, GmGLYII-5, GmGLYII-10* (soybean), *MtGLYI-8, MtGLYI-21, MtGLYII-9* (*M. truncatula*) and *BrGLYI-3* (*B. rapa*) were previously reported to show high expression as a response to abiotic stress [18–21]. Likewise in the present study, we observed most of the members of sorghum glyoxalase family to be highly stress-responsive. For instance, the rice *OsGLYI-8* ortholog *SbGLYI-8*, is significantly induced under both heat and cold stress and a GLYII-encoding *SbGLYII-4* gene is induced under cold stress. Further, *SbGLYI-8*, *SbGLYI-11*, *SbGLYI-14*, *SbGLYII-3* and *SbGLYII-6* were also up-regulated in response to exogenous ABA treatment and osmotic stress. Previous studies show upregulation of *SbGLYI-11* even in response to combined heat and drought stress [55], and is similar to the results obtained in the current study demonstrating increased *SbGLYI-11* expression under both, heat and drought stress. Moreover, a *SbGLYII* gene isolated via RT-PCR from the *Egyptian Sorghum* cv. R3 by Assem et al. [56] has been identified as one of the two salt-tolerant alleles reported in the study. Further, the fungicide Maneb is also known to induce GLYI activity in sorghum indicating a pro-active antioxidant machinery operating in plants under such conditions [57]. However, among the D-LDH genes, only *SbDLDH-1*, encoding a putative mitochondrial protein, was found to be induced in response to ABA and PEG treatment.

Besides exogenous ABA and osmotic stress responsiveness, most of the GLYI genes are also induced in response to nitrogen (N) starvation. In the case of D-LDH, the significantly higher transcript abundance of *SbDLDH* genes under abiotic stresses viz. heat, cold, salinity and drought indicates their role in abiotic stress response in sorghum. In addition, *SbDLDH-2* and *SbDLDH-4* genes were also induced in response to nitrogen limitation suggestive of their role in MG detoxification during N stress in sorghum. In fact, a comparative study conducted in the two sorghum genotypes viz. 3P4 and 4P11, has revealed an increase in GLYI and GLYII activities in the plants subjected to both N-deficient and N-excess conditions, especially in the case, where ammonium was used as a N source [58]. In a similar context, the impact of MG in contributing towards NH_4_^+^ toxicity symptoms in *Arabidopsis* has been recently studied [59]. Since effective incorporation of ammonium ions into the amino acid structures entails high activity of mitochondrial TCA and engagement of the glycolytic pathway, generation of MG is unavoidable under such circumstances. In fact, MG generation was shown to supersede the repair capacity of detoxification enzymes leading to toxicity symptoms in plants. Hence, it can be safely said that a correlation exists between MG detoxification and N metabolism in plants.

Having identified the putatively active SbGLY and SbDLDH proteins in different sub-cellular compartments, we propose a cellular model for MG detoxification via the glyoxalase pathway in sorghum (Fig. 9). Our in silico analysis indicates that cellular defence against MG involves at least four different sub-cellular compartments viz. cytosol, chloroplast, mitochondria and nucleus. The cytosolic MG is converted to SLG by SbGLYI-10 and/or SbGLYI-11 which is then further converted to D-lactate by SbGLYII-3. Interestingly, we found two SbDLDH proteins to be localised in the cytosol which can convert D-lactate to pyruvate, which is then transported to mitochondria by the transport proteins like pyruvate translocase. In the chloroplast, MG produced as a result of degradation of triose sugars derived from Calvin-Benson cycle, may be converted to SLG by SbGLYI-7 and/or SbGLYI-14. The conversion of SLG to D-lactate can be catalysed by SbGLYII-4. The putative chloroplastic glyoxalase proteins which are, predicted to possess dual localization are likely to be present in the mitochondria as well. Therefore, MG in the mitochondria is probably detoxified by the same SbGLYI and SbGLYII proteins. The D-lactate so produced can thus, be converted to pyruvate by SbDLDH-1 and/or SbDLDH-2 proteins in the mitochondria. This pyruvate is ultimately fed into the Kreb’s cycle. Further, MG being a small metabolite can also enter the cell nucleus and exert its harmful effects [44, 60]. To counteract the deleterious effects of MG in the nucleus, SbGLYI-8/SbGLYI-8.1 proteins which possess NLS sequences like their rice and *Arabidopsis* orthologs, may catalyse the conversion of nuclear MG to SLG. However, as no nuclear GLYII could be predicted in the sorghum genome, we propose the nuclear export of SLG to cytosol for its detoxification. However, this model needs to be experimentally validated in order to confirm the role of multiple organelles in the detoxification of MG in the plant cell.

## Conclusion

Unlike the previous reports, the current study has identified the presence of multiple D-LDH genes in sorghum, together with glyoxalase pathway genes, which are needed for complete metabolism of MG into a non-toxic compound, pyruvate. We believe that this study on MG detoxification genes, especially on glyoxalases which are well-established to play important roles in abiotic and biotic stress tolerance, will pave way for future studies aimed at understanding the abiotic stress tolerance mechanisms in sorghum and ultimately pave way for effective abiotic stress alleviation in plants through molecular biology interventions.

## Material and methods

### Identification and nomenclature of glyoxalases and D-LDH genes/proteins in sorghum

To identify all the putative GLYI, GLYII and D-LDH proteins, HMM profile of the conserved glyoxalase (PF00903 and PF12681), metallo-beta-lactamase (PF00753), hydroxyacylglutathione hydrolase (PF16123) and FAD binding_4 (PF01565) domains obtained from the Pfam 32.0 database [61], was searched against the annotated proteins of sorghum using the PhytoMine tool [62] of the Phytozome genome database. For nomenclature, prefix ‘Sb’ was added to GLYI, GLYII and D-LDH followed by Arabic numbers in the increasing order of chromosome number. Alternate splice forms were chronologically numbered. Transcripts of putative functionally active GLY genes were validated using PCR employing primers listed in the Additional file 8: Table S2. The various physical parameters of the protein such as length, molecular weight and theoretical pI were predicted using the ProtParam tool [63]. Sub-cellular localisation of each of the proteins was predicted using the Localiser sub-cellular prediction tool [64], and if not found, WoLF PSORT prediction tool [65] was used. Chloroplast localisation of proteins was confirmed using the ChloroP server [66].

### Evaluation of protein domain architecture

Detailed domain analysis of predicted GLYI, GLYII and D-LDH proteins was carried out using the HMMER Web version 2.31.0 [67]. Domain architecture was represented using the Domain Graph visualisation tool [68].

### Phylogenetic analysis of glyoxalase and D-LDH proteins

For establishing evolutionary relationships, full length or domain amino acid sequence of the predicted sorghum proteins were aligned with the known GLYI, GLYII and D-LDH proteins from different plant species using Clustal in Jalview [69]. Phylogenetic tree was constructed using the Neighbour-Joining method in MEGA 7.0 with 1000 bootstrap replicates [70]. Tree was visualised using the iTOL software [71].

### Developmental and stress-mediated expression profiling of glyoxalase and D-LDH genes in sorghum

The anatomical and developmental microarray data of *SbGLYI*, *SbGLYII* and *SbDLDH* genes was retrieved from the publicly available Genevestigator Affymetrix sorghum genome array database [72]. The normalised and curated perturbation expression data (RNA seq) of the genes was obtained from the Expression Atlas repository from the experiments, E-GEOD-30249 [73] and E-GEOD-54705 [74], corresponding to ABA and PEG, and nitrogen tolerance conditions, respectively. The data was then used to generate heatmap using the Institute for Genomic Research MeV software package [75].

### Three dimensional homology modelling of SbDLDH proteins

For homology modelling, the full length amino acid sequence of the putative SbDLDH proteins were searched against protein data bank in the NCBI BLAST server + 2.8. The 3D structure of the topmost hit with identity > 39% was retrieved from the Protein Data Bank [76]. The topmost hit 3PM9_A corresponding to *Rhodopseudomonas palostris* (RhopaDH) protein was then used as a template for modelling the putative SbDLDH proteins using the Swiss Model server [77]. The modelled structures were then visualised and compared for similarity to the previously characterised *E.coli* D-LDH (PDB ID: 1F0X) using the PyMOL 2.2 software.

### Plant material and stress treatment for quantitative real-time PCR analysis

*Sorghum bicolor* (L.) Moench (Maharashtra Hybrid) seeds were grown hydroponically under controlled conditions in a growth chamber maintained at 28 °C. Seven day old seedlings were exposed to different abiotic stresses such as salinity, cold, drought and heat. The seedlings were kept at 42 °C and 6 °C for heat and cold stress, respectively. For salinity stress, seedlings were subjected to 150 mM NaCl treatment. The treated seedlings were harvested after 6 h of treatment. For drought stress, water was withheld for a period of 48 h after which the seedlings were harvested. Untreated seedlings were used as control.

### Expression profiling of *SbGLY* and *SbDLDH* genes under different abiotic stresses

Total RNA was isolated using TRIzol™ reagent (Sigma Adrich, USA) as per the manufacturer’s protocol. First strand cDNA was synthesised using RevertAid first strand cDNA synthesis kit (Thermo Fischer Scientific, USA). Primers used for the experiment are listed in Additional file 8: Table S2. The qRT-PCR was performed employing ABI 7500 Real Time PCR System and software (PE Applied Biosystems). The specificity of the amplification was tested by dissociation curve analysis. Three technical replicates were analysed for each sample. The relative expression ratio of each of the candidate genes was calculated using the delta Ct value method [78]. The eEF-1α gene was used as a reference for normalization of data.

## Supplementary information

**Additional file 1: Figure S1.** Determination of alternate splicing of putative functionally active SbGLYI transcripts. (A) Depiction of primer designing scheme. (B) Details of primers used for the determination of spliced variants of *SbGLYI* transcripts and respective amplification details. (C) Gel showing the amplification of *SbGLYI* transcripts.

**Additional file 2: Figure S2.** Alignment of SbGLYI-8/8.1 with its rice and *Arabidopsis* orthologs. Box indicates the location of putative nuclear localisation signal (NLS) sequences.

**Additional file 3: Figure S3.** Domain sequence alignment of predicted GLYI proteins from sorghum and rice. Conserved metal binding sites have been marked with an asterisk and region specific for Zn^2+^-dependent isoforms has been marked with a red line. Each domain of the two glyoxalase domain (PF00903)-containing putative GLYI proteins has been represented as a separate sequence, with domains being indicated by numbers 1 or 2 suffixed to the protein name thereby, indicating first and second domain, respectively.

**Additional file 4: Figure S4.** Multiple sequence alignment of predicted SbGLYII proteins with GLYII proteins from rice. Conserved THHHXDH and C/GHT motifs have been highlighted in blue (for active GLYII) and green (for SDO) colours and indicated by a bar.

**Additional file 5: Table S1.** List of FAD-binding-4 containing oxido-reductase superfamily members.

**Additional file 6: Figure S5.** Multiple sequence alignment of predicted SbDLDH proteins with the previously characterised rice and *Arabidopsis* D-LDH proteins.

**Additional file 7: Figure S6.** Phylogenetic tree representing all putative FAD_binding_4 oxido-reductase superfamily proteins in *Sorghum bicolor*. Differently coloured rings in the circular tree represent different domains present in the respective proteins.

**Additional file 8: Table S2.** List of primers used in the study.

**Additional file 9.** Glyoxalase I protein sequences from various species used for the phylogenetic analysis.

**Additional file 10.** Glyoxalase II protein sequences from various species used for the phylogenetic analysis.

**Additional file 11.** D-LDH protein sequences used for the phylogenetic analysis.

## Data Availability

The datasets supporting the conclusions of this article are included within the article and its additional files. The sequence data was obtained from Phytozome v12 (https://phytozome.jgi.doe.gov) for *Sorghum bicolour*, *Medicago truncatula* and *Glycine max*. For rice and *Arabidopsis,* sequence data was retrieved from RGAP (http://rice.plantbiology.msu.edu/) and TAIR (https://www.arabidopsis.org/) database, respectively. The sequences used in the study have been provided as Additional files 9, 10 and 11.

## Abbreviations

ABA: Abscisic acid
CYTc: Cytochrome c
D-LDH: D-lactate dehydrogenase
FAD: Flavin adenine dinucleotide
GLYI: Glyoxalase I
GLYII: Glyoxalase II
GSH: Glutathione
MG: Methylglyoxal
NLS: Nuclear localisation signal
PEG: Polyethylene glycol
Sb: *Sorghum bicolor*
SLG: S-D-lactoylglutathione
