# Supplementary material for: From methylglyoxal to pyruvate: a genome-wide study for the identification of glyoxalases and D-lactate dehydrogenases in Sorghum bicolor
Source: BMC Genomics. 2020 Feb 10;21:145. doi: 10.1186/s12864-020-6547-7 (PMC7011430; doi:10.1186/s12864-020-6547-7)
Supplement: Supplementary file 1 — Additional file 1: Figure S1. Determination of alternate splicing of putative functionally active SbGLYI transcripts. (A) Depiction of primer designing scheme. (B) Details of primers used for the determination of spliced variants of SbGLYI transcripts and respective amplification details. (C) Gel showing the amplification of SbGLYI transcripts. [file 12864_2020_6547_MOESM1_ESM.pdf]

(A)

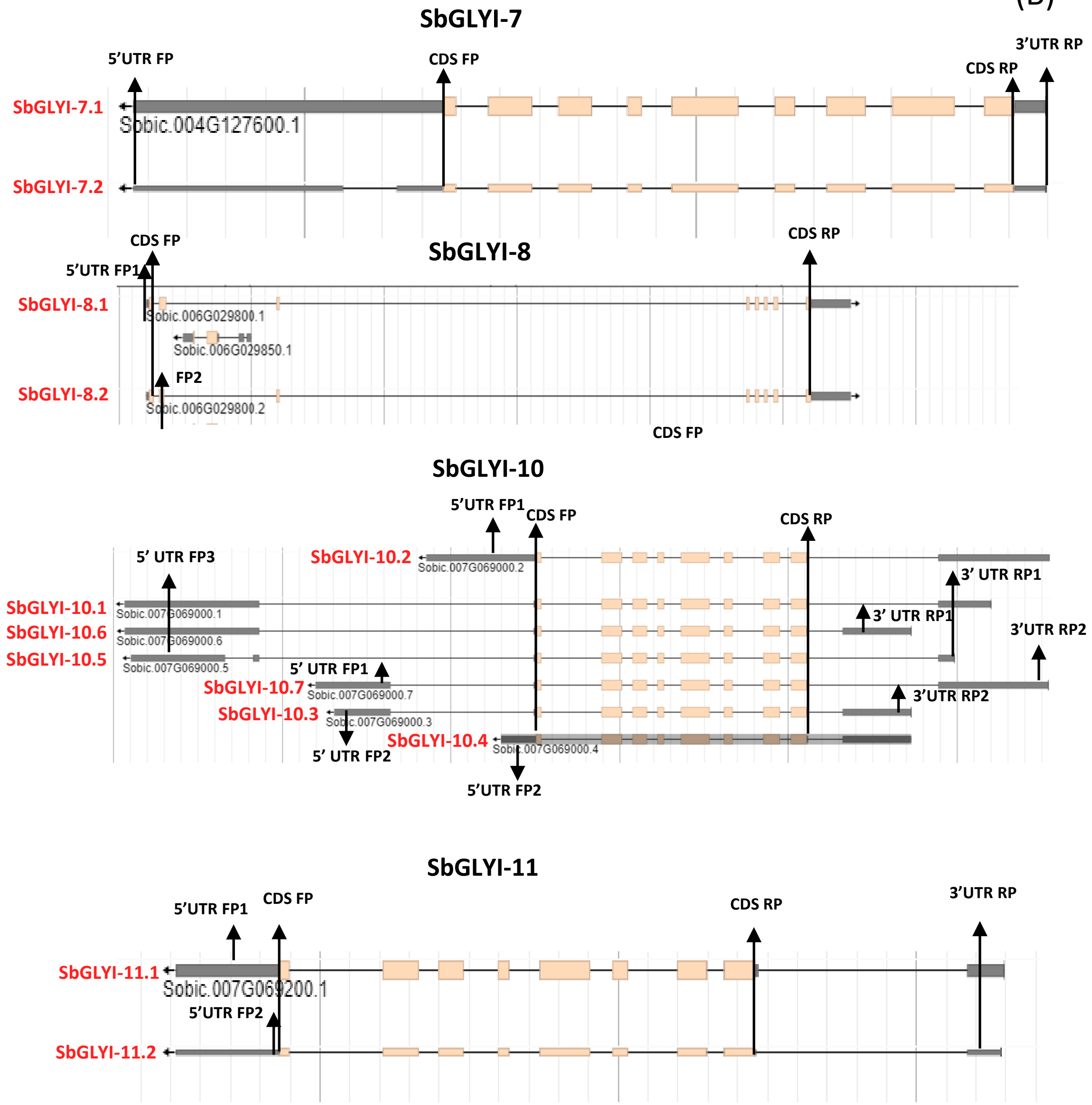

(B)

Transcript IDs	Primer combination	Primer code	Amplicon Size	Amplification
SbGLYI-7	CDS FP & CDS RP	A	1041 bp	No
SbGLYI-7.1	CDS FP & 3'UTR RP	B	1413 bp	No
SbGLYI-7.1	5'UTR FP & CDS RP	C	1141 bp	No
SbGLYI-7.2	CDS FP & 3'UTR RP	D	1241 bp	No
SbGLYI-7.2	5'UTR FP & CDS RP	E	1141 bp	No
SbGLYI-8.1	CDS FP & CDS RP	F	681 bp	Yes
SbGLYI-8.1	5'UTR FP1&CDS RP	G	698 bp	Yes
SbGLYI-8.2	FP2&CDS RP	H	495bp	Yes
SbGLYI-10	CDS FP & CDS RP	I	873 bp	Yes
SbGLYI-10.2/10.7	5'UTR FP1 & CDS RP	J	1318 bp	No
SbGLYI-10.3/10.4	5'UTR FP2 & CDS RP	K	1177 bp	No
SbGLYI-10.1/10.5/10.6	5'UTR FP3 & CDS RP	L	993/993/1177 bp	No
SbGLYI-10.3/10.7	CDS FP1 & 3'UTR RP2	M	999 bp	No
SbGLYI-10.1/10.5/10.6	CDS FP1 & 3'UTR RP1	N	1454/1247/1453 bp	No
SbGLYI-11	CDS FP & CDS RP	O	885 bp	Yes
SbGLYI-11.1	5'UTR FP1 & CDS RP	P	1042 bp	No
SbGLYI-11.2	5'UTR FP2 & CDS RP	Q	891 bp	Yes

(C)

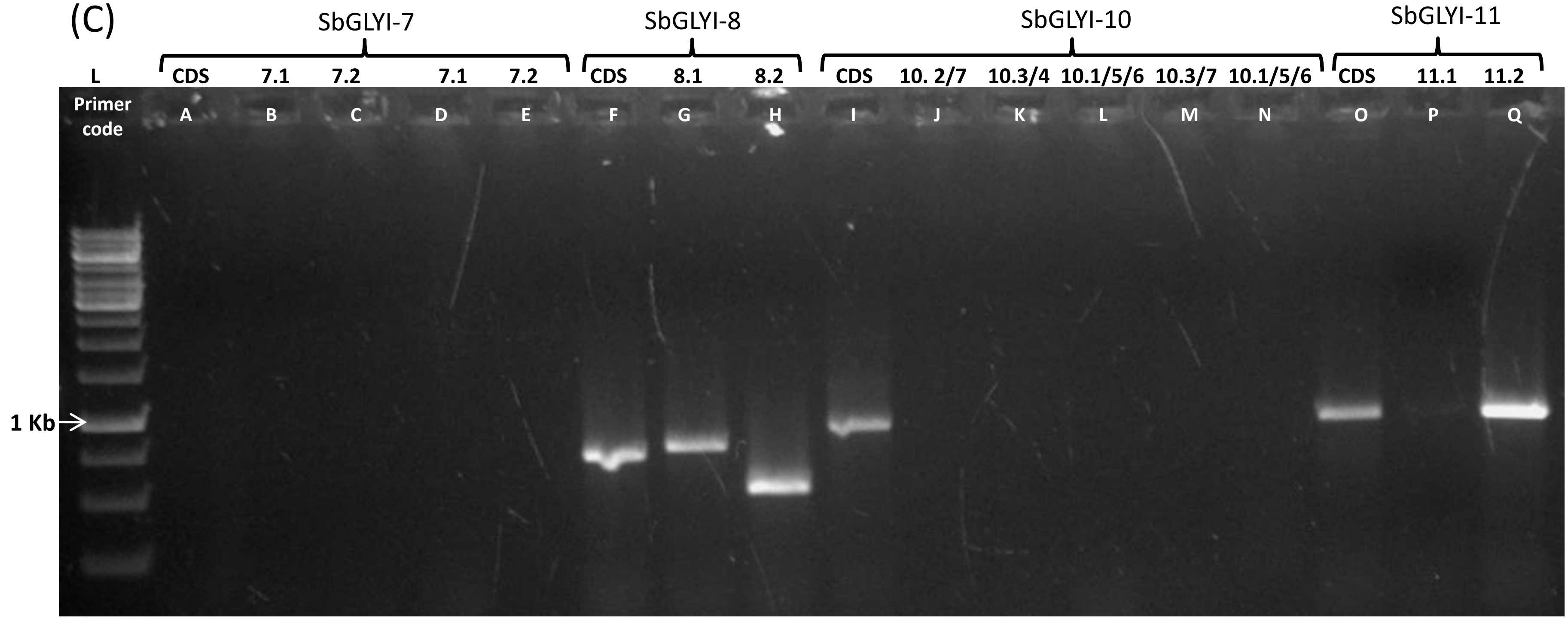

*Primer code indicate primer combinations used for PCR as specified in Figure S1B

Figure S1
