# Supplementary material for: From methylglyoxal to pyruvate: a genome-wide study for the identification of glyoxalases and D-lactate dehydrogenases in Sorghum bicolor
Source: BMC Genomics. 2020 Feb 10;21:145. doi: 10.1186/s12864-020-6547-7 (PMC7011430; doi:10.1186/s12864-020-6547-7)
Supplement: Supplementary file 2 — Additional file 2: Figure S2. Alignment of SbGLYI-8/8.1 with its rice and Arabidopsis orthologs. Box indicates the location of putative nuclear localisation signal (NLS) sequences. [file 12864_2020_6547_MOESM2_ESM.pdf]

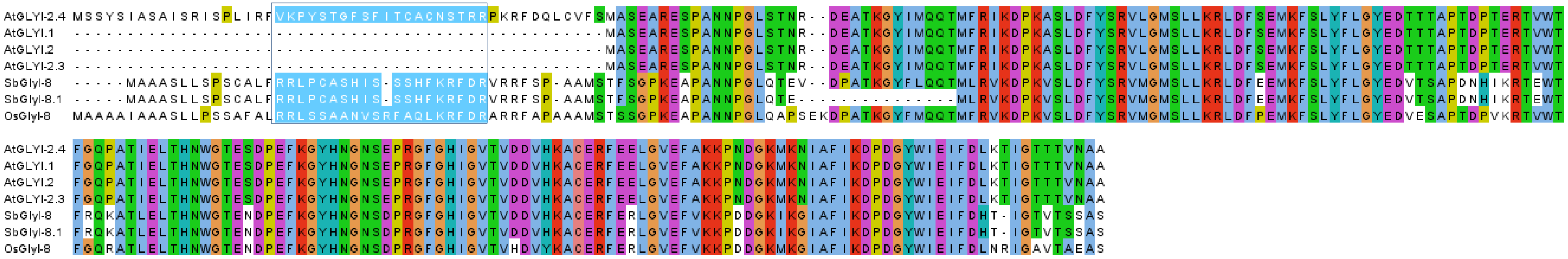

AtGLYI-2.4 MSSYSIASAISRISPLIRFVKPYSTGFSFITCACNSTRRPKRFDQLCVFSMASEARESPANNPGLSTNR--DEATKGYIMQQTMFRIKDPKASLDFYSRVLGMSLLKRLDFSEMKFSLYFLGYEDTTTAPTDPTERTVWT
AtGLYI.1 --MASEARESPANNPGLSTNR--DEATKGYIMQQTMFRIKDPKASLDFYSRVLGMSLLKRLDFSEMKFSLYFLGYEDTTTAPTDPTERTVWT
AtGLYI.2 --MASEARESPANNPGLSTNR--DEATKGYIMQQTMFRIKDPKASLDFYSRVLGMSLLKRLDFSEMKFSLYFLGYEDTTTAPTDPTERTVWT
AtGLYI-2.3 --MASEARESPANNPGLSTNR--DEATKGYIMQQTMFRIKDPKASLDFYSRVLGMSLLKRLDFSEMKFSLYFLGYEDTTTAPTDPTERTVWT
SbGlyI-8 -----MAAASLLSPSCALFRRLPCASHIS-SSHFKRFDRVRRFSP-AAMSTFSGPKEAPANNPGLQTEV--DPATKGYFLQQTMLRVKDPKVSLDFYSRVMGMSLLKRLDFEEMKFSLYFLGYEDVTSAPDNHIKRTEWT
SbGlyI-8.1 -----MAAASLLSPSCALFRRLPCASHIS-SSHFKRFDRVRRFSP-AAMSTFSGPKEAPANNPGLQTE---------------MLRVKDPKVSLDFYSRVMGMSLLKRLDFEEMKFSLYFLGYEDVTSAPDNHIKRTEWT
OsGlyI-8 MAAAAIAAASLLPSSAFALRRLSSAANVSRFAQLKRFDRARRFAPAAAMSTSSGPKEAPANNPGLQAPSEKDPATKGYFMQQTMFRVKDPKVSLDFYSRVMGMSLLKRLDFPEMKFSLYFLGYEDVESAPTDPVKRTVWT
AtGLYI-2.4 FGQPATIELTHNWGTESDPEFKGYHNGNSEPRGFGHIGVTVDDVHKACERFEELGVEFAKKPNDGKMKNIAFIKDPDGYWIEIFDLKTIGTTTVNAA
AtGLYI.1 FGQPATIELTHNWGTESDPEFKGYHNGNSEPRGFGHIGVTVDDVHKACERFEELGVEFAKKPNDGKMKNIAFIKDPDGYWIEIFDLKTIGTTTVNAA
AtGLYI.2 FGQPATIELTHNWGTESDPEFKGYHNGNSEPRGFGHIGVTVDDVHKACERFEELGVEFAKKPNDGKMKNIAFIKDPDGYWIEIFDLKTIGTTTVNAA
AtGLYI-2.3 FGQPATIELTHNWGTESDPEFKGYHNGNSEPRGFGHIGVTVDDVHKACERFEELGVEFAKKPNDGKMKNIAFIKDPDGYWIEIFDLKTIGTTTVNAA
SbGlyI-8 FRQKATLELTHNWGTENDPEFKGYHNGNSDPRGFGHIGVTVDDVHKACERFERLGVEFVKKPDDGKIKGIAFIKDPDGYWIEIFDHT-IGTVTSSAS
SbGlyI-8.1 FRQKATLELTHNWGTENDPEFKGYHNGNSDPRGFGHIGVTVDDVHKACERFERLGVEFVKKPDDGKIKGIAFIKDPDGYWIEIFDHT-IGTVTSSAS
OsGlyI-8 FGQRATLELTHNWGTENDPEFKGYHNGNSDPRGFGHIGVTVHDVYKACERFERLGVEFVKKPDDGKMKGIAFIKDPDGYWIEIFDLNRIGAVTAEAS

Figure S2
