# Supplementary material for: From methylglyoxal to pyruvate: a genome-wide study for the identification of glyoxalases and D-lactate dehydrogenases in Sorghum bicolor
Source: BMC Genomics. 2020 Feb 10;21:145. doi: 10.1186/s12864-020-6547-7 (PMC7011430; doi:10.1186/s12864-020-6547-7)
Supplement: Supplementary file 4 — Additional file 4: Figure S4. Multiple sequence alignment of predicted SbGLYII proteins with GLYII proteins from rice. Conserved THHHXDH and C/GHT motifs have been highlighted in blue (for active GLYII) and green (for SDO) colours and indicated by a bar. [file 12864_2020_6547_MOESM4_ESM.pdf]

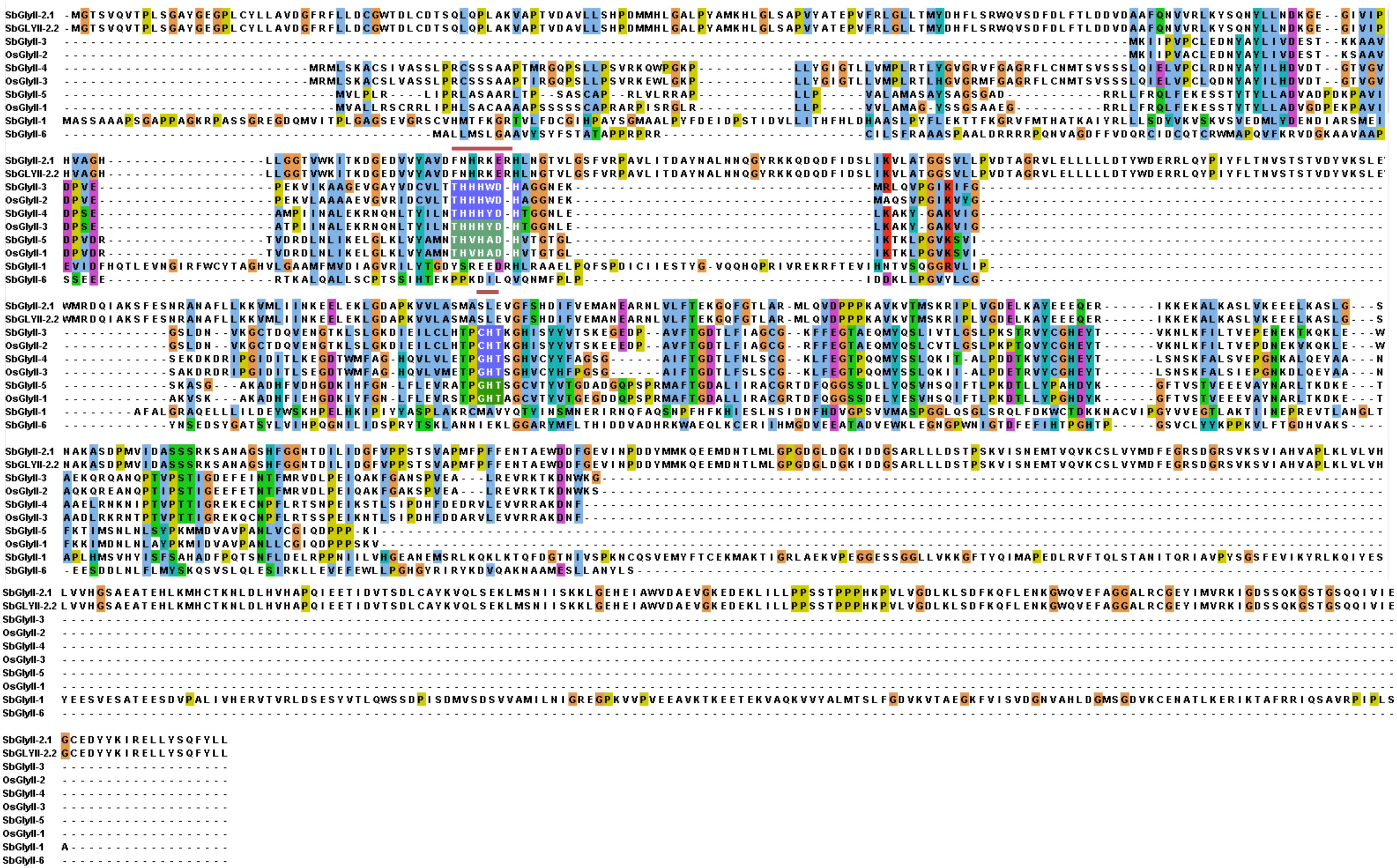
SbGlyII-2.1
SbGLYII-2.2
SbGlyII-3
OsGlyII-2
SbGlyII-4
OsGlyII-3
SbGlyII-5
OsGlyII-1
SbGlyII-1
SbGlyII-6

Figure S4
