# Supplementary material for: From methylglyoxal to pyruvate: a genome-wide study for the identification of glyoxalases and D-lactate dehydrogenases in Sorghum bicolor
Source: BMC Genomics. 2020 Feb 10;21:145. doi: 10.1186/s12864-020-6547-7 (PMC7011430; doi:10.1186/s12864-020-6547-7)
Supplement: Supplementary file 5 — Additional file 5: Table S1. List of FAD-binding-4 containing oxido-reductase superfamily members. [file 12864_2020_6547_MOESM5_ESM.pdf]

TABLE S1. List of FAD-binding-4 containing oxido-reductase superfamily members.

Locus Name	Transcripts	Coordinate (5'-3')	Transcript length (bp)	CDS (bp)	Protein		
					Length (aa)	MW (kDa)	pI
Sobic.001G189500	Sobic.001G189500.1	16747692..16753403	2184	1881	626	66.46	8.08
Sobic.001G215600	Sobic.001G215600.3	19906995..19915069	5248	1575	524	58.07	6.59
Sobic.002G042400	Sobic.002G042400.1	4037195..4048696	2045	1791	596	64.8	6.69
Sobic.002G058500	Sobic.002G058500.1	5652091..5665483	2363	1686	561	61.19	7.03
	Sobic.002G058500.2	5652091..5665483	2360	1686	561	61.19	7.03
Sobic.003G029900	Sobic.003G029900.1	2655907..2661093	2286	1734	577	61.63	6.1
Sobic.003G036700	Sobic.003G036700.1	3381538..3384118	2016	1581	526	57.12	5.06
Sobic.003G313800	Sobic.003G313800.1	64196600..64200920	2430	1647	548	59.11	6.57
	Sobic.003G313800.2	64198244..64200520	1387	1317	438	46.78	6.65
Sobic.003G421100	Sobic.003G421100.1	72617865..72620838	2606	1572	523	58.33	7.21
Sobic.004G093900	Sobic.004G093900.1	8035899..8038098	1951	1614	537	58.9	7.61
Sobic.004G094000	Sobic.004G094000.1	8065896..8068092	1956	1605	534	58.71	7.34
Sobic.004G104400	Sobic.004G104400.1	9835636..9837381	1746	1746	581	62.68	7.98
Sobic.004G128800	Sobic.004G128800.1	16299887..16305971	2068	1752	583	61.97	7.11
Sobic.004G355600	Sobic.004G355600.1	68290516..68292974	2232	1362	453	52.36	7.4
	Sobic.004G355600.2	68290516..68292974	2075	1362	453	52.36	7.4
Sobic.005G031000	Sobic.005G031000.1	2767610..2771688	2142	1758	585	65.8	7.55
	Sobic.005G031000.2	2767610..2771688	1897	1308	435	48.34	9.38
Sobic.005G031000	Sobic.005G031000.3	2767610..2771688	2153	1089	362	40.05	9.91
Sobic.005G119900	Sobic.005G119900.1	52195863..52197992	2130	1587	528	57.15	7.99
	Sobic.005G119900.2	52195895..52197611	1333	1203	400	43.2	7.26
Sobic.006G056300	Sobic.006G056300.1	39996099..39997706	1608	1608	535	57.24	9.36
Sobic.006G151500	Sobic.006G151500.1	51224273..51229767	2219	1593	530	57.27	5.43
	Sobic.006G151500.2	51224273..51229784	4199	1317	438	47.26	4.99
Sobic.007G003200	Sobic.007G003200.2	294053..295694	1641	1641	546	60.28	7.67
Sobic.007G017500	Sobic.007G017500.1	1502262..1504616	2068	1806	601	65.09	6.36
Sobic.007G048200	Sobic.007G048200.1	4806831..4809237	2407	1680	559	58.83	8.43
Sobic.007G048300	Sobic.007G048300.1	4811033..4812703	1671	1671	556	59.06	7.38
Sobic.007G048400	Sobic.007G048400.1	4817244..4818884	1641	1641	546	58.04	8.41
Sobic.007G048600	Sobic.007G048600.1	4829857..4831587	1731	1731	576	62.05	8
Sobic.007G151400	Sobic.007G151400.1	58341777..58345411	2233	1638	545	58.28	5.49
Sobic.008G145900	Sobic.008G145900.1	57770289..57772936	2089	1815	604	65.74	7.61
Sobic.009G118700	Sobic.009G118700.1	46471299..46473626	1943	1569	522	58.31	6.71
Sobic.010G162400	Sobic.010G162400.1	47964514..47966115	1602	1602	533	58.66	8.51
Sobic.010G162500	Sobic.010G162500.1	47975241..47980077	3581	1611	536	58.75	9.7
Sobic.010G162700	Sobic.010G162700.1	48073190..48075092	1903	1581	526	57.71	9.67
Sobic.010G162800	Sobic.010G162800.1	48091245..48093395	2151	1620	539	59.13	6.4
Sobic.010G162900	Sobic.010G162900.1	48098731..48102167	2622	1581	526	57.7	6.94
Sobic.010G163000	Sobic.010G163000.1	48114038..48116057	2020	1590	529	57.57	9.44
Sobic.010G173600	Sobic.010G173600.1	50881171..50883589	2252	1704	567	62.15	6.8

Sobic.010G274400	Sobic.010G274400.1	60762763..60764751	1989	1626	541	59.76	6.88
Sobic.010G277300	Sobic.010G277300.1	61014181..61018074	2197	1734	577	66.91	8.6
	Sobic.010G277300.2	61014032..61018074	2107	1686	561	65.03	8.46

*ER-Endoplasmic reticulum, mito-mitochondria, chlo-chloroplast, Nucl-nucleus, pero-peroxisomes, vacu-vacuoles, plasma-plasma membrane, cyto- cytoplasm, plas- plastid, golg-golgi bodies.
