# Supplementary material for: From methylglyoxal to pyruvate: a genome-wide study for the identification of glyoxalases and D-lactate dehydrogenases in Sorghum bicolor
Source: BMC Genomics. 2020 Feb 10;21:145. doi: 10.1186/s12864-020-6547-7 (PMC7011430; doi:10.1186/s12864-020-6547-7)
Supplement: Supplementary file 6 — Additional file 6: Figure S5. Multiple sequence alignment of predicted SbDLDH proteins with the previously characterised rice and Arabidopsis D-LDH proteins. [file 12864_2020_6547_MOESM6_ESM.pdf]

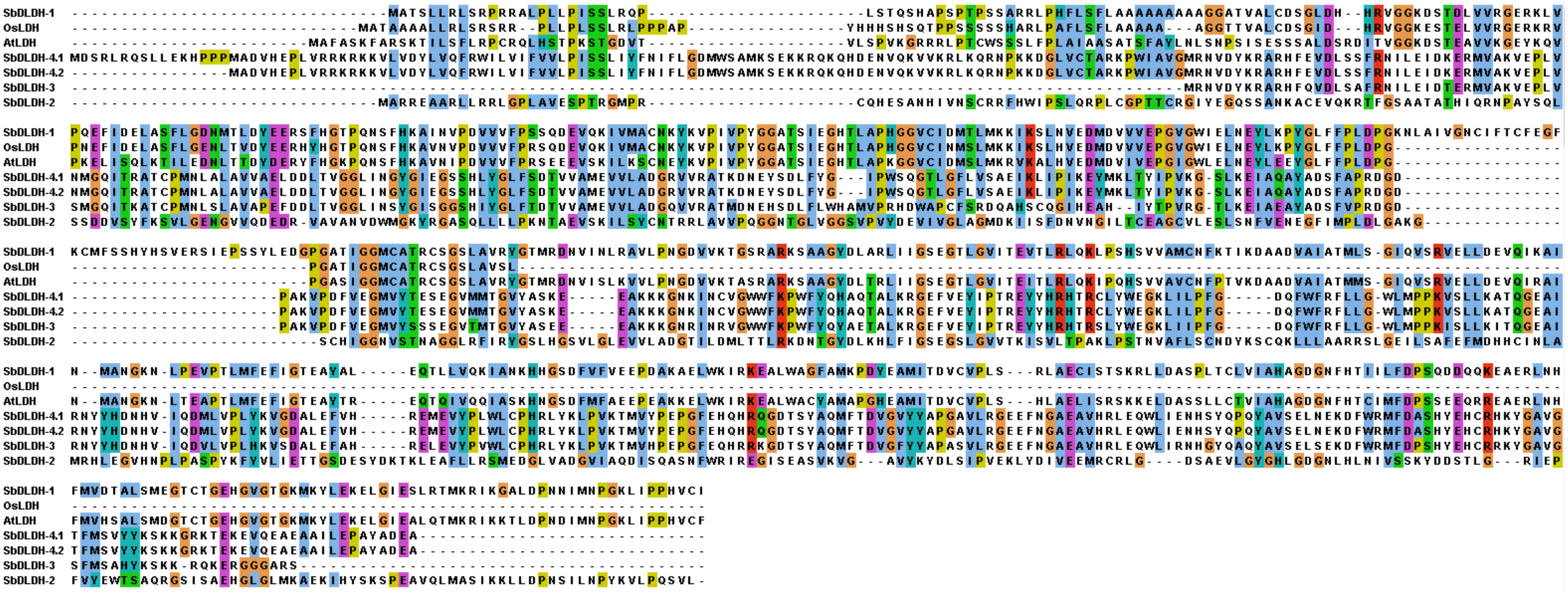

Figure S5
