# Supplementary figures and images for: From methylglyoxal to pyruvate: a genome-wide study for the identification of glyoxalases and D-lactate dehydrogenases in Sorghum bicolor
Source: BMC Genomics. 2020 Feb 10;21:145. doi: 10.1186/s12864-020-6547-7 (PMC7011430; doi:10.1186/s12864-020-6547-7)

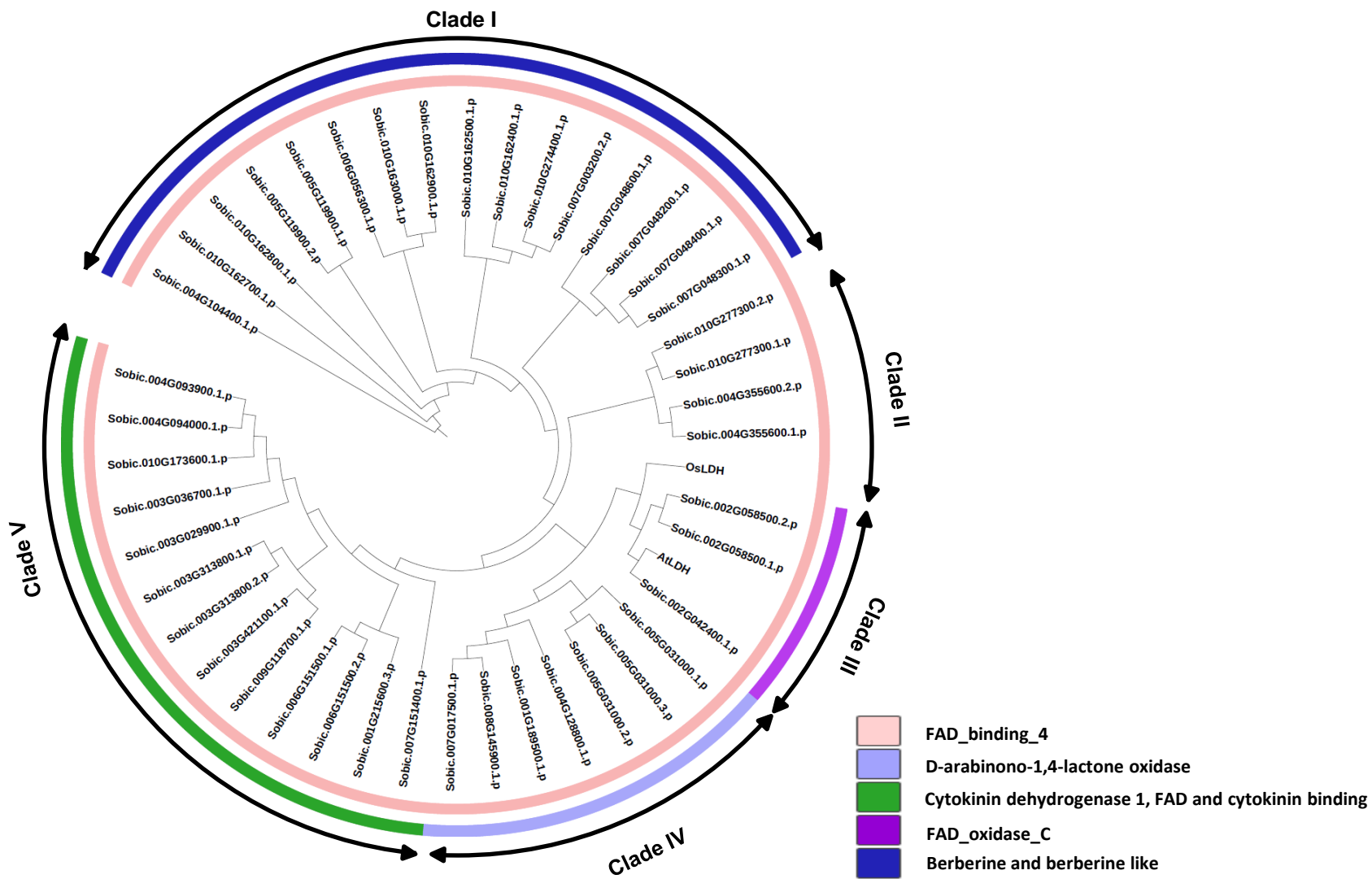

**Figure S6**

Supplement: Supplementary file 7 — Additional file 7: Figure S6. Phylogenetic tree representing all putative FAD_binding_4 oxido-reductase superfamily proteins in Sorghum bicolor. Differently coloured rings in the circular tree represent different domains present in the respective proteins. [file 12864_2020_6547_MOESM7_ESM.pdf]
