# Supplementary material for: From methylglyoxal to pyruvate: a genome-wide study for the identification of glyoxalases and D-lactate dehydrogenases in Sorghum bicolor
Source: BMC Genomics. 2020 Feb 10;21:145. doi: 10.1186/s12864-020-6547-7 (PMC7011430; doi:10.1186/s12864-020-6547-7)
Supplement: Supplementary file 8 — Additional file 8: Table S2. List of primers used in the study. [file 12864_2020_6547_MOESM8_ESM.pdf]

Table S2. List of primers used in the study

S.No.	Primer names	Sequence
For qRT-PCR		
1	**SbEIFα-FP**	5’-CAACTTTGTCACCCGCGATGA-3’
2	**SbEIFα-RP**	5’-TCCAGAAACCTTAGCAGCCCA-3’
3	**SbGLYI-7-qRT-FP**	5’-GCAACAGCATTCCATCTTGA-3’
4	**SbGLYI-7-qRT-RP**	5’-ACGCATCTTGGTTTTTCCTG-3’
5	**SbGLYI-8_8.1-qRT-FP**	5’-GGGTTCTGTTGGGGATGTTA-3’
6	**SbGLYI-8-8.1-qRT-RP**	5’-GTAGTGTGTGCCGGGAGAAT-3’
7	**SbGLYI-10.2_4-qRT-FP**	5’-CCCTGCTCGTCTGTGATGTA-3’
8	**SbGLYI-10.2_4-qRT-RP**	5’-CCACCGCAATAGACATGTTG-3’
9	**SbGLYI-11.1_2-qRT-FP**	5’-CCAGTGAGGTTTGGGATTTG-3’
10	**SbGLYI-11.1_2-qRT-RP**	5’-TTTGAGCTTCACTGGCTCCT-3’
11	**SbGLYI-14-qRT-FP**	5’-TCCAATCTACGGCATCACAA-3’
12	**SbGLYI-14-qRT-RP**	5’-GATAGTCCTGAGACGGAGCA-3’
13	**SbGLYII-3-qRT-FP**	5’-ATGGCTGCCACTACTCTGCT-3’
14	**SbGLYII-3-qRT-RP**	5’-TGGCAAACAGACCACAAGTT-3’
15	**SbGLYII-4-qRT-FP**	5’-TGATTGTGATCCCCAGAACA-3’
16	**SbGLYII-4-qRT-RP**	5’-TCATGGAATCCCAAGCTTTC-3’
17	**SbDLDH-1-qRT-FP**	5’-TGCTGACAGTGCAAGCAATA-3’
18	**SbDLDH-1-qRT-RP**	5’-GGCATGAGGAGACGAATGTT-3’
19	**SbDLDH-2-qRT-FP**	5’-AAGCAGGTTAGGGACCCTTC-3’
20	**SbDLDH-2-qRT-RP**	5’-GACGATGTCGCACTTCAAAA-3’
21	**SbDLDH-3-qRT-FP**	5’-GATGCTGATGCGGAGTGATA-3’
22	**SbDLDH-3-qRT-RP**	5’-TGGCCGAAGGTGATTATAGC-3’
23	**SbDLDH-4.1_2-qRT-FP**	5’-CGAATCTGGACGAGATGAGA-3’
24	**SbDLDH-4.1_2-qRT-RP**	5’-CCATGTAGCAAGTCCCGAAT-3’
For determination of transcript variants		
25	**SbGLYI-8.1-5'UTR-FP**	5’-TACAGATCAACCGGCAGCTAG-3’
26	**SbGLYI-8.2-FP**	5’-GACCCCGCCACCAAGGGC-3’
27	**SbGLYI-7_CDS_FP**	5’-ATGAGGACTCTACAGGTGG-3’
28	**SbGLYI-7_CDS_RP**	5’-CTACTCCAGTTCCTTGGCAA-3’
29	**SbGLYI-7.1/7.2-5'UTR-FP**	5’-CGTCCACAGCAAATACATATCG-3’
30	**SbGLYI-7.1/7.2-5'UTR-RP**	5’-AGAAAGTGAGGTACTATCTGAC-3’
31	**SbGLYI-10_CDS_FP**	5’-ATGGCAACTGGTAGTGAAGCCG-3’
32	**SbGLYI-10_CDS_RP**	5’-TCACTGAAGTTCCTTGAGGAAGT-3’
33	**SbGLYI-10.2/10.7-5'UTR-FP1**	5’-AGAGAATCTTTAAAAAAATTTG-3’
34	**SbGLYI-10.3/10.4-5'UTR-FP2**	5’-CCTTTGGGCCTTCATCCTCAA-3’
35	**SbGLYI-10.1/10.5/10.6-5'UTR-FP3**	5’-GGAGGAAGCCAAAGCCCAAAG-3’
36	**SbGLYI-10.1/10.5/10.6_3'UTR_RP1**	5’-CAAAGCCCTCTCTGCAATTCCTT-3’
37	**SbGLYI-10.3/10.7_3'UTR_RP2**	5’-CTTCTGGCAACAGAGAACTGT-3’
