# Supplementary material for: From methylglyoxal to pyruvate: a genome-wide study for the identification of glyoxalases and D-lactate dehydrogenases in Sorghum bicolor
Source: BMC Genomics. 2020 Feb 10;21:145. doi: 10.1186/s12864-020-6547-7 (PMC7011430; doi:10.1186/s12864-020-6547-7)
Supplement: Supplementary file 9 — Additional file 9. Glyoxalase I protein sequences from various species used for the phylogenetic analysis. [file 12864_2020_6547_MOESM9_ESM.docx]

>SorbicGlyI-1

MATLQLNHIARETSDVVRLAAFYEAVLGFERIPSPTYSGFQVAWLRLPSSPDVALHLIERDPAAAPAAVGPGAEGAPPSQLPRRHHLAFSVADYDGFVTGLKARGTEVFEKTQPDGRTRQVFFFDPDGNGLEVTSAGAGSAK*

>SorbicGlyI-1.1

MATLQLNHIARETSDVVRLAAFYEAVLGFERIPSPTYSGFQVAWLRLPSSPDVALHLIERDPAAAPAAVGPGAEGAPPSQLPRRHHLAFSVADYDGFVTGLKARGTEVFEKTQPDGRTRQVFFFDPDGNTSANTACKMRWKLDMFLGQASFRKINKSEALLTLLFG*

>SorbicGlyI-2

MASGGLKPRLGYIIMYTSDVEKAASFYDAAFGYTVRRLDESRKWAELESGATTIAFTPLHQRETDARSGEVQLPDSAAAERGALEVCFAYEDVDAAYRRAVENGAVPVSAPEEKPWGQKSGFVRDMDGNVVRIASYVRE*

>SorbicGlyI-3

MNAHAHPVVASTAFAHALLPNSSSIRSRRTPLRPIFAVPPPALGQLPTTLRATTKTLLHPTTTPPVVTTTADRAESVFAGTGDRFHVMDFHHVEFWCADAASAAGRFSFALGVPLAAQSDLTTGNTAHASRLLRSRSGPLALLFTAPYARHIGARPDADATSASSPVVPSFSADAARRFAADYGGLAVRAVAVRVSDAAEAFRASVAAGARPAFAPAELGHGFVFAEVELYGDAVLRFVSYPDDTGGVAFLPGFENVANSSACPAPDYGLNRFDHIVGGVPDLAPVAAYIAGFTGFHEFDRVNGDEIGTAESSLNGLVLADSSEKVLLTLLEPVQGTKRRSQIQTFLDHHGGPGVQHLAMTSDDLLGTLREIRARSSMGGFELLPPPPPSYYDGVKRLAGDVLSEAQINECQELGVRVDRADNGGVVLQTFTKAAGDRPTLLLEFIQRIGCVEIDENGKEYQRGGCGGFAKDNVIHLVKSIEDYDKTLDAPAHVAS*

>SorbicGlyI-4

MGEACRDAAAAGRQDEAGGGGGRHHAENEVVGDLAASAKLYEDVPAFPMMALNHISRLCESVDASVRFYVKALGFVLIHRPPALDFSGAWLFNYGVGIHLVQRDDARRAPDVRPETELDPMDNHVSFQCEDMGAMERRLQELHIRYMKRTINEEEGSPIDQLFFRDPDGFMIEICNCENLELVPAGALGRLRLPRDRHNPPVRTGTGGAE*

>SorbicGlyI-5

MVNTMGVEPVQRGAGLPLAALNHISVVCRCLESSLRFYRDVLGFVPIRRPGSFDFHGAWLFNYGIGVHLLQAEDASMPPKKTEINPKDNHISFQCESMEAVQRRLKELGIRYVQRRVEEGGIYVDQLFFHDPDGFMVEVCTCDNLPIVPLVPEGHAILGLQQPAAAPACKRPPAAALRQQAPAPLPVPVAVPTPVPAEQCIPAKAGGGSCVGEVEASIPACAMRSCPEHACV*

>SorbicGlyI-6

MPPTPTTAAATGAAVAAASAEQAAFRLVGHRNFVRVNPRSDRFHTLAFHHVELWCADAASAAGRFSFGLGAPLAARSDLSTGNTAHASLLLRSGALAFLFTAPYAHGADAATASLPSFSAAEARRFAADHGLAVRAVALRVADAEDAFRASVAAGARPAFEPVELGLGFRLAEVELYGDVVLRYVSYPDDADASFLPGFVGVTSPGAADYGLRRFDHIVGNVPELAPAAAYFAGFTGFHEFAEFTAEDVGTTESGLNSMVLANNAENVLLPLNEPVHGTKRRSQIQTYLDHHGGPGVQHMALASDDVLRTLREMQARSAMGGFEFMAPPAPEYYDGVRRRAGDVLTEAQIKECQELGVLVDRDDQGVLLQIFTKPVGDRPTLFLEIIQRIGCMEKDEKGQEYQKGGCGGFGKGNFSQLFKSIEDYEKSLEAKQAAAAQGS*

>SorbicGlyI-7

MRTLQVAAGRGAVACAATPVPRRSLLLSTAAAGAALQSEQVPLRLTRNVPGAAAKFRASADAAQAATFATTDEAFSWAKKDNRRLLHVVYRVGDIDKTIKFYTECLGMKLLRKRDIPEEKYTNAFLGYGPEESHFVVELTYNYGVDKYDVGAGFGHFGIGVEDVAKTVELIRAKGGKVTREPGPVKGGKTVIAFVEDPDGYIFEIIERPGTPEPLSQVMLRVGDLDRAISFYEKACGMELLLKRDNPEYKYTVAMMGYGPEDRNAVLELTYNYGVTEYAKGNAYAQIAIGTDDVYKTAEVVKLSGGQVVREPGPLPGINTKITSILDPDGWKSVFVDNIDFAKELE*

>SorbicGlyI-8

MAAASLLSPSCALFRRLPCASHISSSHFKRFDRVRRFSPAAMSTFSGPKEAPANNPGLQTEVDPATKGYFLQQTMLRVKDPKVSLDFYSRVMGMSLLKRLDFEEMKFSLYFLGYEDVTSAPDNHIKRTEWTFRQKATLELTHNWGTENDPEFKGYHNGNSDPRGFGHIGVTVDDVHKACERFERLGVEFVKKPDDGKIKGIAFIKDPDGYWIEIFDHTIGTVTSSAS*

>SorbicGlyI-8.1

MAAASLLSPSCALFRRLPCASHISSSHFKRFDRVRRFSPAAMSTFSGPKEAPANNPGLQTEMLRVKDPKVSLDFYSRVMGMSLLKRLDFEEMKFSLYFLGYEDVTSAPDNHIKRTEWTFRQKATLELTHNWGTENDPEFKGYHNGNSDPRGFGHIGVTVDDVHKACERFERLGVEFVKKPDDGKIKGIAFIKDPDGYWIEIFDHTIGTVTSSAS*

>SorbicGlyI-9

MDFHEKDEHDPAPPIPLVRLNHVSFQCESVEASVAFYQRVLGFQLVKRPASLDFRGAWLHKYGMGIHLLQRGSDSSAPAAAARPPVINPKGNHISFQCTDMALMKARLGDMELEFVAARVRDGDTVVEQLFFHDPDGNVIEVCDCEKLPVIPLADAGGADLPNLLLPVKTNVHG*

>SorbicGlyI-10

MATGSEAVKPAEAVLGWHKQDSKRMLHAVYRVGDLDRTIKYYTECFGMKLLRKRDVPDEKYTNAFLGFGPEDTNFALELTYNYGVDKYDIGEGFGHFGIANEDVYKLADHIKSKGGNITREPGPVKGGSTVIAFAQDPDGYRFALIQRAEIHDPLCQVMLRVGDLERSIKFYEKALGMKLLTKKDVPDYKHTIAKLGYAEEDKTTALELIYNYGVTEYSKGNAYAQIAIGTNDVYKSAEAVDLATKELGGKILRQPGPLPGINTKIASFVDPDGWKVVLVDHADFLKELQ*

>SorbicGlyI-11

MATGSEASKPAEVPAETVLDWHKQDNKRMLHAVYRVGDLDRTIKYYTECFGMKLLRKRDIPEEKYTNAFLGFGPEDTNFAVELTYNYGVDKYDIGTGFGHFAIANEDVYKLAENIKSKGGKITREPGPVKGGSTVIAFAQDPDGYMFELIQRAETPEPLCQVMLRVGDLERSIKFYEKALGLKLLRKKDVPDYKYTIAMLGYADEDKTTVLELTYNYGVTEYSKGNAYAQVAIGTNDVYKSAEAVELATKELGGKILRQPGPLPGINTKIASFVDPDGWKVVLVDNTDFLRELH*

>SorbicGlyI-12

MATRCLAVAGSLPLLSASTAAAAAARAQPLPSAAAPRRALTRLSVATGGDQQLVTAQDPAHEPDYGVISLHHVGILCENLERSMAFYKDLLGLEVNPARPNDKLPYRGAWLWVGSEMIHLMELPNPDPLTGRPEHGGRDRHTCIAIKDVLKLKEIFDKAGISYTLSKSGRPAIFARDPDGNALEFTQV*

>SorbicGlyI-13

MVSSTTMAAASCGGVRAAGVLPLASLNHISIVCRNVEASLRFYTDVLGFVPIRRPGSFDFDGAWLFNYGIGIHLLQSEDPGSLPEKGEINPKDNHISFQCESMVAVERRLKEMGIPYVQRCVEEGGINVDQIFFHDPDGFMIEICNCDNLPVIPLAGAVQLGSCKRAAAAVVVGKQQQQQSSVVVVVPPPSPPVTATAAAHHQAIRVAEESSSSHISCA*

>SorbicGlyI-14

MARLLLPLPFAAAAAASASSLHLAASRLRLPAVSVARRECLYGGRVVGGVVRAPARLGKRGLCAGAEAGGSAGTVVGQEEALEWVKKDRRRLLHVVYRVGDLDKTIKFYTECLGMKLLRKRDIPEERYTNAFLGYGPEDSHFVVELTYNYGVESYDIGTAFGHFGIAVDDVAKTVELIKAKGGTVTREPGPVKGGKSVIAFIEDPDGYKFELIERGPTPEPLCQVMLRVGDLDRAINFYEKAFGMELLRKRDNPEYKYTIAMMGYGPEDKNAVLELTYNYGVKEYDKGNAYAQIAISTDDVYKTAEAIRVNGGRITREPGPLPGINTKITACTDPDGWKTVFVDNIDFLKELEE*

>SorbicGlyI-15

MAAATATATLRWVLQLHRDVPRAARFYAEGLDFSVNVCTLRWAELQSGPLKLALMHTNDSNLASQRIYSSMLSFTVPDINNTVSKLMALGAELDGPIKYEIHGKVAALRCIDGHMLGLYEPA*

>OsGlyI-1

MVNTTAGVKCGGGGAALPLSTLNHVSLVCRSLSTSLTFYRDFLGFVSVRRPGSFDFDGAWLFNYGIGIHLLQAEDPESMPPNKEINPKDNHISFTCESMEAVQRRLKEMGVRYVQRRVEEGGVYVDQIFFHDPDGFMIEICTCDKLPVVPLDAAAAHSIFAGRSPPPPVACKIRPVKQPSATKLGSVAAGGCVGEVIVVDAINGAAAAGGGGAMS

>OsGlyI-2

MRALPMAAGRAAAVAACASPAVPRRSLLLSTAAAGEPPCRPPADSSSPSKFSRFDRSAVRLLGWTAALQPEPVRLTRGASAAPKLRASPPDAAQAAAAFGSKEEAFAWAKSDNRRLLHVVYRVGDIDRTIKFYTECLGMKLLRKRDIPEEKYTNAFLGYGAEDNHFVVELTYNYGVDKYDIGAGFGHFGIAVDDVAKTVELIRAKGGKVTREPGPVKGGKTVIAFVEDPDGYKFEILERPGTPEPLCQVMLRVGNLDRAISFYEKACGMELLRKRDNPEYKYTVAMMGYGPEDKNAVLELTYNYGVTEYDKGNAYAQIAIGTDDVYKTAEVVKLFGGQVVREPGPLPGINTKITSILDPDGWKSVFVDNIDFAKELE

>OsGlyI-3

MTMTMSNEQGKPEANVRGGRRSGHRVHARHLPGLPTLAAAPARTNGSSWAPGKTEHCTAQHKRRLQAVRDKPQQASVMASEGAVSPAFAYTVVYVKDVAKSAAFYSAAFGYTVRRLDQSHKWAELESGTTTIAFTPLHQRETDALTGAVQLPDSAGERGPVEICFDYADVDAAYRRAVDSGAVPVSPPEQKSWGQKVGYVRDIDGIIVRMGSHVRA

>OsGlyI-4

MATLQLNHVARETDDVRRLAAFYEEVLGFERVASPNYPAFQVAWLRLPGTPGVALHIIERDPAAAPAAVAPGAAGAPPAQLPRRHHLAFSVADYDGFLTGLKARGTDVFEKTQPDGRTRQVFFFDPDGNGLEVTSSGTGDM

>OsGlyI-5

MGSEAPDPAVAASVPLVRLNHVSFQCTSVEKSVDFYRRVLGFELIKRPESLNFNGAWLYKYGMGIHLLQRGDDADGCSIPTRPLPAINPMGNHVSFQCSDMAVMKARLRAMDREFVVRKVWDGETVVDQLFFHDPDGNMIEVCNCENLPVIPLIVASTPGLPELLPPAMQTNVHG

>OsGlyI-6.1

MVNTAAVAAAKGSRGSGLPLASLNHISIVCRSLQESLTFYTDVLGFFPVRRPGSFDFDGAWLFNYGIGIHLLQAEDPDSLPGKTEINPKDNHISFQCESMVAVERRLKELGIPYIQRCVEEGGIYVDQIFFHDPDGFMIEICNCDNLPVVPLGADQPLVMAACKRAAVIKQQQQASSSPATAAAAAQCAVPSSTKAIHVGEEAHISCA

>OsGlyI-7.1

MARLLLPLPIAAAAASRLRLPVLSSSVARREALLFGGRVAAARAPVRLARRGVSAGAEAGGSSSAAAAAQVIGQDEAVEWVKKDRRRMLHVVYRVGDLDKTIKFYTECLGMKLLRKRDIPEERYTNAFLGYGPEDSHFVVELTYNYGVESYDIGTAFGHFGIAVEDVAKTVDLIKAKGGTVTREPGPVKGGKSVIAFIEDPDGYKFELIERGPTPEPLCQVMLRVGDLDHAINFYEKAFGMELLRKRDNPQYKYTIAMMGYGPEDKNAVLELTYNYGVKEYDKGNAYAQIAISTDDVYKTAEVIRQNGGQITREPGPLPGINTKITACTDPDGWKTVFVDNVDFLKELEE

>OsGlyI-8

MAAAAIAAASLLPSSAFALRRLSSAANVSRFAQLKRFDRARRFAPAAAMSTSSGPKEAPANNPGLQAPSEKDPATKGYFMQQTMFRVKDPKVSLDFYSRVMGMSLLKRLDFPEMKFSLYFLGYEDVESAPTDPVKRTVWTFGQRATLELTHNWGTENDPEFKGYHNGNSDPRGFGHIGVTVHDVYKACERFERLGVEFVKKPDDGKMKGIAFIKDPDGYWIEIFDLNRIGAVTAEAS

>OsGLYI-9

MAARCLSSLALLSPSPSSSGKVSAMASPPVPSSAAPRRRPGTRLSVATGGEQLVTAQEASQEPAYGVVSIHHVGILCENLERSMAFYKDLLGLKVNPARPTDKLPYRGAWLWVGSEMIHLMELPNPDPLTGRPEHGGRDRHTCMAIKDVLKLKEIFDKAGIKYTLSKSGRPAIFARDPDGNALEFTQV

>OsGlyI-10

MAGCRRPTTEMGEVCKRVAPSVREEEEEEENGDGGGVDPAAESSSAKLYEDVPEMPLMALNHISRLCKSIDASVRFYVKALGFVLIHRPPALDFNGAWLFNYGVGIHLVQRDDARRAPDVNPGDLDPMDNHISFQCEDMGMMEKRLNEMGIEYMKRTINEEEGSPIDQLFFKDPDGFMIEICNCENLELVPAGALGRLRLPRDRHNPPLRMAAAGNDEA

>OsGlyI-11

MASGSEAEKSPEVVLEWPKKDKKRLLHAVYRVGDLDRTIKCYTECFGMKLLRKRDVPEEKYTNAFLGFGPEDTNFALELTYNYGVDKYDIGAGFGHFAIATEDVYKLAEKIKSSCCCKITREPGPVKGGSTVIAFAQDPDGYMFELIQRGPTPEPLCQVMLRVGDLDRSIKFYEKALGMKLLRKKDVPDYKYTIAMLGYADEDKTTVIELTYNYGVTEYTKGNAYAQVAIGTEDVYKSAEAVELVTKELGGKILRQPGPLPGLNTKIASFLDPDGWKVVWLLSSCSTVFYYC

>ATGLYI-1

MAANMMRPAFAYTVVYVKDVAKSVEFYSRAFGHNVRRLDESHRWGELESGQTTIAFTPLHQHETDDLTGKVQATQSARERAPIEVCFCYPDVDAAFKRAVENGAEAVSKPEDKEWGQKVGYVRDIDGIVVRIGSHVK

>ATGLYI-2

MSSYSIASAISRISPLIRFVKPYSTGFSFITCACNSTRRPKRFDQLCVFSMASEARESPANNPGLSTNRDEATKGYIMQQTMFRIKDPKASLDFYSRVLGMSLLKRLDFSEMKFSLYFLGYEDTTTAPTDPTERTVWTFGQPATIELTHNWGTESDPEFKGYHNGNSEPRGFGHIGVTVDDVHKACERFEELGVEFAKKPNDGKMKNIAFIKDPDGYWIEIFDLKTIGTTTVNAA

>ATGLYI-3

MNEIASASMLRLCQCFISICNVHFVSMRAAESSFLLSRNMAEASDLLEWPKKDNRRFLHVVYRVGDLDRTIEFYTEVFGMKLLRKRDIPEEKYSNAFLGFGPETSNFVVELTYNYGVSSYDIGTGFGHFAISTQDVSKLVENVRAKGGNVTREPGPVKGGGSVIAFVKDPDGYTFELIQRGPTPEPFCQVMLRVGDLDRAIKFYEKALGMRLLRKIERPEYKYTIGMMGYAEEYESIVLELTYNYDVTEYTKGNAYAQIAIGTDDVYKSGEVIKIVNQELGGKITREAGPLPGLGTKIVSFLDPDGWKTVLVDNKDFLKELE

>ATGLYI-4

MKEDAGNPLHLTSLNHVSVLCRSVDESMNFYQKVLGFIPIRRPESLNFEGAWLFGHGIGIHLLCAPEPEKLPKKTAINPKDNHISFQCESMGVVEKKLEEMGIDYVRALVEEGGIQVDQLFFHDPDGFMIEICNCDSLPVVPLVGEMARSCSRVKLHQMVQPQPQTQIHQVVYP

>ATGLYI-5

MATASFRWILQLHRDVPKAARFYEKGLDFSVNVVTLRWAELQSGPLKLALMQAPSEHVMSEKGYSSLLSFTVADINTTISKLMELGAELDGSIKYEVHGKVASVRCLDGHVLGLYEPS

>ATGLYI-6

MVRIIPMAASSIRPSLACFSDSPRFPISLLSRNLSRTLHVPQSQLFGLTSHKLLRRSVNCLGVAESGKAAQATTQDDLLTWVKNDKRRMLHVVYRVGDMDRTIKFYTECLGMKLLRKRDIPEEKYTNAFLGYGPEDSHFVIELTYNYGVDKYDIGAGFGHFGIAVDDVAKTVELVKAKGGKVSREPGPVKGGKTVIAFIEDPDGYKFELLERGPTPEPLCQVMLRVGDLDRAIKFYEKAFGMELLRTRDNPEYKYTIAMMGYGPEDKFPVLELTYNYGVTEYDKGNAYAQIAIGTDDVYKTAEAIKLFGGKITREPGPLPGISTKITACLDPDGWKSVFVDNIDFLKELE

>ATGLYI-7

MKDETGNPLHIKSLNHISLLCRSVEESISFYQNVLGFLPIRRPDSFDFDGAWLFGHGIGIHLLQSPEPEKLLKKTEINPKDNHISFQCESMEAVEKKLKEMEIEYVRAVVEEGGIQVDQLFFHDPDAFMIEICNCDSLPVIPLAGEMARSCSRLNIRQLVQPTQIHP

>ATGLYI-8

MEEKKKKGDDELNSKPPLMALNHVSRLCKDVKKSLEFYTKVLGFVEIERPASFDFDGAWLFNYGVGIHLVQAKDQDKLPSDTDHLDPMDNHISFQCEDMEALEKRLKEVKVKYIKRTVGDEKDAAIDQLFFNDPDGFMVEICNCENLELVPCHSADAIRLPEDRHAPPVALPDSSNRRMPQPNS

>ATGLYI-9

MASLGHIARESSDITRLAQFYKEVFGFEEIESPDFGDLQVVWLNLPGAFAMHIIQRNPSTNLPEGPYSATSAVKDPSHLPMGHHICFSVPNFDSFLHSLKEKGIETFQKSLPDGKVKQVFFFDPDGNGLEVASRS

>ATGLYI-10

MATASFRWILQLHRDVPKAARFYAQGLDFSVNVVTLRWAELHSGPIKLALMQSPSNHVAEKGYSSLLSFTVTDINTTVTKLMALGAELDGTIKYEIHGKVAAMKCPDGYMLGLYEAA

>ATGLYI-11

MASIFRPSSASLDLRPKVICTNLSTKERFEFQKKSVRKERINVRFYSLKAKAQGSSIEGISVVQEKELNNKTDYGVVGVHHVGLLCENLERSLEFYQNILGLEINEARPHDKLPYRGAWLWVGSEMIHLMELPNPDPLTGRPEHGGRDRHACIAIRDVSNLKEILDKAGIAYTMSKSGRPAIFTRDPDANALEFTQV

>GmGLYI-1

MSSSIRPSLSSFMLPSLASCNPSQKLSLFRLGSGIRQFHKFGLKASRFLRHDDKCMRVMAFGNMSTAATQENVLDWVKHDKRRMLHVVYRVGDLDKSIKFYRECLGMKLLRKRDMQEQKYTNAFLGYGPEDAHFVVELTYSNSYGIEKYDIGDGFGHFGIAIDDISRIVELVRAKGGKITREPSPVKGGNTTIAYIEDPDGYQFELLERVPSPEPLCKVMLRVGDLDRSIKFYEKAFGMELLRTQDDPESKSTIGILGYGPEEKNTVLELTYNYGVTNYDKGDAYAQITIDTDDVYKTAEAIKLAGGKITREPGPIPVMKTKITSCVDPDGWKTVFVDNVDFRRELE

>GmGLYI-2

MKMEIEEVGNCEALPLLSLNHVSLLCRSVWVSMRFYEDVLGFVPIKRPSSFKFTGAWFYNYGIGIHLIENPNIDEFDTCVNEERPINPKDNHISFQCTDVELVKKRLEERGMRYVTAVVEEGGIQVDQVFFHDPDGYMIELCNCENIPIIPISSCSFKPRGHSFKKAAPNKCGFMENVMMESLSTDMINFSF

>GmGLYI-3

MSSSLMLPAASMLRPCTTSSSSCTSSRRLALFHLVSTGSIALPQAQLFGAKGPELLRVVEASAAEKLAQPEKDLFDWVKNDNRRFLHVVYRVGDLEKTIKFYTECLGMKLLRQRDIPEDRYSNAFLGYGPEDSNFTVELTYNYGVDNYDIGSGFGHFGVAVEDIYKRVDLVKAKGGKVTREPGPVKDGSAVIAFIEDPDGYKFELLERRPTSEPLCQVMLRVGDLDRAIAFYEKAVGMKLLRKRDNPEQKYTVAFMGYGPEDKNTVLELTYNYGVTNYDKGNGYAQIAIGTNDVYKTAEAIKLCGGKIIREPGPLPGINTKIVACLDPDGWKLAFVDNVDFLKELE

>GmGLYI-4

MVLVRVVPMASSSSIRPTLSSLRFLTPSSLSLSNPSSRISFSHLPSPSVSQSNSFGLKASRELRQHGNSTRIMASGDVSQSISAASPENVLEWVKQDKRRMLHVVYRVGDLDRTIKFYTECLGMKLLRKRDIPEEKYTNAFLGYGPEDSHFVIELTYNYGVDKYDIGTGFGHFGIAVDDVAKAVELIRAKGGKITREPGPVKGGRSVIAFIEDPDGYKFELIERGPTPEPLCQVMLRVGDLNRSIEFYEKAFGMELLRTRDNPEYKYTIAMLGYGPEDKSTVLELTYNYGVTEYDKGNAYAQIAVGTDDVYKTAEAIKLAGGKITREPGALPGINTKITACLDPDGWKSYITRSGRSVFCLLWHDT

>GmGLYI-5

LPQAQLFGAEKIAQPEKNLFDWVKNDNRRFLHVVYRVGDLEKTIKYALLRKRDIPEDRYSNAFLGYGPEESNFTVELTYNYGVDNYDIGSGFGHFGVAGRLITREPGPVKDGSAVIALIEDPDGYKFELLERRPTSEPLCQVMLRVGDIDRAAGMKLLRKRDNPEQKYTVAFMGYGPEYMNSVLELTYNYGVTNYDKGNGYAQIAIGTNDVYKTAEAIKLCGRKIIREPGPLPGINTKIVACLDPDGWKLAFVDNVDFLKELE

>GmGLYI-6

MKESVGNPLRLQSVNHISLICRSVEQSMDFYQNVLGFYPIRRPGSLDFDGAWLFGYGIGIHLLEAENPEKLPKKKEINPKDNHISFQCESMVAVEKKLKEMEIDYVRATVEEGGIQVDQLFFHDPDGFMIEICNCDSLPVIPLVGEVARSCSLVNLEKMQNQQQIQKMLQQL

>GmGLYI-7

MADLLEWSKQDKKRMLHVVYRVGDLDRTIKFYTECLGMKLLRQRDIPEEKYANAFLGFGPEESHFVVELTYNYGVTSYDIGDGFGHFAIATQDIYKLVEHIRAKGGNITREPGPVQGGTTVIAFVKDPDGYTFGLIQRPTVHDPFCQVMLRVGDLERSIKFYEKALGMKVVRKVDKPEYKYTIAMLGYGEEHETTVLELTYNYGVTEYSKGNAYAQIAIGTDDVYKSAEVVNQVIKEVGGKITRQPGPIPGLNTKTTSFLDPDGWKTVLVDNVDFLEELK

>GmGLYI-8

MVLVRLVPMASSSIRPALSTPSSFSLFSPSRRISFSHLPSPSVSQSNSFGLKASRVLRQYGNSTRIMASGDLSHSVAAASPENVLEWVKQDKRRMLHVVYRVGDLDRTIKFYTECLGMKLLRKRDIPEEKYTNAFLGYGPEDSHFVIELTYNYGVDKYDIGTGFGHFGIAVDDVAKAVELIRAKGGKITREPGPVKGGRSVIAFIEDPDGYKFELIERGPTPEPLCQVMLRVGDLNRSIEFYEKAFGMELLRTRDNPEYKYTIAMLGYGPEDKSTVLELTYNYGVTEYDKGNAYAQIAIGTDDVYKTAEAIKLAGGKITREPGPLPGINTKITACLDPDGWKSVFVDNVDFLKELE

>GmGLYI-9

MKESTMGNPLRLQSVNHISLICRSVEQSMDFYQNVLGFYPIRRPGSLDFDGAWLFGYGIGIHLLEAENPENLPKKKEINPKDNHISFQCESMEPVEKKLKEMEIDYVRATVEEGRIQVDQLFFHDPDDFMIEICNCDSLSR

>GmGLYI-10

MLLYKATRPQCRRLFWFVVLCLAIFTIFTEHLHSTLNMAEATQSNAELLEWPKKDKRRFLHVVYRVGDLDRTIKFYTECFGMKLLRKRDIPEEKYANAFLGFGPEQSHFVVELTYNYGVTSYDIGTGFGHFAIATPDVYKLVEDIRAKGGNITREPGPVKGGKSVIAFVKDPDGYAFELIQRSSTPEPLCQVMLRVGDLERSIKFYEKTLGLRVVKKTDRPEYKYTIAMLGYAEEHETTVLELTYNYGVTEYTKGNAYAQVAIGTDDVYKSAEVVNIVTQELGGKITRQPGPVPGLNTKITSFLDPDGWKTVLVDNQDFLKELE

>GmGLYI-11

MASSIRPSLSSFMLPSLRSCNPSEKLSLFHLGSGIRLYHKFGLKSSRLLRHDDNKCMRVMASGNMSTAATQENVLDWVKHDKRRMLHVVYRVGDLDKSIKFYRECLGMKLLRKRDMQEQRYTNAFLGYGPEDAHFVAELTYNYGIDKYDIGDGFGHFGLAVDDISRIVELVRAKGGKITREPSPVKGGNSTIAYIEDPDGYQFELSERVSSPEPLSKVMLRVGDLDRSIKFYEKAFGMELLRTQDDPESKSTIAILGYGPEEKNTVLELTYNYGVTDYDKGDAYAQITIGTDDVYKTAEAIKLAGGKITREPGPVPGIKTKITLCVDPDGWKTVFVDNVDFRRELE

>GmGLYI-12

KEGKGKEENPPPLLAMNHVSRLCRNVKESIDFYTKVLGFVLIERPQALDFEGAWLFNYGVGIHLCEDLEAMEKKLKEKNVKYMKRTLEREDGTTMDQIFFNDPDGFMVEI

>GmGLYI-13

MKMEIEEVGNCEALPLLSLNHVSLLCRSVWESMRFYEDVLGFVPIKRPSSFKFTGAWFYNYGIGIHLIENPNIDEFDTCVVEERPINPKDNHISFQCTDVELVKKRLEERGMRYVTAVVEEGGIQVDQVFFHDPDGYMIELCNCENIPIIPISSCSFKPRGHSFKKAAPNKCGFMENVMMESLSTDMINFSF

>GmGLYI-14

MKDSSSAMVHSMRRSFFNFCLTEKAQLDLYGHSNRINCDCSEPKESPSNNPGLHTTPDQATKAYFTQQTMFRIKDPKVSLDFYSRVLGTYLLKRLDFLEMKFSLYFMGYEDTTKAPSNPVERTVWTFSQKATMELTDNWGTENDPEFKGYHNGNSEPLGYGHIGIAVDDTYKACERFQNLGVEFVTKPDDGFFSQHYCFKHYFQVFFILELFSAGEIKGLAFIKDPDGYWIELFDLKILGGEQAAAHA

>GmGLYI-15

MTVTASLHRLSRLRFIAKPQPFLSPHSIPSHFSLTPKTKKANRFRFLSMAAEPKESPSNNPGLHTTPDEATKGYIMQQTMFRIKDPKVSLDFYSRVLGMSLLKRLDFPEMKFSLYFMGYENTAEAPSNPIDKVVWTFSQKATIELTHNWGTESDPEFKGYHNGNSEPRGFGHIGVTVDDTYKACERFQNLGVEFVKKPEDGKMKGIAFIKDPDGYWIEIFDRKTIGNVTQTAA

>GmGLYI-16

MAATASLHRLSRLRFIAKPQPFLSPHSTPSHFSLTPKTKKPNRFRFRFRSMAAEPKESPSNNPGLHTTPDEATKGYIMQQTMFRIKDPKVSLDFYSRVLGMSLLKRLDFPEMKFSLYFMGYEDTTEAPSNPIDKVVWTFSQKATIELTHNWGTESDPEFKGYHNGNSEPRGFGHIGITVDDTYKACERFQNLGVEFVKKPDDGKMKGIAFIKDPDGYWIEIFDRKTIGNVTQAPA

>GmGLYI-17

MEKMELAETPLPLLSLNHVSFVCKSVSESVKFYEDVLGFLLIKRPSSFKFEGAWLFNYGIGIHLLESEKVPVKKREINPKENHISFQCSDMKVIMQKLDAMKIEYVTAVVEEGGIKVDQLFFHDPDGYMIEICNCQNLPVLPISSCPLKQLGGEATFKINCFAEESMSMLMMDNFVMDMLKISI

>GmGLYI-18

MSCCSCSAMAFLLKAPSFLPPLNQKLNYTHKSFSPINLQSKFYHASVRNGRWNVPSMTIKAQAAVEGDVLLDEESICVNEESDYGVVCMHHVGILCENLERSLDFYQNVLGLKINEARPHNKLPYRGAWLWVGSEMIHLMELPNPDPLTGRPQHGGRDRHTCIAIRDVSKLKAIFDKAGIAYTLSHSGRPAIFTRDPDANALEFTQVDD

>GmGLYI-19

MANPLQLKSLNHISIVCASVEKSVDFYVNVLGFSPIKRPSSLDFNGAWLFNYGIGIHLLQSENPEGMPKTAPINPKDNHISFQCESIAAVEKRLQQMKIEYVKNRVEESGTYVDQLFFHDPDGMMIEICNCDNIPVVPLTEDKVWSCSRFNCNIQNHQQQIQQMIPM

>GmGLYI-20

MKENVGNPLHLKSVNHISLICTSVKESINFYQNLLGFFPIRRPGSFDFDGAWLFGYGIGIHLLQAEDPDNVPRKTKINPKDNHISFQCESMGAVEKKLGEMEIEYVHATVEEGGIKVDQLFFHDPDGFMIEICNCDSLPVIPLAASGNNNGMVRSCSRLNLQILQQIHQFLNQ

>GmGLYI-21

MAEATQSNAELLEWPKKDKRRFLHVVYRVGDLDRTIKFYTECFGMKLLRKRDIPEEKYANAFLGFGPEQSHFVVELTYNYGVTSYDIGTGFGHFAIATPDVYKLVEDIRAKGGNVTREPGPVKGGKSVIAFVKDPDGYAFELIQRPSTPEPLCQVMLRVGDLERSIKFYEKALGLRVVKKTDRPEYKYTIAMLGYAEEHETTVLELTYNYGVTEYTKGNAYAQVAIGTDDVYKSAEVVNIVTQELGGKITRQPGPIPGLNTKITAFLDPDGWKTVLVDNQDFLKELE

>GmGLYI-22

MGKMEPLPLLSLNHVSFVCKSVSESVKFYQDVLGFVLIKRPSSFKFEGAWLFNYGIGIHLLESEKVPVEKREINPKENHISFQCSDMKVIMQKLDAMKIEYVRAVVEEGGIKVDQLFFHDPDGYMIEICNCQNLPVLPISSCPLKQLAAGEATTLNINCFADESVSMLMMDNLVMDMLKISI

>GmGLYI-23

MNCCSAMASLLKSPSFLSPLNQKLNYVSFSPMTTNLQSKFCRASVRNGRWHVPSLTIKSQAAVEGDVLEKESVSINEESDYGVVCMHHVGILCENLERSLEFYQNVLGLKINEARPHDKLPYRGAWLWVGSEMIHLMELPNPDPLTGRAQHGGRDRHTCIAIRDVSKLKAIFDKAGIPYTLSHSGRPAIFARDPDANALEFTQVDG

>GmGLYI-24

MANPLQLKSLNHISIVCASVEKSVDFYVNVLGFSPIKRPSSLDFNGAWLFNYGIGIHLLQSEDPEGMPKLVPINPKDNHISFQHSSGGKRLQQMKIEYVKNRVEENGMMIEICNCDNIPVVPLPEDKVWSCSRFNCNIQNRQQQI

>MtGLYI-1

MGNETQTKTGFKLVGFKKFIRTNPKTDRFKVKRFHHVEFWCTDATNTALRFSQGLGMPIVAKSDLSTGNLIHASYLLRSGDLNFLFSAPYSPSISLSSPSSTASIPTFSASTCFSFCASHGLAVRAIAIEVDDAELAFTVSVNHGALPSSPPIVLENGVKLAEVHLFGVDVVLRYVSYNNPNLLFLPGFESLLNESSNSSLDFGIRRLDHANANVPELASAVKYIKQFTGFHEFAEFTTEDVGTSESGLNNVVLASNDETVLLPICEPIYGTKRKSPIETYLEHNEGAGFQHLALASEDIFRTLREMRKKSGVGGFEFMAPPPVTYYRNLKNRVVDVLSDEQIKECEELGILVDRDDQGTILQIFTKPVGDRPTVLIEIIQRVGCMLKDEEEKEYQRGGCGGFGKGNFSELFKSIEEYEKTLETRRTA

>MtGLYI-2

MKETVAFYEKVLEFISIVRPGSFDFGGAWLFGHGIGIHLLLAEDPEKIPRKNEINTKDKHISFQCDESMDAVEKYLKDMKIGLKRAMVEENGIQVDQLFFHDPDGFMIEICNCDSLPVIPLAGGMVTLCPRLNFESMPQQIDQVAKQI

>MtGLYI-3

MPLPLLSLNHVSFVCRSLQESVKFYENVLGFVLIKRPSSFKFQGAWLFNYGIGIHLLETESDKVPVKRGEINTKENHISFQCSDMKLIMKNLDEMNIEYKTAVVEDGGIKVDQLFFHDPDGYMIEMCNCQNLPVLPISTCPLKQPTNQAPVPFYGEGKNCHAEEALLMMEILVIDLLRISI

>MtGLYI-4

MAEAAQPNAELLEWAKKDKRRFLHAVYRVGDLDRTIKFYTEAFGMKLLRKRDVPEEKYANAFLGFGPETSNFVVELTYNYGVTSYDIGTGFGHFAIATPDVYKFVENARAKGGKVTREPGPVSGGTSVIAFVADPDGYLFEILQRASTPEPLCQVMLRVGDLERSIKFYEKALGLKLARTIDRPQYKYTLAMLGYAEEHETIVLELTYNYGVTEYTKGNAYAQVAVGTDDVYKSAELVNLATQEFGGKITRQPGPIPGLNTKITSFLDPDGWKTVLVDNQDFLKELE

>MtGLYI-5

MDAIVGNPLRLKSVNHISLICRSVDVTVAFYENVLGFVSIVRPGSFNFEGAWLFGHGIGIHLLKAEDPEKIPRKKEINTKDNHISFQCDGSIDAVEKYLNDKKIVCKRALVEENGIQVDQLFFHDPDGFMIEICNCDSLPVIPLAGEIVNSCSRINLETMPQKIHQPVEKI

>MtGLYI-6

MKEIVGNPLRLKSVNHISLICKSVNESVSFYEKVLGFISIVRPGSFDFEGAWLFGYGIGIHLLQAEDPENIPRKNEINPKDNHISFQCDESMDTVEKYLNDKKIGCKRAMVEENGIQVDQLFFHDPDGFMIEICNCDSLPVIPLAGEMVRSCSRLNLEIMPQQIHQVVKQI

>MtGLYI-7

MHAIAMDVYGKRSLLTLMDKEKTEQQESPNIHLHVQIHSHREANEQDIQFSPPRPSTTFPQSPWTLSSLPPPSPSLLYHCIASLHRHEGNIYSIAVSKGFIFTGSNSSRIRVWKQPDCMDKGYLKSNSGEIRTILAYNNMVFSSHKDHKIRIWNFNVSENFKSKKVATLPKRSKNSFLNFSRTKNNNSHNHKHKDLVSCMAYYHSEGLLYTGSHDRTVKAWRISDRNCVDSFLAHEDHVNAILVNQDDGCVFTCSSDGSVKIWRRVYTENSHTLTMTLKFQHSPVNTLALSSSFNHCFLYSGSSDGMINFWEKERLCYRFNHGGFLQGHRFAVLCVETVGNMVFSGSEDTTIRVWRREEDSCYHECLMVLDGHRGPVRCLAACLEMEKVVVGFLVYSASLDQTFKVWRIKVFSEDENVCLDGDNNKCDGRVKKIREYDMSPVLSPSWVEKKLQGGNKETDKGRSVSFICITMISSLMLPSATTLRPCCSCSITPSSSSSSSRRIALFHLLTTGGIALPQSQLLGGKGSDLFQIAEANAAVNLAQPDQNLFNWVQNDNRRFLHVVYKVGDLDKTIKFYTECLGMKLLRKRDIPEDKYSNAFLGYGPEDSSFTVELTYNYGVDNYDIGTGFGHFGIIAEDVSKTVDIVKAKGGKVTREPGSVKGGSIVTASVEDPSGYRFELLERRPTREPLCKVMLRVGDLDRVIAFYEKAVGMKLLHKIDNPEEKYTVAKLGYGPEANGPVLQLTYNYGVTNYDKGNGYAQIAIGTDDVYKTAEAIKSCGGKIIREPGPLPGINTKIVVCLDPDGWKLVFVDNVDFLKELE

>MtGLYI-8

MKESVGNPLHLKSVNHISLICRSVEESIDFYQNVLGFFPIRRPGSFDFDGAWLFGYGIGIHLLEAENPETLPRKKEINPKDNHISFQCESMGAVEKKLKEMEINYVRARVEEGGIEVDQLFFHDPDGFMIEICNCDSLPVIPLVGEVARSCSRLNLHIMQNQNQNQQNQIHKIVK

>MtGLYI-9

MCVKFDRFYTECLGMKLLRKRDIPEDKYSNAFLGYGPEDSSFTVELTYNYGMDNYDIGTGFGHFGIIAEDVSKTVDIVKAKGGKVTREPGSVIGGSIVTASVEDPSGYRFKLLERRTTREPLCKVMLRVGDLDRVIAFYEKAVGMKLLHKIDNPEEKYTVAKLGYGPVLELTYNYGVTNYDKGNGYAQIAIGTDDVYKTAEAIKSCGGKVIREPGPLPGINTKIVVCLDPDGWKLVWHFSIFSLYLM

>MtGLYI-10.1

MMSIATSNFLSRFRFIAKHQSLPIRSPVSIPFHFSLKKQPIRRFRFFSMAASESKESPANNPGLHATVDEATKGYFMQQTMFRIKDPKVSLDFYSRVLGMSLLKRLDFPEMKFSLYFMGYEDTSEAPSNSVDRTVWTFAQKATIELTHNWGTESDPEFKGYHNGNSDPRGFGHIGITVDDTYKACERFQNLGVEFVKKPEDGKMKGIAFIKDPDGYWIEIFDRKTIGNVTGSAA

>MtGLYI-11.1

MASKLSPEFAYTVLYVKDVAESVAFYSKAFGYSVRRLDESHRWGELESGHTTIAFTPIHQHETDDLTGVVHTTRSNKERPPVEVCFVYTDVDAAYKRAVENGAVPVSEPEMKEWGQKVGYVRDIDGIVIRMGNHVKPAKLD

>MtGLYI-12

MGNPLQLKSLNHISLVCRSLDKSVDFYVNVLGFFPIKRPTSLAFNGAWLFNYGIGIHLLQSDDPESMTKNVHINPKDNHISFQCESMAAVENKLQQMKIEYVKNLVEENGIYVDQLFFHDPDGTMIEICNCDNIPIVPLSENSTIWSCSRFNCNIQNQQQQIQQMISM

>MtGLYI-13.1

MASLLKVSSFISPLHHKLNYVSFSPKFNHVSVRNERWNAPSITVKAQTAVEGDVINNESLSSNEQSDYGVVSVHHVGILCENLERSLDFYQNVLGLKINEARPHDKLPYRGTWLWVGSEMIHLMELPNPDPLTGRPQHGGRDRHTCIAIRDVSKLKAILDKAGVPYTLSRSGRPAIFTRDPDANALEFTQIDD

>MtGLYI-14.1

MAEIDLEWPKKDNRRLLHVVYRVGDLERTIKFYTEALGMKLLRQRDVPEEKYANAFVGFGDEHSHFAVELTYNYGVTSYDVGDGFGHFAIATQDVYKLVEHIRAKGGNITREAGPVQGGTTVIAFVKDPDGYTFALVQRPIVHDPFCQISLRVGDLERAIKFYEKALGLKVVRKVDNPENKYTIAILGYKEEDDATVLELTYNYGVTEYSKGTAYAQIAIGTDDVYKSADVVNLVTQELGGEITLQPGPIPGLNTKVTSFLDPDGWKTALVDNEDFLKELE

>MtGLYI-15.1

MSGFCVGLSLPTDIPHSLSHLVVMAEIDLEWPKKDNRRLLHVVYRVGDLERTIKFYTEALGMKLLRQRDVPEEKYANAFLGFGDEQSHFVVELTYNYGVTSYDVGDGFGHFAIATQDVYKLVEHIRAKGGNITREAGPVQGGTTVIAFVKDPDGYTFALVQRPIVHDPFCQISLRVGDLERAIKFYEKALGLKVVRKVDNPENKYTIAILGYKEEDDATVLELTYNYGVTEYSKGTAYAQIAVGTDDVYKSADVVNLVTQELGGKITRQPGPIPGLNTKVVSFLDPDGWKTVLVDNEDFLKELE

>MtGLYI-16.1

MAGVCLNHISRESNDINRLAKFYQEIFGFEEVESPKFGEFKVVWLRVPSSSLYLHLIERNPSNNLPEGPWSATSPVKDPSHLPRGHHLCFSVSNFQSFLQTLKDKGIETFEKSLPNGKIKQVFFFDPDGNGLEVASKEDS

>MtGLYI-17.1

MAAAEGVSLNHIARESTDVKRLSKFYQEMFGFEEVETPDFGELKIIWLRLPSSSLLIHLIQHSNGELAPSSSIPVKDPSHIRLGHHLCFSISNLHSFHNTLKDKGIETFETTNGNIKRVFFYDPDGNELEVFASIEDSS

>MtGLYI-18.1

MAASFRWLLQLHKDVPKAARFYSEGLDFTVNVCTLRWAELQSGPLKLALMHSPIDQSTQKGYSSLLSFTVTDINSTVTKLMALGAELDGPIKYEVHGKVAAMRCIDGHLLGLYEPV

>MtGLYI-19.1

MQLLDSLREQELKMEIEEVCEAQALPLLSLNHVSLLCRSVLESMQFYEDVLGFVPIKRPSSFKFTGAWFYNYGIGIHLIQNPDIDEFDTYMNESRPINPKDNHISFQCTDVELVKKRLEEKGMRYVTALVEDEGIKVDQVFFHDPDGYMIELCNCENIPIIPISSCTASFKPRSHSFKRSTSNFKCGGFMQNVMMQSLSMDMMNFAF

>MtGLYI-20.1

MANETHNQTGFKLVGCKNFIRTNPKTDRFKVKHFHHVEFWCTDATNTAHRFSHGLGMPIVAKSDLSTGNLTHASYLLRSGDLNFLFTAAYSPSISLSSPSSTASIPTFSPSTCFSFSNSHGLNVRALAVEVEDAELAYTVSVSYGALPSSPPVVLENGVKLAEVRLFGDVVLRYVSYNNPNQNQNLLFLPGFETLSGESSNSSLDFGIRQLDHANGNVPELSSALKYIKQFTGFHDFAEFTAEDVESGLNAVALANNDETVLLPLCEPVYGTKRKSTIETYLEHNEGAGFQHLALASEDIFKTLREMRKRSGVGGFEFMPSPPVTYYRNLKNRVGDVLSDEQIKECEELGILVDRDDQGTLLQIFTKPIGDRPTIFLEIIQRVGCMLKDEEGKEYQKGGCGGFGKGNFSELFKSIEEYEKTLETRRTA

>MtGLYI-21.1

MAIETETQTQTQTGFKLVGFKNFVRANPKSDRFNVKRFHHVEFWCTDATNTARRFSHGLGMPIVAKSDLSTGNLTHASYLLRSGDLNFLFSAAYSPSISLSSPSSTAAIPTFSASTCFSFSASHGLAVRAVAVEVEDAEVAFTTSVNLGAIPSSPPVILENNVKLAEVHLYGDVVLRYVSYNDLNPNQNPNLFFLPGFERVSDESSNSSLDFGIRRLDHAVGNVPELSSAVKYVKQFTGFHEFAEFTAEDVGTSESGLNSVVLANNEETVLLPMNEPVYGTKRKSQIETYLEHNEGAGLQHLALMSADIFRTLREMRKRSGVGGFEFMPSPPVTYYRNLKNRVGDVLSDEQIKECEELGILVDRDDQGTLLQIFTKPIGDRPTIFIEIIQRVGCMLKDEEGKEYQKGGCGGFGKGNFSELFKSIEEYEKTLETRRTA

>MtGLYI-22.1

MASSSIRPSLSSLNKLPSFSSRNLSQRFSLFHLRNGVRLLPQNFGLKASRLLRHDSGSMRVMASRSMSQSVTQENALDWVKWDKRRMLHVVYRVGDFDKSIKFYTECLGMKVLRKRDMTEEKYTNAFLGYGPEDAHFAIELTYNYGIETYDIGTGFGHYGIAMDDISRVVDIVRAKGGIITREPGPVKGGDSTVAVIEDPDGYKFELLERAPSPEPLCKVMLRVGDLDRSIKFYEKVVGMELLRKQDDPESKCTVAIMGYGPEEKTTVLELTYNYGITKYDKGDAYAQIAIGTDDVYKTAEAIKLAGGKITREAGPVPGYRTKITSCVDPDGWKTVFVDNHDFHKELE

>MtGLYI-23.1

MGIQEIGSYEAPLPLLSLNHVSILCRSVLDSMRFYEEILGFGLIKRPSSFKFNGAWLYNYGFGIHLLENPNYDEFDTPMSESRPINPKDNHISFQCTDVGLVKMRLEDMGMKYVTALVEDEGIKVEQVFFHDPDGYMIELCNCENIPIVPISSASGSFKARGQSFKKTVSNKCGFMENVMMRSLTKDMMNFAF

>MtGLYI-24.1

MVRVIPIASSSILPTLSLFNRTPRISFSHFSTAVPQSHNFGLKACRLFKQNGNSLKVMSSGNVSSSVTAASPENVLEWVKQDKRRMLHVVYRVGDLDRTIKFYTECLGMKLLRKRDIPEERYTNAFLGYGPEDSHFVIELTYNYGVDKYDIGTAFGHFGIAVDDITKTVELIRAKGGKITREPGPVKGGKTVIAFVEDPDGYKFELLERGPTPEPLCQVMLRVGDLNRSIEFYEKAFGMELLRTRDNPDNKYTIAMLGYGPEDKSTVLELTYNYGVTEYDKGNAYAQIAIGTDDVYKTAEAIKLSTGKLTREPGPLPGINTKITACLDPDGWKTVFVDNIDFLKELE

>MtGLYI-25.1

MQKQEVKEEERNSNKKEEKNEKEGDEGTKESNQTPLMALNHISRLCRDVKESIDFYTKVLGFVLIERPQVLDFEGAWLFNYGVGIHLVQSKEEQKLPSPDAQHDLDPQDNHISFQCEDVKGMEKKLKEMKVKYKKRNLEAEDGTTMDQIFFNDPDGFMVEICNCENLKLTPADSQGKIKIPMDRHTPPVETNQNEHDNVK

>MtGLYI-26.1

VAKTVNIVKEKWGKVMRELEPVKDGSTVTAFIEDPSGDRFELLGRRLTREPLCKVMLQADNLDCVIAFYEKAVGMKLLHKIVNPK

>MtGLYI-27.1

MGTIRALGYVGFTATNIDRWKDFAPGVLGLQLSETWPDGTLVLRADAYQRRIFIHPGRVDEIRYIGWEVYDADSLEKLKSQLTGKRVPFVDLNEDETAHRAVIDGLKFLDTDGLHIEAFYGASQSKHEPFVSPVGQGPFVTGEQGLGHIVVHPENYSAAVAFYKDVLDFKISDYCTINTLGARDGHATFMHVNPRHHSLALANFPIGQRLNHIMLELETIDDVGCAYERALAAGAHILLDLGRHTNDDVFSFYVMTPSGWSVEIGCGGRRIDDTTWHVSHHTRPSSWGHNLDSTSRMVKVGNLDIHYHDSGSGDQVVVLLHGGGPGASSWSNFQRNIGPLSEHFRVLAIDMPHFGKSTKPEGRYLDRPWYAEVVGATLDTLNISKAHFVGNSLGGSVSMVLSVERPEIVDRLVLMGTMGSLPVFAPLPPEGAKHIIEYYHGEGPTREKLEAFLRSMIYDQNLITTDFIEERFIASTTPELLFVAQQPKLNTHFTQWQTADQVKHKSLLLYGRDDRVVPWDTSLLLLRLMPNADLHIFSRCGHWAQWERADEFNSVVANFL

>MtGLYI-28.1

MLLGSRTDIRIAGESAEFGFTEIRHGLGGPAAIISRLRDQIPYTSLMWLAFGQHINAHEAHRIGLVNELVPDDQVLDRAMEVARAIAEVPPLAIRAEKQSLLRTQHQPFKEAVQYGTALFSMIQMSADAREGVQAFVEKRRPNFRMDRRREPAGRWRAGRFNAGSFMSRVVVVTGAASGNGLAIASRFLDHGDRVVAVDVSTDGLGARFQKEWRPYEERVIALTKDVSCQTDIDASRASQDLSFRVRPSTRGEGARGIPCMPEQCEGVVVNIASIATLVANPGRTSYATSKGALLQRTRSIAADYAHAGIRCNALCPGLIETAMTQWRRGGSEQRNQVLAKIPQNEVGTVDDVASAVMFISDPQSRYFNGAALVIKADHAAYVVSSLKEALTFWVDGMGATLEGRFKAGGPMLANVTGAVGADVSIALIEIAGQRLELLEYQGVTPNPGATLRPYDAGAMHLALNVDDVHAALRHVAQYGYRAQGVPQKAPTGSTAMYVVGPDGATIEFRQPEVA

>MtGLYI-29.1

MKKIATVLFSALMLASTCASAQALCKAGKIDKIETDTTGNLLVTINDGVYSFSAKEVYSIIYQAYSENRNLFIYGNNCANGSPATRFAIPPPRAGVPILRDMAPACPRPVHPSAIPVPVPMKPAGLPLRISRRPAFGASTASSRRSRRAGCPASLRRRPSGPGHEQTPGCRISPCADRTHPSRRRPMIDHVYISVTDIEKSLAFYAEALKPLGWRIFGNYDSASGPESVPDLYGIGDDVYGKGAGVGSSIWLRKRHPGETGLYVGIVCDTNELVDAAYAAAIKAGGIDEGKPADRTYFAPGYYAANVADFDGNRLEFVHKAWNPKRHA
