# Supplementary material for: From methylglyoxal to pyruvate: a genome-wide study for the identification of glyoxalases and D-lactate dehydrogenases in Sorghum bicolor
Source: BMC Genomics. 2020 Feb 10;21:145. doi: 10.1186/s12864-020-6547-7 (PMC7011430; doi:10.1186/s12864-020-6547-7)
Supplement: Supplementary file 10 — Additional file 10. Glyoxalase II protein sequences from various species used for the phylogenetic analysis. [file 12864_2020_6547_MOESM10_ESM.docx]

>SorbicGlyII-1

MASSAAAPSGAPPAGKRPASSGREGDQMVITPLGAGSEVGRSCVHMTFKGRTVLFDCGIHPAYSGMAALPYFDEIDPSTIDVLLITHFHLDHAASLPYFLEKTTFKGRVFMTHATKAIYRLLLSDYVKVSKVSVEDMLYDENDIARSMEKIEVIDFHQTLEVNGIRFWCYTAGHVLGAAMFMVDIAGVRILYTGDYSREEDRHLRAAELPQFSPDICIIESTYGVQQHQPRIVREKRFTEVIHNTVSQGGRVLIPAFALGRAQELLLILDEYWSKHPELHKIPIYYASPLAKRCMAVYQTYINSMNERIRNQFAQSNPFHFKHIESLNSIDNFHDVGPSVVMASPGGLQSGLSRQLFDKWCTDKKNACVIPGYVVEGTLAKTIINEPREVTLANGLTAPLHMSVHYISFSAHADFPQTSNFLDELRPPNIILVHGEANEMSRLKQKLKTQFDGTNIVSPKNCQSVEMYFTCEKMAKTIGRLAEKVPEGGESSGGLLVKKGFTYQIMAPEDLRVFTQLSTANITQRIAVPYSGSFEVIKYRLKQIYESVESATEESDVPALIVHERVTVRLDSESYVTLQWSSDPISDMVSDSVVAMILNIGREGPKVVPVEEAVKTKEETEKVAQKVVYALMTSLFGDVKVTAEGKFVISVDGNVAHLDGMSGDVKCENATLKERIKTAFRRIQSAVRPIPLSAS*

>SorbicGlyII-2

MGTSVQVTPLSGAYGEGPLCYLLAVDGFRFLLDCGWTDLCDTSQLQPLAKVAPTVDAVLLSHPDMMHLGALPYAMKHLGLSAPVYATEPVFRLGLLTMYDHFLSRWQVSDFDLFTLDDVDAAFQNVVRLKYSQNYLLNDKGEGIVIAPHVAGHLLGGTVWKITKDGEDVVYAVDFNHRKERHLNGTVLGSFVRPAVLITDAYNALNNQGYRKKQDQDFIDSLIKVLATGGSVLLPVDTAGRVLELLLLLDTYWDERRLQYPIYFLTNVSTSTVDYVKSFLEWMRDQIAKSFESNRANAFLLKKVMLIINKEELEKLGDAPKVVLASMASLEVGFSHDIFVEMANEARNLVLFTEKGQFGTLARMLQVDPPPKAVKVTMSKRIPLVGDELKAYEEEQERIKKEKALKASLVKEEELKASLGSNAKASDPMVIDASSSRKSANAGSHFGGNTDILIDGFVPPSTSVAPMFPFFENTAEWDDFGEVINPDDYMMKQEEMDNTLMLGPGDGLDGKIDDGSARLLLDSTPSKVISNEMTVQVKCSLVYMDFEGRSDGRSVKSVIAHVAPLKLVLVHGSAEATEHLKMHCTKNLDLHVHAPQIEETIDVTSDLCAYKVQLSEKLMSNIISKKLGEHEIAWVDAEVGKEDEKLILLPPSSTPPPHKPVLVGDLKLSDFKQFLENKGWQVEFAGGALRCGEYIMVRKIGDSSQKGSTGSQQIVIEGPLCEDYYKIRELLYSQFYLL*

>SorbicGlyII-3

MKIIPVPCLEDNYAYLIVDESTKKAAAVDPVEPEKVIKAAGEVGAYVDCVLTTHHHWDHAGGNEKMRLQVPGIKIFGGSLDNVKGCTDQVENGTKLSLGKDIEILCLHTPCHTKGHISYYVTSKEGEDPAVFTGDTLFIAGCGKFFEGTAEQMYQSLIVTLGSLPKSTRVYCGHEYTVKNLKFILTVEPENEKTKQKLEWAEKQRQANQPTVPSTIGDEFEINTFMRVDLPEIQAKFGANSPVEALREVRKTKDNWKG*

>SorbicGlyII-4

MRMLSKACSIVASSLPRCSSSAAPTMRGQPSLLPSVRKQWPGKPLLYGIGTLLVMPLRTLYGVGRVFGAGRFLCNMTSVSSSLQIELVPCLRDNYAYILHDVDTGTVGVVDPSEAMPIINALEKRNQNLTYILNTHHHYDHTGGNLELKAKYGAKVIGSEKDKDRIPGIDITLKEGDTWMFAGHQVLVLETPGHTSGHVCYYFAGSGAIFTGDTLFNLSCGKLFEGTPQQMYSSLQKITALPDDTKVYCGHEYTLSNSKFALSVEPGNKALQEYAANAAELRNKNIPTVPTTIGREKECNPFLRTSNPEIKSTLSIPDHFDEDRVLEVVRRAKDNF*

>SorbicGlyII-5

MVLPLRLIPRLASAARLTPSASCAPRLVLRRAPLLPVALAMASAYSAGSGADRRLLFRQLFEKESSTYTYLLADVADPDKPAVLIDPVDRTVDRDLNLIKELGLKLVYAMNTHVHADHVTGTGLIKTKLPGVKSVISKASGAKADHFVDHGDKIHFGNLFLEVRATPGHTSGCVTYVTGDADGQPSPRMAFTGDALIIRACGRTDFQGGSSDLLYQSVHSQIFTLPKDTLLYPAHDYKGFTVSTVEEEVAYNARLTKDKETFKTIMSNLNLSYPKMMDVAVPANLVCGIQDPPPKI*

>SorbicGlyII-6

MALLMSLGAAVYSYFSTATAPPRPRRCILSFRAAASPAALDRRRRPQNVAGDFFVDQRCIDCQTCRWMAPQVFKRVDGKAAVAAQPSSEEERTKALQALLSCPTSSIHTEKPPKDILQVQNMFPLPIDDKLLPGVYLCGYNSEDSYGATSYLVIHPQGNILIDSPRYTSKLANNIEKLGGARYMFLTHIDDVADHRKWAEQLKCERIIHMGDVEEATADVEWKLEGNGPWNIGTDFEFIHTPGHTPGSVCLYYKPPKVLFTGDHVAKSEESDDLNLFLMYSKQSVSLQLESIRKLLEVEFEWLLPGHGYRIRYKDVQAKNAAMESLLANYLS*

>OsGlyII-1

MVALLRSCRRLIPHLSACAAAAPSSSSSCAPRARPISRGLRLLPVVLAMAGYSSGSAAEGRRLLFRQLFEKESSTYTYLLADVGDPEKPAVLIDPVDRTVDRDLNLIKELGLKLVYAMNTHVHADHVTGTGLIKTKLPGVKSVIAKVSKAKADHFIEHGDKIYFGNLFLEVRSTPGHTAGCVTYVTGEGDDQPSPRMAFTGDALLIRACGRTDFQGGSSDELYESVHSQIFTLPKDTLLYPGHDYKGFTVSTVEEEVAYNARLTKDKETFKKIMDNLNLAYPKMIDVAVPANLLCGIQDPPPSKV*

>OsGlyII-2

MKIIPVACLEDNYAYLIVDESTKSAAAVDPVEPEKVLAAAAEVGVRIDCVLTTHHHWDHAGGNEKMAQSVPGIKVYGGSLDNVKGCTDQVENGTKLSLGKDIEILCLHTPCHTKGHISYYVTSKEEEDPAVFTGDTLFIAGCGRFFEGTAEQMYQSLCVTLGSLPKPTQVYCGHEYTVKNLKFILTVEPDNEKVKQKLEWAQKQREANQPTIPSTIGEEFETNTFMRVDLPEIQAKFGAKSPVEALREVRKTKDNWKS*

>OsGlyII-3

MRMLSKACSLVASSLPRCSSSAAPTIRGQPSLLPSVRKEWLGKPLLYGIGTLLVMPLRTLHGVGRMFGAGRFLCNMTSVSSSLQIELVPCLQDNYAYILHDVDTGTVGVVDPSEATPIINALEKRNQNLTYILNTHHHYDHTGGNLELKAKYGAKVIGSAKDRDRIPGIDITLSEGDTWMFAGHQVLVMETPGHTSGHVCYHFPGSGAIFTGDTLFSLSCGKLFEGTPQQMYSSLQKIIALPDETRVYCGHEYTLSNSKFALSIEPGNKDLQEYAANAADLRKRNTPTVPTTIGREKQCNPFLRTSSPEIKNTLSIPDHFDDARVLEVVRRAKDNF*

>ATGLX2-1

MPVISKASSTTTNSSIPSCSRIGGQLCVWPGLRQLCLRKSLLYGVMWLLSMPLKTLRGARKTLKITHFCSISNMPSSLKIELVPCSKDNYAYLLHDEDTGTVGVVDPSEAAPVIEALSRKNWNLTYILNTHHHDDHIGGNAELKERYGAKVIGSAVDKDRIPGIDILLKDSDKWMFAGHEVRILDTPGHTQGHISFYFPGSATIFTGDLIYSLSCGTLSEGTPEQMLSSLQKIVSLPDDTNIYCGRENTAGNLKFALSVEPKNETLQSYATRVAHLRSQGLPSIPTTVKVEKACNPFLRISSKDIRKSLSIPDSATEAEALRRIQRARDRF*

>ATGLX2-2

MKIFHVPCLQDNYSYLIIDESTGDAAVVDPVDPEKVIASAEKHQAKIKFVLTTHHHWDHAGGNEKIKQLVPDIKVYGGSLDKVKGCTDAVDNGDKLTLGQDINILALHTPCHTKGHISYYVNGKEGENPAVFTGDTLFVAGCGKFFEGTAEQMYQSLCVTLAALPKPTQVYCGHEYTVKNLEFALTVEPNNGKIQQKLAWARQQRQADLPTIPSTLEEELETNPFMRVDKPEIQEKLGCKSPIDTMREVRNKKDQWRG*

>ATGLX2-3

MVMTHFSRLRQLLLLQPKFLSSQPRPLRSPPPTFLRSVMGSSSSFSSSSSKLLFRQLFENESSTFTYLLADVSHPDKPALLIDPVDKTVDRDLKLIDELGLKLIYAMNTHVHADHVTGTGLLKTKLPGVKSVISKASGSKADLFLEPGDKVSIGDIYLEVRATPGHTAGCVTYVTGEGADQPQPRMAFTGDAVLIRGCGRTDFQEGSSDQLYESVHSQIFTLPKDTLIYPAHDYKGFEVSTVGEEMQHNPRLTKDKETFKTIMSNLNLSYPKMIDVAVPANMVCGLQDVPSQAN*

>ATGLX2-4

MQAISKVSSAASFFRCSRKLVSQPCVRPCVRQLHVRKGLVSGVMKLFSSPLRTLRDAGKSVRISRFCSVSNVSSSLQIELVPCLTDNYAYILHDEDTGTVGVVDPSEAVPVMDALQKNSRNLTYILNTHHHYDHTGGNLELKDRYGAKVIGSAADRDRIPGIDVALKDADKWMFAGHEVHIMETPGHTRGHISFYFPGARAIFTGDTLFSLSCGKLFEGTPEQMLASLQRIIALPDDTSVYCGHEYTLSNSKFALSIEPTNEVLQSYAAYVAELRDKKLPTIPTTMKMEKACNPFLRTENTDIRRALGIPETADEAEALGIIRRAKDNFKA*

>ATGLX2-5

MQTISKASSATSFFRCSRKLSSQPCVRQLNIRKSLVCRVMKLVSSPLRTLRGAGKSIRVSKFCSVSNVSSLQIELVPCLKDNYAYILHDEDTGTVGVVDPSEAEPIIDSLKRSGRNLTYILNTHHHYDHTGGNLELKDRYGAKVIGSAMDKDRIPGIDMALKDGDKWMFAGHEVHVMDTPGHTKGHISLYFPGSRAIFTGDTMFSLSCGKLFEGTPKQMLASLQKITSLPDDTSIYCGHEYTLSNSKFALSLEPNNEVLQSYAAHVAELRSKKLPTIPTTVKMEKACNPFLRSSNTDIRRALRIPEAADEAEALGIIRKAKDDF*

>GmGLYII-1

RKDENPSHTNYSYLYVVVFVTNLQDNVKGCTDKVENGDKVSLGPDVTVLALLTPCHTQGHISYYVTGKEDEQPAVFTGDTLFIASCGKFFEETAEQMYQSLNVTLASLPKSTRVYRGHEYSVNNLQFAVTLEPDNLRIQKKLAWARNQWQAGQATIPSTIEDELETNPFMRVDLPEIQERVGCKSPVKALGEIRKQKDNWRG*

>GmGLYII-2

MKIYHVPCLRDNYSYLIVDKSTKEGAVVDPVEPQKVLEAANSHWVNLKLVLTTHHHGDHAGGNEKIKQLVPGIKVYGSLIDNVIGCTDKVENGDKESLGADIYILCLHTPCHTKGHISYYVTGKEEEQPAVFTGDTLFIADCGKFFKGTAEQMYQSLCVTLGSLPKPTRVYCGHGEKVGCKSPVEALRELRKLKDNWKG*

>GmGLYII-3

IVDESTKEGAVVDPVEPQKVLEAANSHGVNNLKLVLTTHHHGDHAGGNEKIKQLVLGMKVYGGSMDNIKGCTDKVENGDKMSLGADINILCLHTPCHTKGHISYCVTGKEEEVLRKEYNKLILKAMPKFIAGCGKFFEGTAEQIYQSLCVTLGSLPKPTRVYCGHEYAVRNLLFALTIEPDNLRIQQKLTWAKNQQQAGQSTIPSTIEEEMETNPFMRVNLPEIQGASLPVEALRELRKLKDKWKGVMELTNYCILHV*

>GmGLYII-4

MGDTKERSGFCVWPDARQLCLGKGLLYGFMRLFSIPLKTLRGASRSLRVNQFCSVVNLSSSLQIELVPCLRDNYAYLLHDVDTGTVGVVDPSEAAPIIDALSKKDLNLTYIMNTNHHPDHTGGNAELKERYGAKVIGSEIDKERIPGIDIYLSDGDNWMFAGHEVHILATPGHTEGHVSFYFPGSGAIFTGDTLFSLSCGKLLEGTPKQMLSSLKRIMSLPDDTSIYCGHEYTSSNSKFALSIEPENKELQSYAAHVANLRNKGLPTIPTTVKVEKACNPFLRTWSMEIRQKLNIATTADDAEALGVIQQAKDNF*

>GmGLYII-5

MNVLVFERSGFCVWPDARQLCLRKGLLYGFMRLFSIPLKTLRGASRSLRVDQFCSVVNLSSSLQIELVPCLRDNYAYLLHDVDTGTVGVVDPSEAAPIIDALSKKDLNLTYILNTNHHPDHTGGNAELKERYGAKVIGSEIDKERIPGIDIYLSDGDNWMFAGHEVHILATPGHTEGHVSFYFPGSGAIFTGDTLFSLSCGKLLEGTPEQMLSSLKRIMSLPDDTSIYCGHEYTLNNSKFALSIEPENKELQSYATHVSNLRNKGLPTIPTTLKVEKACNPFLRTWSIEIRQKLNIAATADDAEALGVIRQAKDNF*

>GmGLYII-6

MPIATKLYASNVTSTLNSKTNSNNGDLIYVILIVIPVIKLFLYSTIHNQNPTTSTLQSLSSIQPFWWQLLFMLQKMLRLHFTTALSHFASKASPFPLTPVSVTVSRAIVCNNPTRFRSQMGSFSTSSSSSSKLLFRQLFEKESSTYTYLLADASHPEKPALLIDPVDRTVDRDLSIIEQLGLKLVYAMNTHVHADHVTGTGLIKSKVPSVKSVISKASGATADLYVEPGDKVQIGDLFLEVRATPGHTKGCVTYVTGDAPDQPQPRMAFTGDTLLIRGCGRTDFQGGSSEQLYKSIHSQILTLPKSTLIYPAHDYKGFTVSTVGEELQNNPRITKDEETFKNIMGNLNLSYPKMIDIAVPANMVCGIQSNPKQAEAS*

>GmGLYII-7

MLHMFSKASSAMATFPCSRVKSGLCVWPDVRQLCFRKGMLYGFMRLFSTPLKTLRGASRSLRVTQFCSVANMSSSLQIELVPCLKDNYAYLLHDVDTGTVGVVDPSEAVPIIDALSRKNRNLTYILNTHHHHDHTGGNVELKARYGAKVIGSGTDKERIPGIDIHLNDGDKWMFAGHEVRVMDTPGHTRGHISFYFPGSGAIFTGDTLFSLSCGKLFEGTPQQMLSSLKKIMSLSDDTNIYCGHEYTLNNIKFALSIEPENEELQSYAAQVAYLRSKGLPTIPTTLKVEKACNPFLRTSSAAIRQSLKIAATANDAEALGVIRQAKDNF*

>GmGLYII-8

MRIHHIACLQDNYSYLIVDESTKEAAAVDPVEPEKVLEVASSHGLTLKFVLTTHHHWDHAGGNDKIKQLVPGIKVYGGSIENVKGCTDKVENGDKVSLGAEITILALHTPCHTQGHISYYVTGKEDEQPAVFTGDTLFIASCGKFFEGTAEQMYQSLNVTLASLPKSTRVYCGHEYSVNNLQFALTLEPDNLRIQQKLTWARNQRQAGQATIPSTIEDELETNPFMRVDLPEIQERVGCKSPVEALGEIRKQKDNWRG*

>GmGLYII-9

MLSKPSSAMPTFPSSMVRSGLCVWPNVRQLCFRKGILYGFMRLFSTPLKTLRGASRSLRVAQFCSVANMSSSLQIELVPCLKDNYAYLLHDVDTGTVGVVDPSEAVPVIDALSRKNRNLTYILNTHHHHDHTGGNVELKARYGAKVIGSGTDKKRIPGIDIHLNDGDKWMFAGHEVRVMDTPGHTQGHISFYFPGSGAIFTGDTLFSLSCGKLFEGTPQQMLSSLKKIMSLPDNTNIYCGHEYTLNNTKFALSIEPENEELQSYAAQVAYLRSKGLPTIPTTLKMEKACNPFLRTSSAAIRQSLNIAATANDAEALGGIRQAKDNF*

>GmGLYII-10

LRSQMCSFSTTSFSSSSSKLLFHQLFEKKSSTYTYLLADASHPEKPTLLIDPVDRTVDRDLSLIEQLGLKIVYTMNTHVHADHVTGTGLIKGKVPSVKSVISKASGATVDLYVEPGDKVHIGDLFLEVRATPGHTKGCVTYVTGDAPDQPQPRMAFTGDTLLIRGCGRTGFQIYTCSKLLEQRRKWVKV*

>GmGLYII-11

TGKKRIPAIDIHLNDGDKWMCAGHEVRVMDTPGHTQGHISFYFPGSGVIFTGDTFFNLSCGKLFEGTPQQVVLNCTCPFFLFFFF*

>GmGLYII-12

MATHRLALIIQNPSNDDEFLLVKQSRPPKFHDEEYDSFVDSDLWDLPSAQLNPLLAESEPPVELELAVSHSESQDVDLRKFDIRSALNEVFGQLGFGAVDGGGWKFHKYVKEAAFGPDLPVNTVFIVGKLVAAEDKDFRDSYRWKSVRSCLNWILEVKPHGDRVGPLVVIGLINESSISTKWKVPPAINYQEYPPGNIIIPMGSRTLRPFHTTNLVVFAPENVSNDSGENNFIVRGDALIVDPGCLSEFYGELEKIVTALPRRLVVFVTHHHPDHVDGLSVIQKCNPDATLLAHEKTMHRISRDVWSLGYTPVTGDEDIDIGGQRLRVIFAPGHTDGHMALLHANTHSLIVGDHCVGQGSATLDIKAGGNMSEYFQTTYKFLELSPHALIPMHGRVNVWPKQMLCGYLKNRRSREANIVKAIEGGAKSLFDIIVYVYSDVDRRAWIAASSNVRLHVDHLAQQHKLPKDFSIQKFKNTCGLHFLSRWIWAYGSGSLSHQIGKSPFLVAGVLAGIAGIAVLYCQRKFTK*

>MtGLYII-1.1

MTIEVLVLGAGQEVGKSCVIVKINGKRIMFDCGMHMRHTDHSRYPDFKKISDSGNFNDALDCIIITHFHLDHVGALAYFTEVCGYSGPVYMTYPTKALSPLMLEDYRKVMVDRRGEEEQFTSDHIAECMKKVIAVDLKQTVQVDEDLQIRAYYAGHVIGAAMFYVKVGDAEMVYTGDYNMTPDRHLGAAQIDRLRLDLLITESTYATTIRDSKYAREREFLKAVHKCVSGGGKVLIPTFALGRAQELRILLDDYWERMNLKVPIYFSSGLTIQANTYHKMLIGWTSQKIKDTYSTHNAFDFKNVHKFERSMLDAPGPCVLFATPGMLIGGFSLEVFKHWAPSEKNLVALPGYCMAGTVGHRLTSGKPTKVDTDPDTQIDVRCQIHQLAFSAHTDSKGIMDLVKFLSPKHVMLVHGDKPKMVSLKERIDSELGIPCSHPANNEIVTISSTQYVNAEASDTFTKNCLNPNFKFQKCSSMDTCNSTLIDRNLTPELQVEDERVADGVLVMENNNNKKAKIVHEDEILLMLDEKKHEV*

>MtGLYII-2.1

MGTSVQVTPLCGVYNENPLSYLVSIDSFNILIDCGWNDHFDPSLLQPLSRVASTIDAVLLSHPDTLHLAALPYAIKHLGLSAPVYSTEPVYRLGLLTMYDHFLSRKQVSDFDLFTLDDIDSAFQTVTRLTYSQNHHLSGKGEGIVIAPHTAGHLLGGTIWKITKDGEDVIYAVDFNHRKERHLNGTVLGSFVRPAVLITDAYNALNNQPYRRQKDKEFGDILKKTLRAGGNVLLPVDTAGRILELILMLESYWADENLNYPIYFLTYVASSTIDYVKSFLEWMSDSIAKSFEQTRENIFLLKNITLLVSKADLDNAPDGPKVVLASMASLEAGFSHDIFVEWGNDVKNLVLFTERGQFGTLARMLQADPPPKAVKVTVSKRVPLVGEELIAYEEEQNRIKKEEALKASLMKEEEFKASQGADNNAIDPMIIDTGNSQPSPEVAVPKNGGYRDVFIDGFVPPSSSVAPMFPCYENITEWDDFGEVINPDDYVIKEEDMDQAANNVGGDLNGKLDESAASLIFDTKPSKVISDERTVQVRCSLVYMDFEGRSDGRSIKNILSHVAPLKLVLVHGSAEATDHLKQHCLKNVCPHVYAPQIEETIDVTSDLCAYKVQLSEKLMSSVLFKKLGEYEVAWVDAEAGKTENDMLSLLPVSGAPHPHKSVLVGDLKLADFKQFLSTKGVPVEFAGGALRCGEYVTVRKVGDATQKGAGSGTQQIIIEGPLCEDYYKIRDYLYSQFYLL*

>MtGLYII-3.1

MATHKLALIIQNPSNQNEFLLIKQSRPPKFNDEEYDSFLDSDLWDLPSVQLNPLQPQSDPPVEVQISVSHSDEFNFSEFDIHSALKEVFGELGFGIVERGEWKFHKYVKEPAFGPGLPVNTVFIAGKLVDDEIKDFSDSYKWMSIQSCLNWLLEVIPHGDRVGPLVVVGLVNDSSVSANWEAPPAINYQEYPTGVILIPMGSRTAKPFHTTNLVVFAPENVPNASKDNQLIVYGDALIVDPGCLSKFHGELKNIVTALPRRLVVFVTHHHRDHVDGLSVIQKCNPDAILLAHENTMRRISRDDWSLGYTSVTGDEDIYIGGQKLKVIFAPGHTDGHMALLHVNTHSLIVGDHCVGQGSALLDINSGGNMSEYFETTYKFLELSPHALIPMHGRVNVWPKQMLCEYLKNRRSREAAILKAIEGGAKTLFEIVAYVYSNVDRRAWIPASSNVRLHVDHLAEQHKLPKEFSIRNFKNTCGLHFLSRWIWGYTSCSIHPRKSSFLIAGVLVGIAVLVHCSAKTKFRK*

>MtGLYII-4.1

MSSVKKRESNGGTINRETEDQLIVTPLGAGNEVGRSCVYMTYKGKTVLFDCGIHPGYSGMAALPYFDEIDPSTVDVLLITHFHLDHAASLPYFLEKTTFKGRVFMTYATKAIYKLLLSDYVKVSKVSVDDMLYDEQDINRSMDKIEVIDFHQTVEVNGIRFWCYTAGHVLGAAMFMVDIAGVRVLYTGDYSREEDRHLRAAETPQFSPDVCIIESTYGVQHHQPRHTREKRFTDVIHSTISQGGRVLIPAYALGRAQELLLILDEYWANHPELQNIPIYYASPLAKKCLTVYETYTLSMNDRIQNAKSNPFAFKHISALSSIDIFKDVGPSVVMASPGGLQSGLSRQLFDMWCSDKKNSCVIPGYVVEGTLAKTILNEPKEVTLMNGLSAPLHMQVHYISFSAHADSAQTSAFLEELNPPNIILVHGAANEMGRLKQKLMTQFADRNTKILTPKNCQSVEMYFNSQKMAKTIGKLAEKTPEVGETVSGLLVKKGFTYQIMAPDDLHVFSQLSTANVTQRITIPYSGAFCVIQSRLKQIYESVEPSVDEESGVPMLLVHDRVTVKHESEKHVSLHWASDPINDMVSDSVVALVLNINRDLPKIVAESDATKIEEENEKKTEKVMQALLNSLFGNVKVGENGKLIINIDGNVAELNKESGEVESENEGLKERVRTAFRRIQSSVKPIPLSAP*

>MtGLYII-5.1

MATSNGTDDGTPPSESALIFLGTGCSSMVPNVLCLINPSDPPCSVCAQSLSIPPEKNPNYRCNTSMLIDYCGSGSNHNYILIDVGKTFRETVLRWFVHHRIPKIDSIILTHEHADAVLGLDDVRAVQPFSPTNDIDPTPIYLSQHSMDSIEEKFPYLVQKQRKEGQEIRRVAQMAWNIITDDCNQPFFASGLKFTPLPVMHGEDYICLGFLFGEKSRVAYISDVSRIPASTEYVISKSGAGQLDLLILDSLYRTGSHNVHLCFPQTLEIVKRLCPKQTLLIGMTHEFDHHKDNEFLKEWSRREGIPVQLSHDGLRVPINL*

>MtGLYII-6.1

MLKSQFIKFTPFFPYKPSFSSLSISTTIKLKSQMASYSTSSSSSKLLFRQLFEKESSTYTYLLADASHAEKPAVLIDPVDRTVDRDLSLIQELGLKLVYAMNTHVHADHVTGTGLIKSKVPDVKSVISKASGATADLYVEQGDKIRFGDLFLEVRATPGHTLGCLTYVTGDGPDQPQPRMAFTGDTLLIRGCGRTDFQGGSAEKLYKSIHSQIFTLPKDTLLYPAHDYKGFSVSTVGEEMQYNPRLTKDEETFKNIMANLNLSYPKMIDVAVPANMVCGVQSKTS*

>MtGLYII-7.1

MLSKASTTAMSAFSSCSRVRTGFSVWPNVRQLCFRKGILYGFMRLFSTPYKTLRGGASRSLRVARFCSVANMSSSLQIELVPCLSDNYAYILHDIDTGTVGVVDPSEATPVIDALSKKNRNLNYILNTHHHHDHTGGNVELKARYGAKVIGSATDKERIPGIDIHLNDGDKWMFAGHEVQVMDTPGHTRGHISFYFAGSGAIFTGDTLFSLSCGKLFEGTPQEMQSSLGKIMSLPDDTSIYCGHEYTLNNTDFALKLEPGNKELRSYAGHVASLRSKGLPTIPTTLKMEKACNPFLRTSNAQIRQLLNIPATADDAEALGIIRQAKDNF*

>MtGLYII-8.1

MAQILNFRNFLFLPSYKPTTHFRLRFLSTLVSSSSRRSNINAPPLHLRRRSTTTSTTPMEVEENSSVGFNKRRAEGTENSGLPKKNLQLKVRKLNPINTISYVQVLGTGMDTQDTSPAVMLFFDKQRFIFNAGEGLQRFCTEHGIKLSKIDHIFLSRVCSETAGGLPGLLLTLAGMGDEGMTVNVWGPSDLKYLVDAMRSFIPNAAMVHTKSFGPTFGTESTVKSQSDPIVLVDDEVVKISAIILQPCQIPSQKTDHSIDIADSLNGKKLLAAKPGDMSVVYVCELPEIQGKFDPEKAKALGLRPGPKYRELQLGNSVESDRQKNVMVHPSDVMDPSIPGPVVLVVDCPTESHLEALLSAKSLDTYGDQVGNLPKAGKSVSCVIHLTPESVVCCSNYQNWMKTFSSAQHIMAGHEKKNIEVPILKASARIATRLNYLCPRFFPAPGFWSLPNQNCSKPVSLASSEDSFSAPSNVIYAENLLKFTLRPYVNLGLDRSCIPPKASSSEIIDELLLEIPEVVEAAQHVRQLWEDSSQAKEDSIPLADHSEVIEEPWLSEDGITPACLENIRRDDLEIVLLGTGSSQPSKYRNVTSIYINLFSKGGLLLDCGEGTLGQLKRRYGVSGADDVVRSLSCIWISHIHADHHTGLTRILALRRDLLKGVPHEPVLVVGPRMLKRYLDAYHRLEDLDMLFLDCKHTFEASLADFENDLQETVNSLDLNNNNAEINASKVDSTLFARGSPMQSLWKRPGSPVDKDTVYPLLRKLKGVIQEAGLNTLISFPVVHCSQSYGVVLEAEKRINSVGKVIPGWKIVYSGDTRPCPELIKASRDATVLIHEATFEEGMVLEAIARNHSTTNEAIETGEAANVYRIILTHFSQRYPKIPVINKEHMDITCIAFDLMSINIADLPVLPKVLPYLKLLFRNDMTVDESNDVVVTVDESDDVVDVATSAS*

>MtGLYII-9.1

MATLTSLPPLPHSLLSLRSKPTRLSVSASALSASGNDGSTSRVPQKRRRRIEGPRKSMEDSVQRRMEQFYEGNDGPPLRVLPIGGLGEIGMNCMLVGNHDRYILIDAGIMFPDYDDLGVQKIIPDTTFIRKWSHKIEALVITHGHEDHIGALPWVIPALDSNTPIFASSFTMELIKKRLKEHGIFLPSRLKIFRTKNKFVAGPFEIEPIRVTHSIPDCCGLVLRCSDGTILHTGDWKIDETPLDGKVFDREGLEELSKEGVTLMMSDSTNVLSPGRTTSESVVADSLLRHISASKGRVITTQFASNLHRIGSVKAAADLTGRKLVFVGMSLRTYLEAAWKDGKAPFDPSTLVKAEDIDAYAPKDLLIVTTGSQAEPRAALNLASFGSSHAFKLTKEDIVLYSAKVIPGNESRVMEMMNRISEIGSTIVMGRNENLHTSGHAYRGELEEVLRIVKPQHFLPVHGEYLFLKEHESLGKSTGIRHTAVIKNGEMLGVSHLRNRRVLSNGFISLGKENLQLKYSDGDKAFGTSGELFLDERMRIALDGIIVVSMEIFRPKNLESLAGNTLKGKIRITTRCLWLDKGKLLDALYKAAHAALSSCPVKSPLPHMERTVSEVLRKMVRKYSGKRPEVIAIAIENPGAVFADEINTKLSGKSQVGPGISTFRRSVDEHRKENQSTALQIRDDGIDIEGLLVEIETITTAAEGDLSDSGESDEFWKPFIASSVEKSIKANNGYVSRKEHKSNTKQDDSEDIDEAKSEEMSDSEPESSKSEKKNKWKTEEVKKLIDLRSDLRDRFKVVKGRMALWEEISQSLLADGISRSPGQCKSLWTSLALKYEEIKNGKDSRKNWQYLEDMERILSSDETPATN*

>MtGLYII-10.1

MTDIKERSVLCALPDVRQICFRKGLLYGFSRVFSIPLKTLRGASRSLRVDQFCSVVNISSSLMIELVPCLRDNYAYILYDVDTGTVGVVDPSEAAPVIDALTKKNLNLTYILNTHHHHDHTNGNTELKERYGAKVIGSDVDKERIPGIDIYLSDGDKWMFAGHEVQIMATPGVTQGHISFYFPGSAAIFTGDTLFSLSCGKIYEGTPEQMLSSLKKITSLSDDTSIYCGHEYTLDNSKFALSIDPQNKELQSYASHVAQLRNKGLPTVPTTLKMEKACNPFLRTWSMEIRRKLKVAASADDAEALGVIRQAEDNF*

>MtGLYII-11.1

MVTCNSISLSHNLHFYTRFHRLHPTRRSHYCRFRSNALPRDTDGAKVVHKRPRRIEGPRKSMEDSVQRKMEQFYEGSDGPPLRVLPIGGLGEIGMNCMLVGNHDRYILVDAGVMFPGDDELGVQKIIPDTTFIKKWSHKIEAVVITHGHEDHIGALPWVIPMLDSQTPVFASSFTMELIRKRLKDHGIFVPSRLKVFRTRKKFVAGPFEIEPITVSHSIPDCCGLVLRCSDGTILHTGDWKIDETPLDGKVFDREALEELSKEGVTLMMSDSTNVLSPGRTMSESVVADALLRHISAAKGRVITTQFSSNIHRLGSLKAAADLTGRKLVFAGMSLRTYLDAAWKDGKVPIDSSTLVKVEDMHAYAPKDLLIVTTGSQAEPRAALNLASYGSSHAFELTKEDTVLYSAKVIPGNESRVMEMLNRISEIGPTIVMGKNECLHTSGHAYRGELEEVLRIVKPQHFLPIHGELLFLKEHELLGKSTGIRHTAVIKNGEMLGVSHLRNRKVLSNGFISLGKENLELKYSDGDKAFGTSSELFIDERLRIALDGIIVVSMEVCRAQSLDSSVENTLKGKIRITTRCLWLDKGKLLDALHKAAHASLSSCPVNCPLAHMEKTVSEMLRKMVRKYSGKRPEVIAVAIENPGAVLATEINTKLSGKSYVGGISTFRNVVHKENQSTKMQMRGMIGMLEFWRRSRMRRTERRHGYIWRTWKAFDNEALAKK*

>MtGLYII-12.1

MKIYHVPCLEDNYSYLIVDESTKEAAAVDPVEPEKVLEASNSLGLTIKFVLTTHHHWDHAGGNEKIKELVPGIKVYGGSIDNVKGCTNALENGDKVHLGADINILALHTPCHTKGHISYYVTGKEDEDPAVFTGDTLFIAGCGKFFEGTAEQMYQSLSVTLGSLPKPTRVYCGHEYSVKNLQFALTVEPDNLRILEKLTWAQNQRQTGQPTIPSTIGDELESNPFMRVDLPAIQEKMGFNSPVEALGELRKVKDNWRG*

>MtGLYII-13.1

MVVTSSIRLLPSSLTSLVHHRSPYSRRLLRPSSISFPLSPIHSLSSNGIGEVDSHVDQSQVIFIGTGTSEGIPRVSCLTNPSTKCPVCTKAAKPGDKNRRLNTSILVRHSNGTGTHNILIDAGKFFYHSALQWFPKFGIRTLDAVIITHSHADAIGGLDDLRDWTNNVQPSIPIYVAKRDFEVMKKTHYYLVDTSVIIPGAAVSALQFNSISEEPFFVHGLKFTPLPVWHGQGYRSLGFRFGNICYISDVSEIPEETYPLLKDCELLIMDALRPDRSSATHFGLPRALEEVRKIQPKRTLFTGMMHLMDHEEVNDYLTKLLESEGLDAQLSYDGLCIAVRL*

>MtGLYII-14.1

MKNLNWQLGGLRIVALSDGTHPFPVDTVFRDISKDDIRRDLDRAFLEPPVQGSINAFLVDTGTKRILVDSGAGVLYGDCCGKLLANLRAAGYAPEQIDEVLLTHLHKDHVGGIVTNGRMTFPNAVVRVNEIEANYWLDPDNKAQAPAFLASFFDAAAASVAPYIAAGRFRTFRGEATLAPGIRAVPMPGHTPGHTAYLIESGDAGLLAWGDIVHVAAIQLQDPDATVQYDSDADAARRTRRDTLKRVANKRYLVGAAHIAFPGLGHLRRDGEQYDWVPVNYDATPLR*
