# Supplementary material for: From methylglyoxal to pyruvate: a genome-wide study for the identification of glyoxalases and D-lactate dehydrogenases in Sorghum bicolor
Source: BMC Genomics. 2020 Feb 10;21:145. doi: 10.1186/s12864-020-6547-7 (PMC7011430; doi:10.1186/s12864-020-6547-7)
Supplement: Supplementary file 11 — Additional file 11. D-LDH protein sequences used for the phylogenetic analysis. [file 12864_2020_6547_MOESM11_ESM.docx]

>OsDLDH

MATAAAALLRLSRSRRPLLPLSSLRLPPPAPYHHHSHSQTPPSSSSSHARLPAFLSFLAAAAAAGGTTVALCDSGIDHRVGGKESTELVVRGERKRVPNEFIDELASFLGENLTVDYEERHYHGTPQNSFHKAVNVPDVVVFPRSQDEVQKIVMACNKYKVPIVPYGGATSIEGHTLAPHGGVCINMSLMKKIKSLHVEDMDVVVEPGVGWIELNEYLKPYGLFFPLDPGPGATIGGMCATRCSGSLAVSL*

>AtDLDH

MAFASKFARSKTILSFLRPCRQLHSTPKSTGDVTVLSPVKGRRRLPTCWSSSLFPLAIAASATSFAYLNLSNPSISESSSALDSRDITVGGKDSTEAVVKGEYKQVPKELISQLKTILEDNLTTDYDERYFHGKPQNSFHKAVNIPDVVVFPRSEEEVSKILKSCNEYKVPIVPYGGATSIEGHTLAPKGGVCIDMSLMKRVKALHVEDMDVIVEPGIGWLELNEYLEEYGLFFPLDPGPGASIGGMCATRCSGSLAVRYGTMRDNVISLKVVLPNGDVVKTASRARKSAAGYDLTRLIIGSEGTLGVITEITLRLQKIPQHSVVAVCNFPTVKDAADVAIATMMSGIQVSRVELLDEVQIRAINMANGKNLTEAPTLMFEFIGTEAYT

REQTQIVQQIASKHNGSDFMFAEEPEAKKELWKIRKEALWACYAMAPGHEAMITDVCVPLSHLAELISRSKKELDASSLLCTVIAHAGDGNFHTCIMFDPSSEEQRREAERLNHFMVHSALSMDGTCTGEHGVGTGKMKYLEKELGIEALQTMKRIKKTLDPNDIMNPGKLIPPHVCF*

>SorbicDLDH-1

MATSLLRLSRPRRALPLLPISSLRQPLSTQSHAPSPTPSSARRLPHFLSFLAAAAAAAAAGGATVALCDSGLDHHRVGGKDSTDLVVRGERKLVPQEFIDELASFLGDNMTLDYEERSFHGTPQNSFHKAINVPDVVVFPSSQDEVQKIVMACNKYKVPIVPYGGATSIEGHTLAPHGGVCIDMTLMKKIKSLNVEDMDVVVEPGVGWIELNEYLKPYGLFFPLDPGKNLAIVGNCIFTCFEGFKCMFSSHYHSVERSIEPSSYLEDGPGATIGGMCATRCSGSLAVRYGTMRDNVINLRAVLPNGDVVKTGSRARKSAAGYDLARLIIGSEGTLGVITEVTLRLQKLPSHSVVAMCNFKTIKDAADVAIATMLSGIQVSRVELLDEVQIKAINMANGKNLPEVPTLMFEFIGTEAYALEQTLLVQKIANKHHGSDFVFVEEPDAKAELWKIRKEALWAGFAMKPDYEAMITDVCVPLSRLAECISTSKRLLDASPLTCLVIAHAGDGNFHTIILFDPSQDDQQKEAERLNHFMVDTALSMEGTCTGEHGVGTGKMKYLEKELGIESLRTMKRIKGALDPNNIMNPGKLIPPHVCI*

>SorbicDLDH-2

MARREAARLLRRLGPLAVESPTRGMPRCQHESANHIVNSCRRFHWIPSLQRPLCGPTTCRGIYEGQSSANKACEVQKRTFGSAATATHIQRNPAYSQLSSDDVSYFKSVLGENGVVQDEDRVAVANVDWMGKYRGASQLLLLPKNTAEVSKILSYCNTRRLAVVPQGGNTGLVGGSVPVYDEVIVGLAGMDKIISFDNVNGILTCEAGCVLESLSNFVENEGFIMPLDLGAKGSCHIGGNVSTNAGGLRFIRYGSLHGSVLGLEVVLADGTILDMLTTLRKDNTGYDLKHLFIGSEGSLGVVTKISVLTPAKLPSTNVAFLSCNDYKSCQKLLLAARRSLGEILSAFEFMDHHCINLAMRHLEGVHNPLPASPYKFYVLIETTGSDESYDKTKLEAFLLRSMEDGLVADGVIAQDISQASNFWRIREGISEASVKVGAVYKYDLSIPVEKLYDIVEEMRCRLGDSAEVLGYGHLGDGNLHLNIVSSKYDDSTLGRIEPFVYEWTSAQRGSISAEHGLGLMKAEKIHYSKSPEAVQLMASIKKLLDPNSILNPYKVLPQSVL*

>SorbicDLDH-3

MRNVDYKRARHFQVDLSAFRNILEIDTERMVAKVEPLVSMGQITKATCPMNLSLAVAPEFDDLTVGGLINSYGISGGSHIYGLFTDTVVAMEVVLADGQVVRATMDNEHSDLFLWHAMVPRHDWAPCFSRDQAHSCQGIHEAHIYTPVRGTLKEIAEAYADSFVPRDGDPAKVPDFVEGMVYSSSEGVTMTGVYASEEEAKKKGNRINRVGWWFKPWFYQYAETALKRGEFVEYIPTREYYHRHTRSLYWEGKLIIPFGDQFWFRFLLGWLMPPKISLLKITQGEAIRNYYHDNHVIQDVLVPLHKVSDALEFAHRELEVYPVWLCPHRLYKLPVKTMVHPEPGFEQHRRKGDTSYAQMFTDVGFYYAPASVLRGEEFNGAEAVHRLEQWLIRNHGYQAQYAVSELSEKDFWRMFDPSHYEHCRRKYGAVGSFMSAHYKSKKRQKERGGGARS*

>SorbicDLDH-4.1

MDSRLRQSLLEKHPPPMADVHEPLVRRKRKKVLVDYLVQFRWILVIFVVLPISSLIYFNIFLGDMWSAMKSEKKRQKQHDENVQKVVKRLKQRNPKKDGLVCTARKPWIAVGMRNVDYKRARHFEVDLSSFRNILEIDKERMVAKVEPLVNMGQITRATCPMNLALAVVAELDDLTVGGLINGYGIEGSSHLYGLFSDTVVAMEVVLADGRVVRATKDNEYSDLFYGIPWSQGTLGFLVSAEIKLIPIKEYMKLTYIPVKGSLKEIAQAYADSFAPRDGDPAKVPDFVEGMVYTESEGVMMTGVYASKEEAKKKGNKINCVGWWFKPWFYQHAQTALKRGEFVEYIPTREYYHRHTRCLYWEGKLILPFGDQFWFRFLLGWLMPPKVSLLKATQGEAIRNYYHDNHVIQDMLVPLYKVGDALEFVHREMEVYPLWLCPHRLYKLPVKTMVYPEPGFEHQHRQGDTSYAQMFTDVGVYYAPGAVLRGEEFNGAEAVHRLEQWLIENHSYQPQYAVSELNEKDFWRMFDASHYEHCRHKYGAVGTFMSVYYKSKKGRKTEKEVQEAEAAILEPAYADEA*

>SorbicDLDH-4.2

MADVHEPLVRRKRKKVLVDYLVQFRWILVIFVVLPISSLIYFNIFLGDMWSAMKSEKKRQKQHDENVQKVVKRLKQRNPKKDGLVCTARKPWIAVGMRNVDYKRARHFEVDLSSFRNILEIDKERMVAKVEPLVNMGQITRATCPMNLALAVVAELDDLTVGGLINGYGIEGSSHLYGLFSDTVVAMEVVLADGRVVRATKDNEYSDLFYGIPWSQGTLGFLVSAEIKLIPIKEYMKLTYIPVKGSLKEIAQAYADSFAPRDGDPAKVPDFVEGMVYTESEGVMMTGVYASKEEAKKKGNKINCVGWWFKPWFYQHAQTALKRGEFVEYIPTREYYHRHTRCLYWEGKLILPFGDQFWFRFLLGWLMPPKVSLLKATQGEAIRNYYHDNHVIQDMLVPLYKVGDALEFVHREMEVYPLWLCPHRLYKLPVKTMVYPEPGFEHQHRQGDTSYAQMFTDVGVYYAPGAVLRGEEFNGAEAVHRLEQWLIENHSYQPQYAVSELNEKDFWRMFDASHYEHCRHKYGAVGTFMSVYYKSKKGRKTEKEVQEAEAAILEPAYADEA*
