# Supplementary material for: From methylglyoxal to pyruvate: a genome-wide study for the identification of glyoxalases and D-lactate dehydrogenases in Sorghum bicolor
Source: BMC Genomics. 2020 Feb 10;21:145. doi: 10.1186/s12864-020-6547-7 (PMC7011430; doi:10.1186/s12864-020-6547-7)
Supplement: Supplementary file 3 — Additional file 3: Figure S3. Domain sequence alignment of predicted GLYI proteins from sorghum and rice. Conserved metal binding sites have been marked with an asterisk and region specific for Zn2+-dependent isoforms has been marked with a red line. Each domain of the two glyoxalase domain (PF00903)-containing putative GLYI proteins has been represented as a separate sequence, with domains being indicated by numbers 1 or 2 suffixed to the protein name thereby, indicating first and second domain, respectively. [file 12864_2020_6547_MOESM3_ESM.pdf]

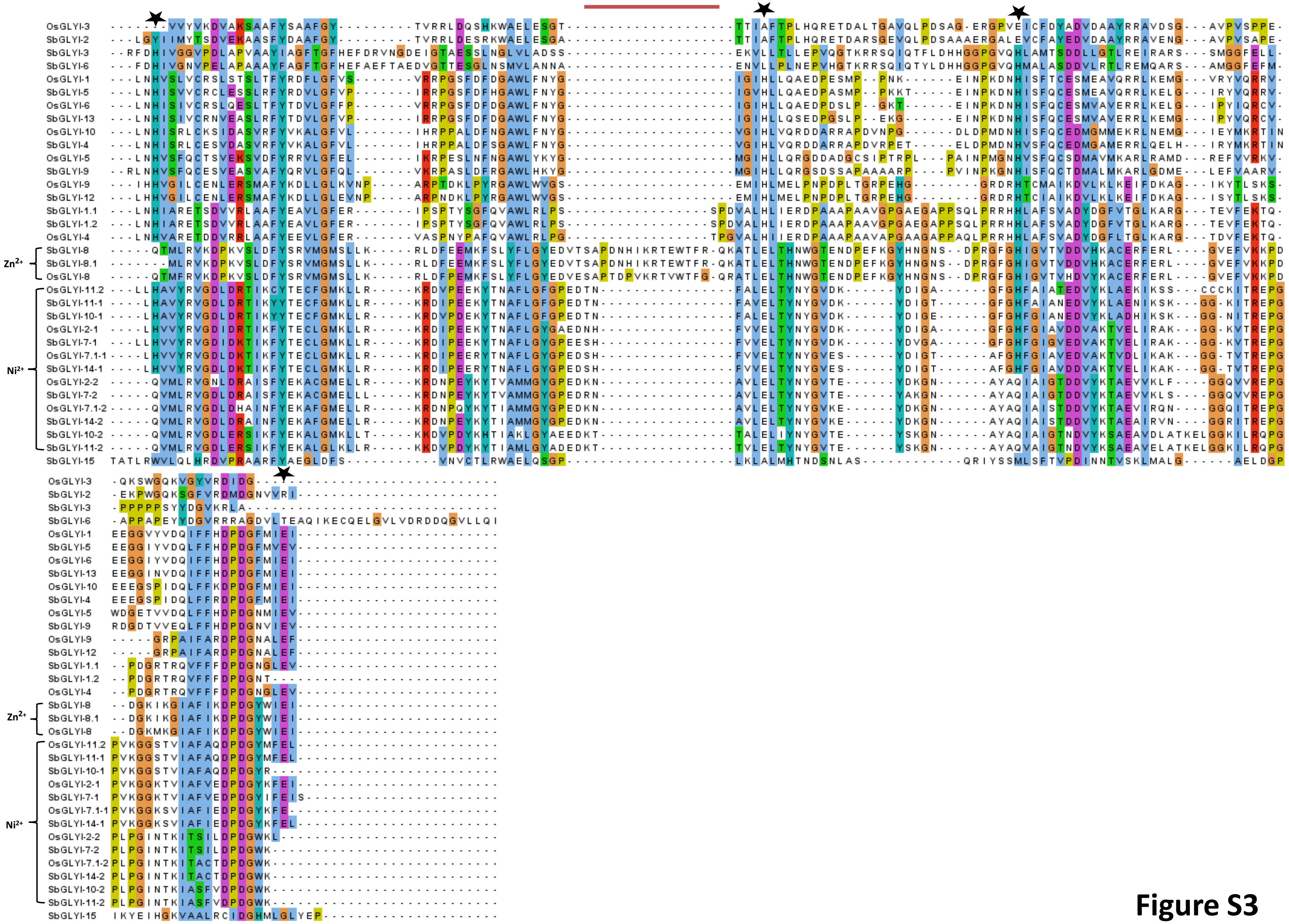
OsGLYI-3
SbGLYI-2
SbGLYI-3
SbGLYI-6
OsGLYI-1
SbGLYI-5
OsGLYI-6
SbGLYI-13
OsGLYI-10
SbGLYI-4
OsGLYI-5
SbGLYI-9
OsGLYI-9
SbGLYI-12
SbGLYI-1.1
SbGLYI-1.2
OsGLYI-4
Zn2+
SbGLYI-8
SbGLYI-8.1
OsGLYI-8
Ni2+
OsGLYI-11.2
SbGLYI-11-1
SbGLYI-10-1
OsGLYI-2-1
SbGLYI-7-1
OsGLYI-7.1-1
SbGLYI-14-1
OsGLYI-2-2
SbGLYI-7-2
OsGLYI-7.1-2
SbGLYI-14-2
SbGLYI-10-2
SbGLYI-11-2
SbGLYI-15

Figure S3
